# The dual role of POSTN in maintaining glioblastoma stem cells and the immunosuppressive phenotype of microglia in glioblastoma

**DOI:** 10.1186/s13046-024-03175-9

**Published:** 2024-09-04

**Authors:** Hao Wang, Lin Yao, Jinming Chen, Yanyan Li, Zuopeng Su, Yongsheng Liu, Wen Li, Yun Xiong, Heyang Gao, Xiao Zhang, Youxin Zhou

**Affiliations:** 1 Department of Neurosurgery & Brain and Nerve Research Laboratory, The First Affiliated Hospital of Soochow University, Suzhou, 215006 China; 2 Department of Neurosurgery, The First Affiliated Hospital of Gannan Medical University, Ganzhou, 341000 China; 3 Department of Neurosurgery, Minhang Hospital of Fudan University, Shanghai, 201199 China

**Keywords:** Glioblastoma, POSTN, Glioblastoma stem cell, Microglia, Regulatory T cell, Immunosuppressive microenvironment

## Abstract

**Background:**

Glioblastoma (GBM) is an immunosuppressive, universally lethal cancer driven by glioblastoma stem cells (GSCs). The interplay between GSCs and immunosuppressive microglia plays crucial roles in promoting the malignant growth of GBM; however, the molecular mechanisms underlying this crosstalk are unclear. This study aimed to investigate the role of POSTN in maintaining GSCs and the immunosuppressive phenotype of microglia.

**Methods:**

The expression of POSTN in GBM was identified via immunohistochemistry, quantitative real-time PCR, and immunoblotting. Tumorsphere formation assay, Cell Counting Kit-8 assay and immunofluorescence were used to determine the key role of POSTN in GSC maintenance. ChIP-seq and ChIP-PCR were conducted to confirm the binding sequences of β-catenin in the promoter region of FOSL1. Transwell migration assays, developmental and functional analyses of CD4^+^ T cells, CFSE staining and analysis, enzyme-linked immunosorbent assays and apoptosis detection tests were used to determine the key role of POSTN in maintaining the immunosuppressive phenotype of microglia and thereby promoting the immunosuppressive tumor microenvironment. Furthermore, the effects of POSTN on GSC maintenance and the immunosuppressive phenotype of microglia were investigated in a patient-derived xenograft model and orthotopic glioma mouse model, respectively.

**Results:**

Our findings revealed that POSTN secreted from GSCs promotes GSC self-renewal and tumor growth via activation of the αVβ3/PI3K/AKT/β-catenin/FOSL1 pathway. In addition to its intrinsic effects on GSCs, POSTN can recruit microglia and upregulate CD70 expression in microglia through the αVβ3/PI3K/AKT/NFκB pathway, which in turn promotes Treg development and functionality and supports the formation of an immunosuppressive tumor microenvironment. In both in vitro models and orthotopic mouse models of GBM, POSTN depletion disrupted GSC maintenance, decreased the recruitment of immunosuppressive microglia and suppressed GBM growth.

**Conclusion:**

Our findings reveal that POSTN plays critical roles in maintaining GSCs and the immunosuppressive phenotype of microglia and provide a new therapeutic target for treating GBM.

**Supplementary Information:**

The online version contains supplementary material available at 10.1186/s13046-024-03175-9.

## Background

Glioblastoma (GBM), classified as a grade 4 glioma, is the most prevalent intrinsic malignancy of the central nervous system [1]. Despite the implementation of the standard treatment protocol, which includes maximal surgical resection, radiotherapy and temozolomide (TMZ)-based chemotherapy combined with subsequent sequential TMZ treatment, the mean overall survival (OS) time of newly diagnosed GBM patients is only 14.6 months [2]. Glioblastoma stem cells (GSCs) are small populations of GBM cells with self-renewal and multilineage differentiation capabilities and are considered responsible for the tumorigenesis and development of GBM [3]. GSCs also exhibit radiation resistance, chemoresistance, and angiogenic and invasive properties, which are correlated with poor outcomes in GBM patients [3, 4]. Therefore, targeting GSCs constitutes a promising approach for treating GBM patients.

The mutual regulation between GSCs and the tumor microenvironment (TME) profoundly affects GBM development. The TME is composed of the extracellular matrix, blood vessels, immunocytes, secreted molecules and other components. The development of the TME is essential for GBM occurrence and development [5]. Previous studies have confirmed that GSCs interact with endothelial cells, astrocytes, fibroblasts, microglia and other immune cells in the TME to reshape the microenvironment, thereby inducing the malignant progression of GBM [6–8]. In the GBM microenvironment, microglia are among the most abundant immune cell populations [9]. The proliferative activity of microglia is significantly greater in GBM tissue than in normal brain tissue [10]. On the one hand, microglia synthesize and release transforming growth factor-beta 1 (TGF-β1) to support GSC invasion by promoting MMP-9 expression [11]; on the other hand, immunosuppressive microglia promote the formation of an immunosuppressive microenvironment in the GBM by releasing IL-10, regulating the mammalian target of rapamycin (mTOR) pathway or other pathways [12, 13]. Furthermore, CSCs recruit and regulate microglia by producing factors such as soluble colony-stimulating factor (sCSF-1), macrophage-inhibiting cytokine-1 (MIC-1), and Wnt1-inducible signaling pathway protein 1 (WISP1), leading to the establishment of an immunosuppressive TME [14, 15]. Accordingly, GSCs and their interactions with microglia may be potential targets for GBM therapy. In this study, POSTN was identified as a key therapeutic target that mediates GSC–microglia crosstalk.

POSTN is a secreted matricellular protein encoded by the POSTN gene located on chromosome 13q13.3 [16]. Under physiological conditions, POSTN is involved in wound healing, the formation and maintenance of tooth structure and normal bone, and the development of heart valves [16–19]. POSTN is expressed at extremely low levels in healthy tissues but is overexpressed in cancers, including GBM [20]. Tian et al. reported that POSTN expression is high in GSCs and may be an independent prognostic factor for glioma [21]. Zhou et al. confirmed that POSTN secreted by GSCs can recruit tumor-associated macrophages (TAMs). Silencing POSTN expression in GSCs reduced the recruitment of TAMs, inhibited tumor growth and increased the survival rate of mice transplanted with GSCs [22]. Castellani et al. confirmed that POSTN could mimic the effects of extracellular vesicles derived from endothelial cells on the migration and clonogenic ability of GSCs [23]. These findings show that POSTN facilitates the maintenance of GSCs and tumor-associated immune cells and that POSTN may be a therapeutic target for mediating GSC–immune cell crosstalk. However, the precise mechanisms by which POSTN regulates GSCs and the immune microenvironment remain to be clarified.

In this study, we showed that POSTN secreted from GSCs promotes GSC self-renewal and tumor growth via activation of the αVβ3/PI3K/AKT/β-catenin/FOSL1 pathway. In addition to its intrinsic effects on GSCs, POSTN can recruit microglia and upregulate CD70 expression in microglia through the PI3K/AKT/NFκB pathway, which in turn promotes Treg development and functionality and supports the formation of an immunosuppressive TME. Inhibition of POSTN disrupts GSC maintenance, inhibits the recruitment of immunosuppressive microglia, suppresses regulatory T-cell (Treg) development and function, and suppresses GBM growth, suggesting that targeting POSTN may effectively increase the efficacy of GBM treatment.

## Methods

### Cell culture

SHG141 GSCs, SHG142 GSCs, SHG143 GSCs, SHG144 GSCs, SHG145 GSCs and SHG146 GSCs were obtained from the GBM tissues of patients at the First Affiliated Hospital of Soochow University via CD133^+^ magnetic bead (Miltenyi) sorting and cultured in GSC medium. The GSC culture medium consisted of DMEM/F12 (Corning), B27 (Gibco), 20 ng ml^− 1^ recombinant human epidermal growth factor and 20 ng/ml basic fibroblast growth factor. All procedures performed using human tissues were approved by the Ethics Committee of Soochow University (Approval No. SUDA20221206H03). The murine glioma cell line GL261 was obtained from the American Type Culture Collection, and the BV2 cell line was purchased from Pricella Biotechnology Co., Ltd. and cultured in DMEM supplemented with 10% FBS (VivaCell) and 1% PS. The HMC3 cell line was purchased from Pricella Biotechnology Co., Ltd., and cultured in MEM (including NEAA) supplemented with 10% FBS (VivaCell) and 1% PS. All the cells were maintained at 37 ℃ and 5% CO_2_. We have included the clinical information of the analyzed glioma cells in Supplementary Table S1.

### Tumorsphere formation assay

The cells from the different treatment groups were resuspended in 96-well plates at 100 cells/well. After 10 days, the tumorspheres in each well were imaged and quantified, and the diameter was measured.

### Cell counting Kit-8 (CCK-8) assay

Cells from different treatment groups were seeded into 96-well plates at 1000 cells per well. On Days 0, 2, 4, and 6, 10 µl of CCK-8 (Beyotime) reagent was added to each well. After reacting at 37 ℃ for 2 h, the optical density at a wavelength of 450 nm was measured.

### Transwell migration assay

One hundred microliters of 5 × 10^4^ HMC3 cells suspended in serum-free medium were seeded in the chamber of a Transwell 24-well plate. (8.0 μm, Corning). Culture medium containing 10% serum with or without rhPOSTN (R&D) was added to the remaining wells. After 48 h, the migrated microglia were fixed, stained with crystal violet (Beyotime) and counted via ImageJ.

### Isolation of peripheral blood mononuclear cells from healthy donors

The collection of peripheral blood from healthy donors was approved and performed by the Ethics Committee of Soochow University (Approval No. SUDA20221206H03). All donors provided their formal consent for their blood to be used in the experiments and analyses described in this study. Four milliliters of lymphocyte isolation solution was pipetted into a 15 ml separation tube, the blood was diluted with PBS 1:1, 6 ml of diluted blood sample was added to the 15 ml separation tube, and the tube was centrifuged at 400 × g for 30 min at room temperature; after centrifugation, the upper plasma was removed before proceeding. PBMCs were collected in a new centrifuge tube, resuspended, washed with PBS, and centrifuged at 400 × g for 10 min at room temperature; this process was repeated twice.

### CD4+ naïve T-cell sorting

CD4^+^ naïve T cells were magnetically isolated from peripheral blood mononuclear cells via a naïve CD4^+^ T-cell isolation kit II (Miltenyi) following the manufacturer’s instructions. CD4^+^ naïve T cells were activated in TexMACS™ GMP medium (Miltenyi) supplemented with 10% heat-inactivated FBS, 1% PS, 50 µM 2-mercaptoethanol (Gibco), and 1:100 T-Cell TransAct™ (Miltenyi) for 2–3 days.

### Coculture of HMC3 cells and naïve CD4+ T cells

For indirect coculture, HMC3 cells were seeded in 24-well plates at a density of 5 × 10^4^ cells/well for 24 h, and then, 1 × 10^5^ CD4^+^ naïve T cells were added to Transwell chambers (1 μm, Corning). For direct coculture of naïve CD4^+^ T cells with HMC3 cells, HMC3 cells were seeded in 24-well plates at a density of 5 × 10^4^ cells/well and cultured for 24 h until the HMC3 cells adhered to the plate. After HMC3 cells adhered to the plate, the supernatant in the 24-well plates was discarded, and 2 × 10^5^ naïve CD4^+^ T cells were seeded into 24-well plates for direct coculture with HMC3 cells. The cells were cultured in 1.5 mL of TexMACS™ GMP medium (Miltenyi) supplemented with 10% heat-inactivated FBS, 1% PS, 25 IU mL^− 1^ IL-2 (Miltenyi), 2 ng mL^− 1^ TGFβ1 (PeproTech) and 50 µM 2-mercaptoethanol for 3 days.

### CFSE staining and analysis

CD8^+^ T cells were stained with CFSE (BD Biosciences) according to the manufacturer’s instructions before they were cocultured with CD4^+^ naïve T cells. After coculture for 48 h, the proliferation of CD8^+^ T cells was assessed by staining with a PE-CYN7-conjugated anti-human CD8 antibody (eBioscience), and the CFSE intensity in the CD8^+^ subpopulation was determined via a Beckman Cytomics flow cytometer and quantified via CFSE analysis via Kaluza software.

### Enzyme-linked immunosorbent assay (ELISA)

The levels of IL-10, TGF-β1, IFN-γ, Granzyme B, Perforin and sCD27 in the cell culture supernatants were measured via ELISA via commercial IL-10 (Invitrogen, #BMS215-2), TGF-β1 (Invitrogen, #BMS249-4), IFN-γ (Invitrogen, #KHC4021), Granzyme B (Invitrogen, #BMS2027-2), Perforin (Invitrogen, # BMS2306) and sCD27 (Invitrogen, #BMS286INST) kits following the manufacturers’ instructions. The OD value was measured with a Varioskan LUX multimode microplate reader.

### Adenosine assay

The cell supernatants were collected and cleared of cells and debris via centrifugation. The cleared supernatants were stored at -80 ℃. Extracellular adenosine levels were measured via a fluorescent adenosine assay (Abcam, Cat# ab211094) according to the manufacturer’s instructions.

### Apoptosis detection test

After GSCs were cocultured with CD8^+^ T cells and CD4 naïve T cells for 48 h, a PE Annexin V Apoptosis Detection Kit I (BD Biosciences) was used to determine the apoptosis rate of CD45-negative GSCs. The cellular intensities of PE-Annexin V, APC-7-AAD and FITC-CD45 were measured via a Beckman Cytomics flow cytometer and analyzed via Kaluza software.

### Developmental and functional analysis of CD4+ T cells

CD4^+^ naïve T cells were collected, washed with cell staining buffer (eBioscience) and centrifuged at 300 × g for 5 min. Subsequently, the T cells were fixed and permeabilized using FOXPЗ/TRH FACTOR STANBUFFER (Invitrogen). The nuclear factor FOXP3 and intracellular CTLA4 were stained with an AF488-conjugated anti-human FOXP3 antibody (eBioscience) and a PE-conjugated anti-human CTLA4 antibody (eBioscience), measured with a Beckman Cytomics flow cytometer and analyzed with Kaluza software.

### Immunoblotting

Protein expression in tissues and cells was determined by immunoblotting analysis as previously described [24]. Normal brain and glioma samples were derived from epilepsy surgery or tumor resection of glioma, respectively. All procedures performed using human tissues were approved by the Ethics Committee of Soochow University (Approval No. SUDA20221206H03). The primary antibodies used in this study are listed in Supplementary Table S2. Exposure was performed after secondary antibody incubation, and each experiment was repeated at least three times.

### Immunohistochemistry and immunofluorescence

The experimental procedures were performed as previously described [24], and the primary antibodies used in this study are listed in Supplementary Table S2.

### Quantitative real-time PCR

Total RNA was isolated from tissues and cells via TRIzol reagent (Beyotime). qRT‒PCR was performed by using an ABI Prism 7500 heat cycle apparatus with PowerUp™ SYBR™ Green (Thermo Fisher Scientific). The following quantitative PCR primers were used in this study: human POSTN, forward 5’-CCCCGTGACTGTCTATAAGCC-3’ and reverse 5’-TGACCTTGGTGACCTCTTCTTG-3’; human FOSL1, forward 5’-CAGGCGGAGACTGACAAACTG-3’ and reverse 5’-TCCTTCCGGGATTTTGCAGAT-3’; human CD70, forward 5’-GCTTTGGTCCCATTGGTCG-3’ and reverse 5’-CGTCCCACCCAAGTGACTC-3’; mouse CD70, forward 5’-CCGCACACAGCTGAGTTACA-3’ and reverse 5’-CTCTGGTCCGTGTGTGAAGG-3’; and human GAPDH, forward 5’-CATGAGAAGTATGACAACAGCCT-3’ and reverse 5’-AGTCCTTCCACGATACCAAAGT-3’.

### RNA-seq

After the extracted total RNA samples underwent quality control and were quantified via agarose electrophoresis and Nanodrop analysis, the mRNAs were enriched with olig (dT) magnetic beads, and a library was constructed via a KAPA Stranded RNA-Seq Library Prep Kit (Roche). The mixed sequencing libraries of different samples were denatured with 0.1 M NaOH to generate single-stranded DNA, which was subsequently diluted to a concentration of 8 pM and amplified in situ via a reagent kit. The ends of the generated fragments were sequenced for 150 cycles using a DNBSEQ-T7 sequencer. The original sequencing data were subjected to quality control and compared for statistical and quantitative analysis of gene and transcript expression. Solexa pipeline version 1.8 (Off-Line Base Caller software, version 1.8) software was used for image processing and base recognition. FastQC software was used to evaluate the sequencing quality of the reads, and then Cutadapt was used to remove 3’ and 5’ adapters. The reference genome was aligned with HISAT2 software. Transcript abundance was estimated via StringTie software with reference to the official database annotation information. R software Ballgown was used to calculate the FPKM at the gene level and transcript level, the expression differences at the gene level and transcript level were calculated, and differentially expressed genes between samples or groups were screened. New gene/transcript predictions were assembled and merged via StringTie for each sample and compared with the official annotation information obtained via Ballgown calculations, and the coding capacity of new transcripts was predicted via CPAT. rMATS was used for alternative splicing event detection, difference calculation and mapping. GO and KEGG enrichment analyses were performed via the R package ClusterProfiler v4.6.0.

### ChIP-seq and ChIP‒PCR

ChIP-seq experimental procedures and analysis were performed as described previously [24]. For ChIP‒PCR, DNA samples were immunoprecipitated via antibody-coupled magnetic beads on a rotator overnight at 4°C. The immunoprecipitates were collected via a magnetic stand. The beads were washed, and the bound chromatin was eluted in ChIP elution buffer. Chromatin products were treated with RNase A (37°C for 15 min) and proteinase K (55°C for 2 h). After DNA purification, FOSL1 or CD70 promoter binding sites were quantified via qPCR and normalized to total chromatin (input). The primers used for FOSL1 were as follows: FOSL1-1, forward 5’-GTCTCCGTGCCCGCTTTCT-3’ and reverse 5’-CTTCGAGGCGACCACCCTA-3’; FOSL1-2, forward 5’-ACAAGGCCAGTGGAAAGACC-3’ and reverse 5’-CACAAAATAGCACGAAAGAATCA-3’;

FOSL1-3: forward 5’-TTCTGGATGTGCGACAAGGT-3’ and reverse 5’-GCTGGGAGATGCTGAAATGAAT-3’;

FOSL1-4: forward 5’-AAGCATTACCTTATCGCAAACAT-3’ and reverse 5’-CCAAGTAAGTGGGACTACAGGC-3’;

FOSL1-5: forward 5’-GATGAAATGGAAGGACCGATG-3’ and reverse 5’-CTGGGGGACAGAGCAAGACT-3’. The primers used for CD70 were as follows: CD70-1, forward 5’-ACAGGTTGAAGCAAGTAGACGC-3’ and reverse 5’-GCCGAGAAGGAAGGAAGGAA-3’; CD70-2, forward 5’-AAGAATGAGGTGGAGAGGGGA-3’ and reverse 5’-AAAGTGGGTGGAGGCAGTTG-3’; CD70-3,

forward, 5’-ACAAAAAAGCAGGTGGTCTCC-3’ and reverse, 5’-GGCTGGACTTGAACTCCTGAC-3’; CD70-4,

forward, 5’-CGCTTGAGCCCAGATGTTT-3’ and reverse, 5’-GTCTACCAGCACACGCTACCAT-3’; and CD70-5,

forward, 5’-CCTTCTTGGTGTTTCTTGACTTTC-3’, and reverse, 5’-GTGGAGAGAAAAGAGGCTCATAGA-3’.

### Intracranial mouse model

All experimental protocols were conducted according to the Soochow University guidelines for animal research and were approved by the Ethics Committee of Soochow University (Approval No. SUDA20221206A02). Five-week-old female (SLAC Laboratory Animal Center; Shanghai, China) nude mice were used to establish intracranial GBM xenografts. Before the formal experiment began, all the nude mice were randomly divided into groups. Previously, SHG143 GSCs were infected with a POSTN knockdown lentivirus. Luciferase-labeled SHG143 GSCs or SHG143 GSCs + POSTN knockdown cells were used for the mouse xenograft model. A total of 5 × 10^5^ cells were intracranially injected into each mouse. Bioluminescence imaging was performed on days 7, 14, 21, and 28 to monitor intracranial tumor growth. At the end of the experiment, Kaplan–Meier survival curves were plotted to determine survival. Five-week-old female (SLAC Laboratory Animal Center; Shanghai, China) C57BL/6J mice were used to establish an intracranial model of glioma. Before the formal experiment began, all C57BL/6J mice were randomly divided into groups. GL261 cells or GL261 cells + POSTN-knockdown cells labeled with luciferase were used to establish a mouse intracranial model of glioma. A total of 5 × 10^5^ cells were intracranially injected into each mouse. Bioluminescence imaging was performed on days 7, 14, 21, and 28 to monitor intracranial tumor growth. At the end of the experiment, Kaplan–Meier survival curves were plotted to determine survival.

### Single-cell RNA-seq data analysis

scRNA-seq data was performed in the R environment (v4.3.1). The raw data for 2 samples were processed separately with the Seurat (v4.4.0) method of data cleaning. To remove low expressed genes and low-quality cells, we retained genes expressed in at least 3 cells and filtered the cells with more than 12% mitochondrial reads. Additionally, cells with fewer than 200 genes or more than 6000 genes, or fewer than 500 reads or more than 25,000 reads were removed. We then passed the Seurat object to the RunHarmony() function, RunUMAP() and FindNeighbors() to constructe a shared nearest neighbor graph with Harmony reduction and 50 dimensions input. The identified clusters were subjected to cell type identification using previously reported markers [25, 26]. Tumor clusters were then isolated and the clustering procedure was repeated to differentiate more specific subtypes for tumor cells. The also used scRNA-seq data of 3 GBM patients from GEO database under the accession number GSE139448, we processed the datasets using the same parameters as described above.

### Statistical analysis

Comparisons between two groups were performed via two-tailed unpaired Student’s t test, and multiple comparisons between groups were performed via one-way analysis of variance (ANOVA) with Tukey’s method. For Kaplan‒Meier survival curves, we used the “surv_cutpoint” function of the R package “survminer” to calculate the optimal cutoff value and divided the expression data into a high-expression group and a low-expression group. Log-rank tests were performed to assess the statistical significance of differences between groups. Correlation analyses were performed via the Pearson test to determine R and P values. In vitro and in vivo measurement data are expressed as the mean ± standard deviation (SD). Statistical analysis was performed via GraphPad Prism 9. Differences with a minimum *P* < 0.05 were considered statistically significant.

## Results

### POSTN is highly expressed in GBM and promotes GSC self-renewal

**Fig. 1:**
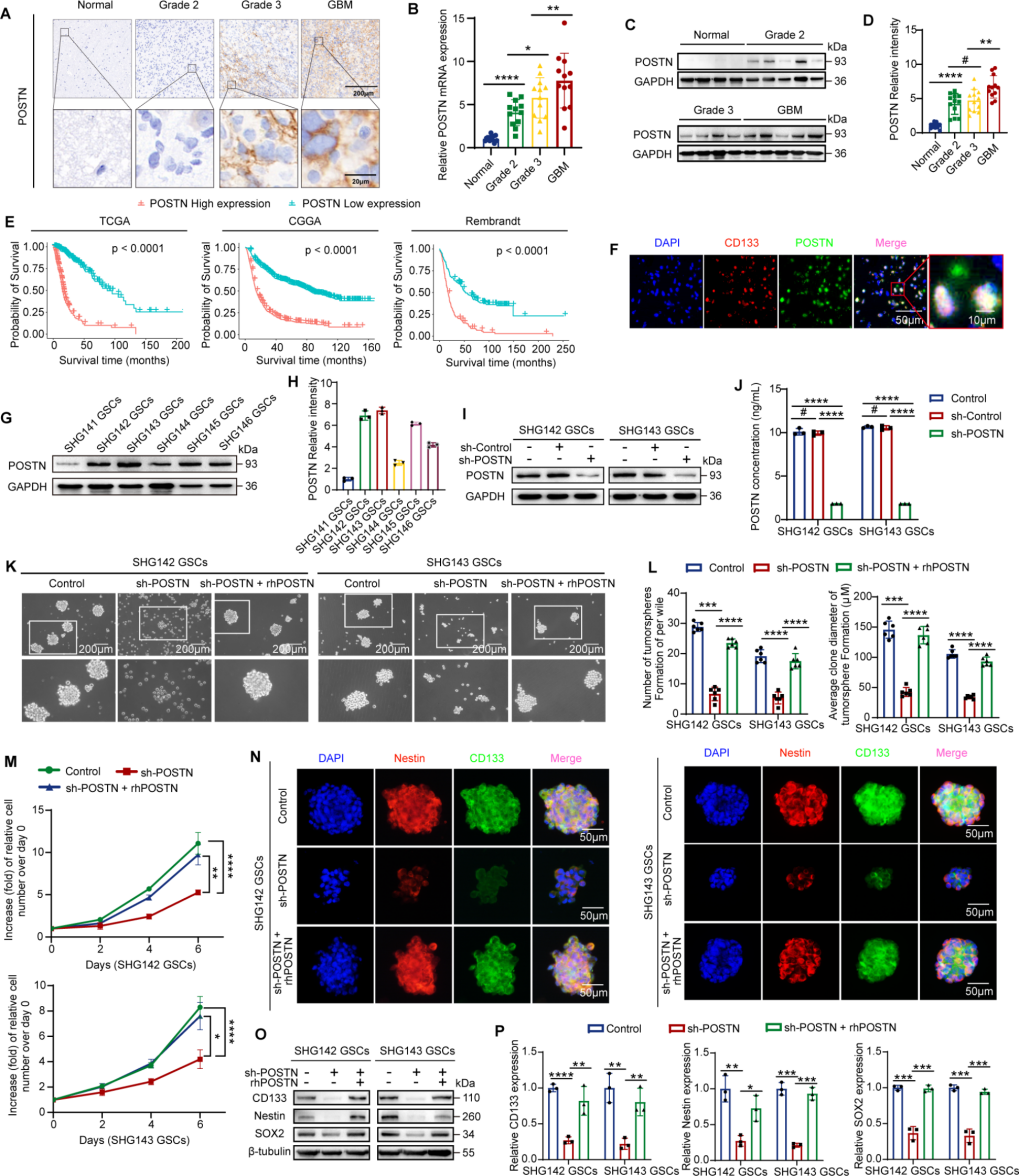
POSTN is highly expressed in glioblastoma and promotes glioblastoma stem cell self-renewal. **A**, Images showing the expression of POSTN in human normal brain tissue and glioma tissue samples via immunohistochemical staining. Scale bar, 200 μm (top), 20 μm (bottom). **B**, qRT‒PCR analysis of POSTN mRNA expression in human normal brain tissue and glioma tissue samples; *n* = 12 for all groups. **C** and **D**, Immunoblot analysis of POSTN in human normal brain tissue and glioma tissue samples. POSTN protein levels were quantified (**D**). **E**, Kaplan–Meier survival analysis of patients stratified by median POSTN expression in different glioma datasets: the TCGA, CGGA, and Rembrandt datasets. TCGA datasets: low POSTN expression, *n* = 279; high POSTN expression, *n* = 233. CGGA datasets: low POSTN expression, *n* = 557; high POSTN expression, *n* = 413. Rembrandt datasets: low POSTN expression, *n* = 149; high POSTN expression, *n* = 150. **F**, Immunofluorescence staining of POSTN (green) and the GSC marker CD133 (red) in human GBM tissue. Scale bars, 50 μm (left) and 10 μm (right). **G** and **H**, Immunoblot analysis of POSTN in lysates of GSCs. POSTN protein levels were quantified (**H**). **I**, Immunoblot analysis of POSTN in SHG142 GSCs and SHG143 GSCs expressing control, shRNA control (sh-Control) or sh-POSTN. **J**, ELISA for the detection of secreted POSTN in the culture supernatant of GSCs expressing control, sh-Control or sh-POSTN. *n* = 3 biological replicates. **K**, Representative images of tumorspheres formed by SHG142 GSCs and SHG143 GSCs treated with control, sh-POSTN, or sh-POSTN + rhPOSTN (1 µg ml^− 1^, 48 h). Scale bar, 200 μm. **L**, Quantification of the number and diameter of SHG142 GSC- and SHG143 GSC-derived tumorspheres treated with control, sh-POSTN, or sh-POSTN + rhPOSTN (1 µg ml^− 1^, 48 h). *n* = 6 biological replicates. **M**, CCK-8 assay of SHG142 GSCs and SHG143 GSCs treated with control, sh-POSTN, or sh-POSTN + rhPOSTN (1 µg ml^− 1^, 48 h). *n* = 6 biological replicates. **N**, Representative images of immunofluorescence staining for CD133 and Nestin in SHG142 GSCs and SHG143 GSC-derived tumorspheres treated with control, sh-POSTN, or sh-POSTN + rhPOSTN (1 µg ml^− 1^, 48 h). Scale bar, 50 μm. **O** and **P**, Immunoblot analysis of CD133, Nestin and SOX2 in SHG142 GSCs and SHG143 GSCs treated with control, sh-POSTN, or sh-POSTN + rhPOSTN (1 µg ml^− 1^, 48 h). The error bars indicate the means ± SDs (**B**,** D**,** H**,** J**,** L**, and **P**). Two-tailed Student’s t test (**B**,** D**,** H**,** J**,** L**, and **P**). #, nonsignificant; *p < 0.05, **p < 0.01, ***p < 0.001, and ****p < 0.0001

To assess the potential clinical significance of POSTN, we examined the expression of POSTN in normal brain and glioma tissues through immunohistochemistry, quantitative real-time PCR (qRT‒PCR) and immunoblotting. POSTN expression was significantly increased (*p* < 0.0001) in glioma tissues compared with normal brain tissues and was highest in GBM among the glioma types (Fig. 1A-D). In addition, survival analysis of datasets from The Cancer Genome Atlas (TCGA), the Chinese Glioma Genome Atlas (CGGA) and Rembrandt collection revealed that high POSTN expression was associated with a worse prognosis than low POSTN expression in glioma patients (*p* < 0.0001) (Fig. 1E). Next, we performed single-cell RNA-seq analysis on 2 adult IDH1 wild-type GBM tissues obtained during surgery. We independently validated the cell type classification by using uniform manifold approximation and projection (UMAP) to quantify and map the expression of well-established lineage markers (Supplementary Fig. S1A). We confirmed that POSTN is expressed almost exclusively in tumor cells (Supplementary Fig. S1A and S1B). Tumor clusters were then isolated and the clustering procedure was repeated to differentiate more specific subtypes for tumor cells(Supplementary Fig. S1C and S1D). We further analyzed public single-cell datasets (GSE139448) of 3 adult glioblastomas and obtained similar results (Supplementary Fig. S1E-H). To further explore the potential link between POSTN expression and glioblastoma growth, we examined POSTN expression in human glioblastoma tissue via immunofluorescence and found that POSTN is preferentially expressed by cancer cells expressing a GSC marker (CD133) and is distributed in the area around GSCs (Fig. 1F). These data demonstrate that POSTN is preferentially secreted by GSCs in human glioblastoma and is associated with the development and progression of glioblastoma.

To investigate the impact of POSTN expression on GSC self-renewal, we cultured 6 GSC lines and selected the GSC lines SHG142 and SHG143, which presented the highest POSTN expression, and the GSC lines SHG141 and SHG144, which presented the lowest POSTN expression, for subsequent experiments (Fig. 1G and H). We first established stable POSTN-knockdown SHG142 GSCs and SHG143 GSCs via lentiviral transduction of shRNAs (Fig. 1I) and found that POSTN knockdown reduced POSTN secretion in GSCs (Fig. 1J). ShRNA-mediated depletion of POSTN significantly reduced the self-renewal ability of GSCs and that treatment with recombinant human POSTN (rhPOSTN) (1 µg ml^− 1^) for 48 h reversed the decrease in the self-renewal ability of GSCs caused by POSTN knockdown (Fig. 1k and l). We further investigated the impact of POSTN on GSC proliferation and the expression of stem cell markers through Cell Counting Kit-8 (CCK-8) assays, immunofluorescence staining, and immunoblot analysis. Knockdown of POSTN significantly reduced the proliferation ability and expression of the classical stemness biomarkers CD133, Nestin, and SOX2 in GSCs, and these effects were reversed by rhPOSTN treatment (1 µg ml-1, 48 h) (Fig. 1M-P).

Given that the knockdown of POSTN reduced the self-renewal ability of GSCs, we next investigated whether an increase in POSTN expression could promote GSC self-renewal. First, we established SHG141 GSCs and SHG144 GSCs with stable POSTN overexpression (Supplementary Fig. S1I) and confirmed that POSTN overexpression increased POSTN secretion in GSCs (Supplementary Fig. S1J). GSC tumorsphere formation assays revealed that both ectopic POSTN expression and rhPOSTN treatment (1 µg ml^− 1^, 48 h) increased GSC self-renewal (Supplementary Fig. S1K and S1L). POSTN overexpression increased the proliferative capacity of GSCs and the expression of CD133, Nestin and SOX2, and similar results were observed with rhPOSTN treatment (1 µg ml^− 1^, 48 h) (Supplementary Fig. S1M-P). Taken together, these findings demonstrate that POSTN is overexpressed in GBM and is a major promoter of GSC self-renewal.

### FOSL1 is a crucial transcription factor that mediates the promotion effect of the POSTN protein on GSC Self-renewal

**Fig. 2:**
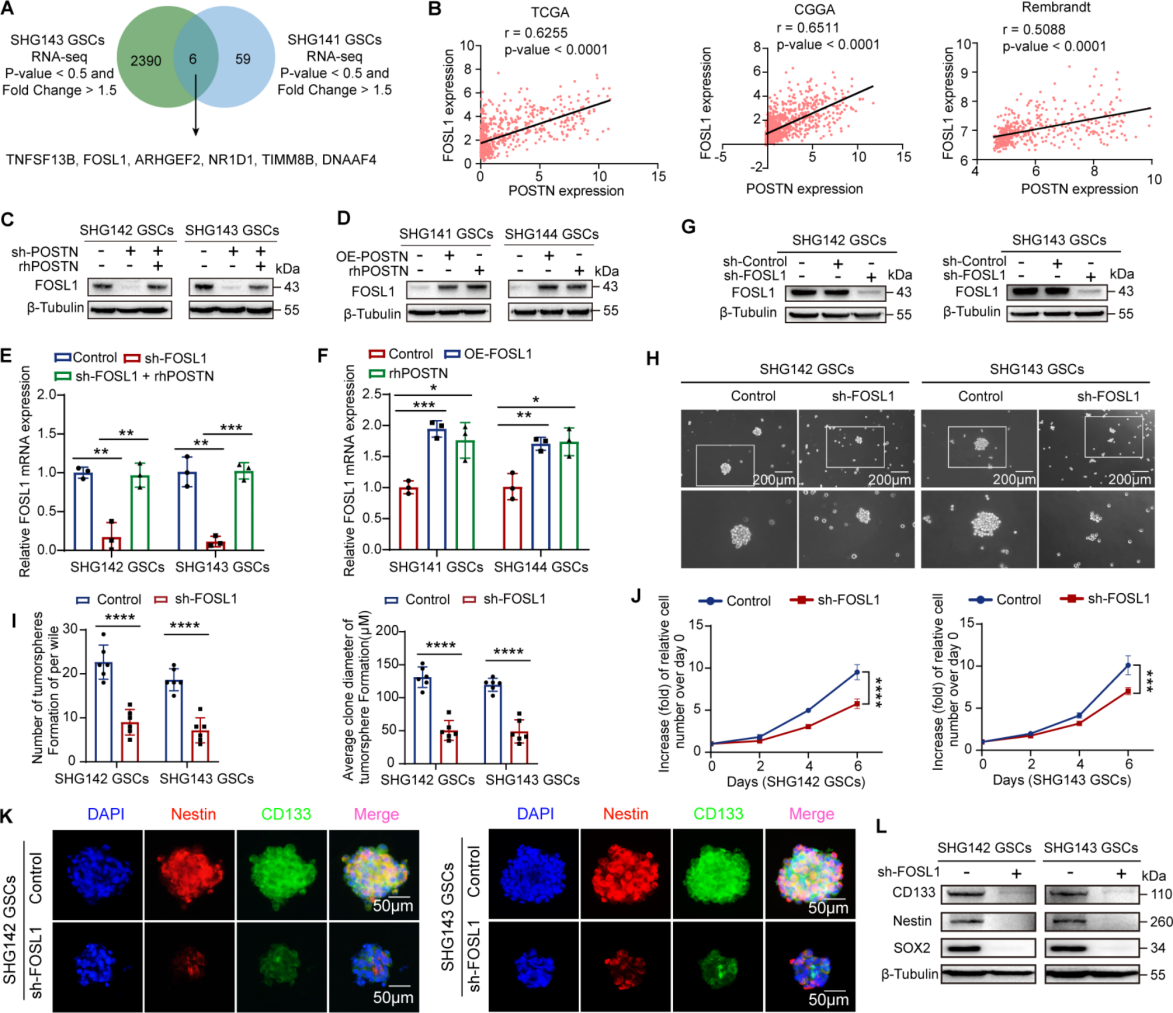
FOSL1 is a crucial protein that mediates the ability of POSTN to promote GSC self-renewal. **A**, Venn diagram showing the overlap of RNA-seq data for SHG143 GSCs and SHG141 GSCs. **B**, Correlations between POSTN and FOSL1 expression in the TCGA, CGGA and Rembrandt datasets. TCGA datasets: *n* = 675. CGGA datasets: *n* = 1018. Rembrandt datasets: *n* = 475. R and P values were determined via Pearson correlation analysis. **C**, Immunoblot analysis of FOSL1 in SHG142 GSCs and SHG143 GSCs treated with sh-POSTN or sh-POSTN + rhPOSTN (1 µg ml^− 1^, 48 h). **D**, Immunoblot analysis of FOSL1 in SHG141 GSCs and SHG144 GSCs treated with OE-POSTN or rhPOSTN (1 µg ml^− 1^, 48 h). **E**, qRT‒PCR analysis of POSTN mRNA expression in SHG142 GSCs and SHG143 GSCs treated with sh-POSTN or sh-POSTN + rhPOSTN (1 µg ml^− 1^, 48 h). **F**, qRT‒PCR analysis of POSTN mRNA expression in SHG141 GSCs and SHG144 GSCs treated with OE-POSTN or rhPOSTN (1 µg ml^− 1^, 48 h). **G**, Immunoblot analysis of FOSL1 in SHG142 GSCs and SHG143 GSCs expressing sh-Control or sh-FOSL1. **H**, Representative images of tumorspheres formed by SHG142 GSCs and SHG143 GSCs expressing control or sh-FOSL1. Scale bar, 200 μm. **I**, Quantification of the number and diameter of SHG142 GSC- and SHG143 GSC-derived tumorspheres expressing control or sh-FOSL1; *n* = 6 biological replicates. **J**, CCK-8 assay of SHG142 GSCs and SHG143 GSCs expressing control or sh-FOSL1; *n* = 6 biological replicates. **K**, Representative images of immunofluorescence staining of CD133 and Nestin in SHG142 GSCs and SHG143 GSC-derived tumorspheres expressing control or sh-FOSL1. Scale bar, 50 μm. **L**, Immunoblot analysis of CD133, Nestin and SOX2 in SHG142 GSCs and SHG143 GSCs expressing control or sh-FOSL1. The error bars indicate the means ± SDs (**E**,** F**, and **I**). Two-tailed Student’s t test (**E**,** F**,** I**, and **J**). *p < 0.05, **p < 0.01, ***p < 0.001, and ****p < 0.0001

To determine the molecular mechanisms underlying the effect of POSTN on GSC self-renewal, we performed RNA sequencing (RNA-seq) analysis of SHG143 GSCs with or without POSTN knockdown and SHG141 GSCs with or without rhPOSTN treatment. We then determined the overlap between the differentially expressed genes in the two GSC lines and identified 6 overlapping genes (Fig. 2A). TCGA data analysis revealed that the expression of POSTN had the strongest correlation with the expression of FOSL1 among these six genes and that this correlation was significantly positive; this correlation was confirmed by analysis of datasets from CGGA and Rembrandt (Fig. 2B; Supplementary Fig. S2A). Analysis of protein and mRNA expression by immunoblotting and qRT‒PCR revealed that FOSL1 was a critical downstream target of POSTN (Fig. 2C‒F). The FOSL1 gene encodes the transcription factor FOS-like antigen 1 (FOSL1), which is a component of the AP1 complex. FOSL1 is upregulated in numerous malignancies, including GBM, and is implicated in cancer occurrence and development [27]. Previous studies have reported that FOSL1 is an essential transcriptional regulator of the proneural-to-mesenchymal transition in GSCs [28]. FOSL1 reprograms differentiated cancer cells into stem-like cells by regulating four stemness-related transcription factors [29]. Thus, FOSL1 may be correlated with the self-renewal of GSCs.

To further assess the expression of FOSL1 in glioma and the association between FOSL1 expression and prognosis in glioma patients, we examined the expression of POSTN in normal brain and glioma tissues and analyzed data from the TCGA, CGGA and Rembrandt databases. Immunohistochemical staining of a tissue microarray revealed that FOSL1 expression was significantly elevated in glioma tissues compared with normal brain tissues, with the highest expression in GBM tissues (Supplementary Fig. S2B). In addition, survival analysis revealed that high expression of FOSL1 was associated with poor survival in glioma patients (Supplementary Fig. S2C).

We next examined the effects of FOSL1 on the self-renewal and proliferation of GSCs. We generated GSC lines with stable FOSL1 knockdown or overexpression (Fig. 2G; Supplementary Fig. S2D and S2E). Tumorsphere formation and CCK-8 assays revealed that the knockdown of FOSL1 in GSCs significantly reduced their self-renewal and proliferation capacities (Fig. 2H-J), whereas the overexpression of FOSL1 in GSCs increased their self-renewal and proliferation capacities (Supplementary Fig. S2F-H). Immunofluorescence staining and immunoblotting demonstrated that the knockdown of FOSL1 significantly reduced the expression of the stemness markers CD133, Nestin and SOX2 (Fig. 2K and L). In contrast, the overexpression of FOSL1 increased the expression of stemness markers (Supplementary Fig. S2I, J). Furthermore, the knockdown of FOSL1 abrogated the effects of the increase in cellular POSTN expression on self-renewal (Supplementary Fig. S3A and S3B), proliferation (Supplementary Fig. S3C) and stemness marker expression (Supplementary Fig. S3D-F) in GSCs. In summary, FOSL1 plays an important role in the ability of POSTN to promote the self-renewal and proliferation of GSCs.

### FOSL1 is regulated by β-Catenin in the POSTN/αvβ3/PI3K/AKT pathway

**Fig. 3:**
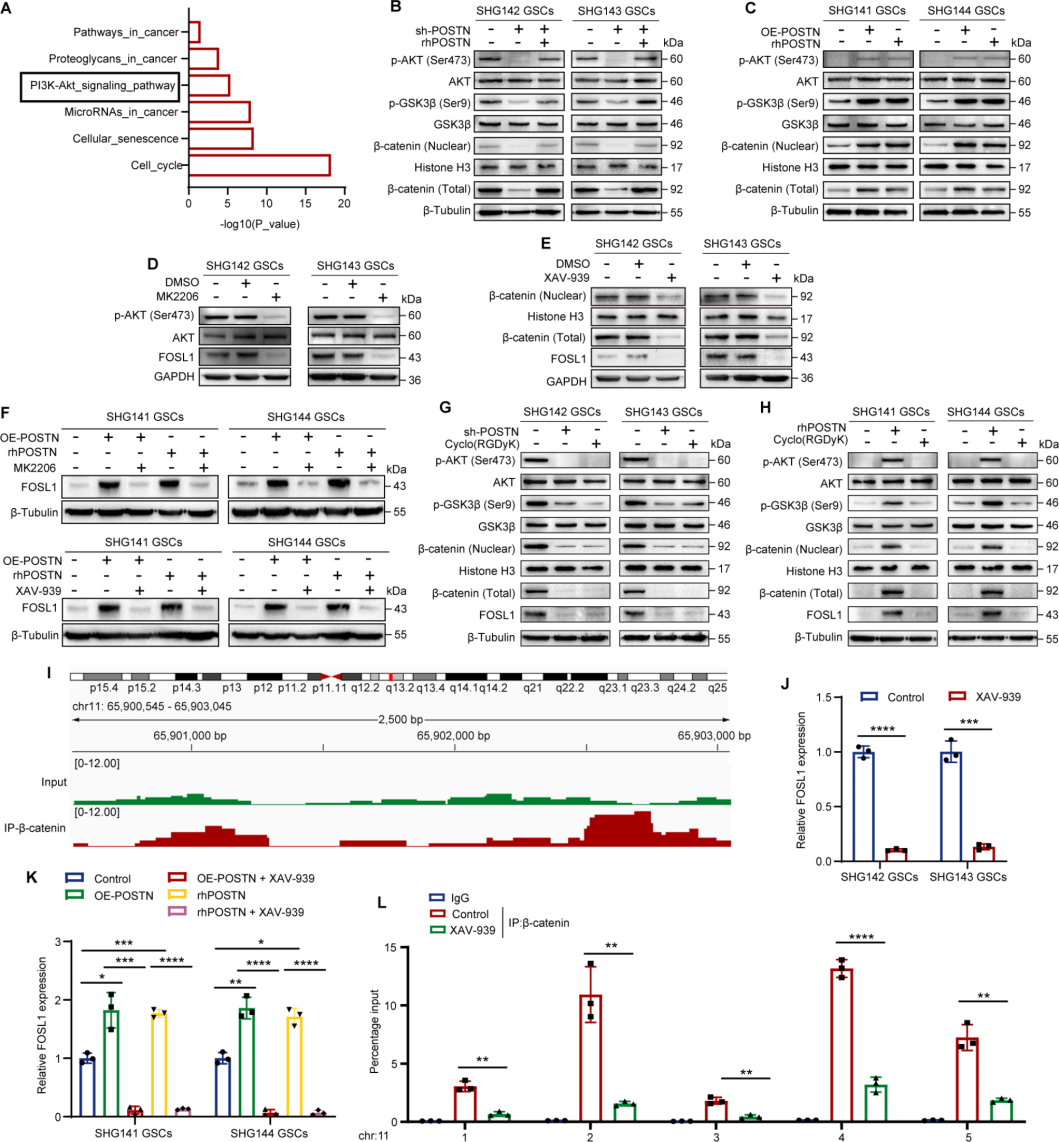
FOSL1 is regulated by β-catenin in the POSTN/αvβ3/PI3K/AKT pathway. **A**, KEGG analysis results showing the six oncogenic pathways affected by POSTN depletion. **B**, Immunoblot analysis of the PI3K/AKT/β-catenin pathway in SHG142 GSCs and SHG143 GSCs treated with sh-POSTN or sh-POSTN + rhPOSTN (1 µg ml^− 1^, 48 h). **C**, Immunoblot analysis of the PI3K/AKT/β-catenin pathway in SHG141 GSCs and SHG144 GSCs treated with OE-POSTN or rhPOSTN (1 µg ml^− 1^, 48 h). **D**, Immunoblot analysis of р-AKT (Ser473), AKT and FOSL1 in SHG142 GSCs and SHG143 GSCs treated with MK2206 (10 µM, 48 h). **E**, Immunoblot analysis of β-catenin (total) and FOSL1 in SHG142 GSCs and SHG143 GSCs treated with XAV-939 (5 µM, 48 h). **F**, Immunoblot analysis of FOSL1 in SHG141 GSCs and SHG144 GSCs treated with control, OE-POSTN, OE-POSTN + MK2206 (10 µM)/XAV-939 (5 µM), rhPOSTN (1 µg ml^− 1^), or rhPOSTN (1 µg ml^− 1^) + MK2206 (10 µM)/XAV-939 (5 µM) for 48 h. **G** and **H**, Immunoblot analysis of the PI3K/AKT/β-catenin/FOSL1 pathway in SHG142 GSCs, SHG143 GSCs, SHG141 GSCs and SHG144 GSCs treated with sh-POSTN, rhPOSTN (1 µg ml^− 1^, 48 h) or cyclo(RGDyK) (100 nM, 48 h). **I**, ChIP-seq data showing that β-catenin is enriched in the FOSL1 promoter in SHG143 GSCs. **J**, qRT‒PCR analysis of FOSL1 mRNA expression in SHG142 GSCs and SHG143 GSCs treated with the control or XAV-939 (5 µM, 48 h). **K**, qRT‒PCR analysis of FOSL1 mRNA expression in SHG141 GSCs and SHG144 GSCs treated with control, OE-POSTN, OE-POSTN + XAV-939 (5 µM), rhPOSTN (1 µg ml^− 1^), or rhPOSTN (1 µg ml^− 1^) + XAV-939 (5 µM) for 48 h. **L**, ChIP‒qPCR analysis of β-catenin occupancy at the FOSL1 promoter. IgG was used as a reference. The error bars indicate the means ± SDs (**J**,** K**, and **L**). Two-tailed Student’s t test (**J**,** K**, and **L**). *p < 0.05, **p < 0.01, ***p < 0.001, and ****p < 0.0001

To determine the mechanism by which POSTN regulates GSC self-renewal, we performed Kyoto Encyclopedia of Genes and Genomes (KEGG) pathway enrichment analysis of the differentially expressed genes in SHG143 GSCs identified via RNA-seq (Fig. 3A). This analysis revealed that the POSTN-related genes were associated with multiple cancer-related signaling pathways, including the PI3K/AKT signaling pathway. Previous studies have reported that POSTN can bind to integrin αVβ3 and activate the PI3K/Akt pathway in various types of cells [16, 17]. β-Catenin regulates the transcription of FOSL1 [30]. We thus hypothesized that POSTN may promote FOSL1 transcription through the PI3K/AKT/GSK3β/β-catenin signaling pathway. Immunoblotting revealed that the levels of phospho-AKT (p-AKT) (Ser473), phospho-GSK3β (p-GSK3β) (Ser9) and β-catenin were decreased after POSTN knockdown in SHG142 GSCs and SHG143 GSCs and that rhPOSTN treatment restored the levels of p-AKT (Ser473), p-GSK3β (Ser9) and β-catenin (Fig. 3B). Correspondingly, overexpression of POSTN promoted increases in p-AKT, p-GSK3β and β-catenin levels (Fig. 3C). To determine whether FOSL1 is regulated by AKT and β-catenin, we used MK-2206 (10 µM, 48 h) to inhibit AKT phosphorylation and XAV-939 (5 µM, 48 h) to inhibit β-catenin expression (Fig. 3D and E). Immunoblotting analysis revealed that MK-2206 and XAV-939 suppressed the protein expression of FOSL1 and reversed the FOSL1 upregulation induced by POSTN overexpression or rhPOSTN treatment (Fig. 3F).

Previous studies have shown that integrin αvβ3 is a major receptor for POSTN in the activation of PI3K signaling [16]. We next investigated the impact of the selective integrin αVβ3 inhibitor cyclo(RGDyK) on POSTN-mediated activation of the AKT/β-catenin/FOSL1 pathway. After GSCs were treated with 100 nM cyclo(RGDyK) for 48 h, the levels of p-AKT (Ser473), p-GSK3β (Ser9), β-catenin and FOSL1 were reduced, which was consistent with the findings obtained upon POSTN knockdown (Fig. 3G). Moreover, disruption of integrin signaling by cyclo(RGDyK) treatment in SHG141 GSCs and SHG144 GSCs significantly suppressed the activation of the AKT/β-catenin/FOSL1 signaling pathway induced by rhPOSTN (Fig. 3H). These observations suggest that FOSL1 expression is induced via the POSTN/αvβ3/PI3K/AKT/β-catenin axis.

To further investigate whether β-catenin directly transcriptionally regulates FOSL1 expression, we performed ChIP-seq experiments. We found that β-catenin can bind to the FOSL1 promoter region in SHG143 GSCs (Fig. 3I). Next, we confirmed that β-catenin inhibition resulted in a significant reduction in the FOSL1 mRNA level (Fig. 3J and K). ChIP‒PCR was then performed using IgG and an anti-β-catenin antibody and five pairs of FOSL1 promoter-specific genomic PCR primers. Compared with that in control cells, the enrichment of β-catenin at the FOSL1 promoter region in XAV-939-treated SHG143 GSCs was abolished (Fig. 3L). These findings suggest that FOSL1 transcription is regulated by β-catenin downstream of the POSTN/αvβ3/PI3K/AKT pathway.

### POSTN depletion decreases the tumorigenicity of GSCs by reducing FOSL1 expression

**Fig. 4:**
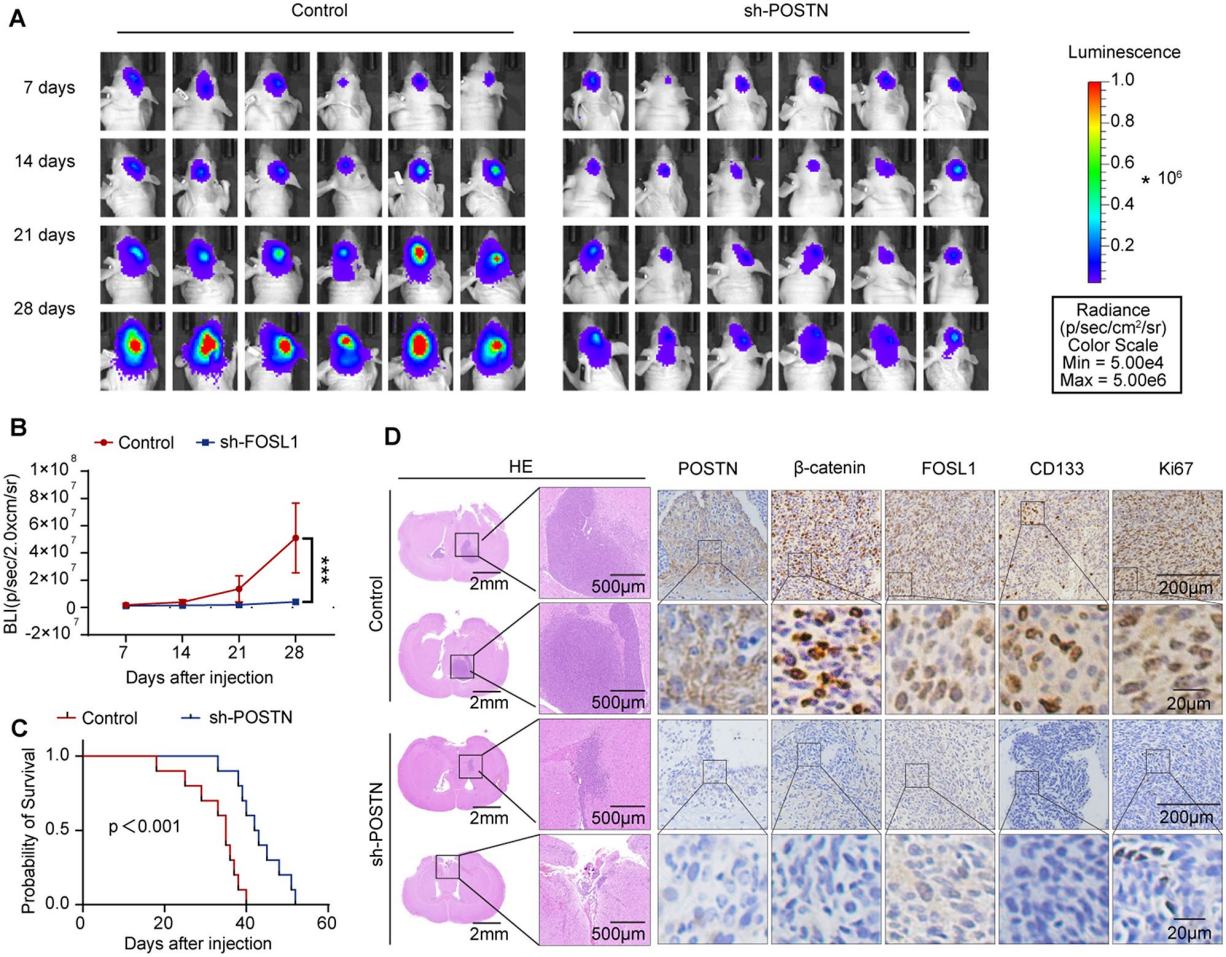
POSTN depletion inhibits the tumorigenicity of GSCs by reducing FOSL1 expression. **A** and **B**, In vivo bioluminescence images (**A**) and quantitative analysis (**B**) of xenografts derived from luciferase-labeled SHG143 GSCs expressing control or sh-POSTN on the indicated days after implantation; *n* = 8 mice. The data are presented as the means ± SDs. Two-tailed Student’s t test. ***p < 0.001. **C**, Kaplan–Meier survival curves of mice implanted with SHG143 GSCs expressing control or sh-POSTN; *n* = 10 mice. Log–rank test. **D**, Representative images of HE staining and IHC staining for POSTN, β-catenin, FOSL1, CD133, and Ki67 in sections of SHG143 GSC xenografts from each group. Scale bars: 2 mm for HE staining (left), 500 μm for HE staining (right), 200 μm for IHC staining (top) and 200 μm for IHC staining (bottom)

To assess the effect of POSTN on GBM growth in vivo, we stably transduced SHG143 GSCs with lentiviral vectors containing sh-POSTN or empty vector and then separately implanted these two groups of cells into the brains of immunocompromised mice. Bioluminescence imaging on days 7, 14, 21, and 28 revealed that POSTN depletion significantly inhibited tumor growth (Fig. 4A and B) and prolonged the survival of tumor-bearing mice (Fig. 4C). HE staining and immunohistochemical analysis revealed that POSTN knockdown reduced tumor growth and the expression of β-catenin and FOSL1, the GSC marker CD133, and the proliferation marker Ki67 (Fig. 4D). Collectively, these results validate the crucial function of POSTN in GSC tumorigenicity.

### POSTN increases CD70 expression in microglia, thereby promoting treg development and function

**Fig. 5:**
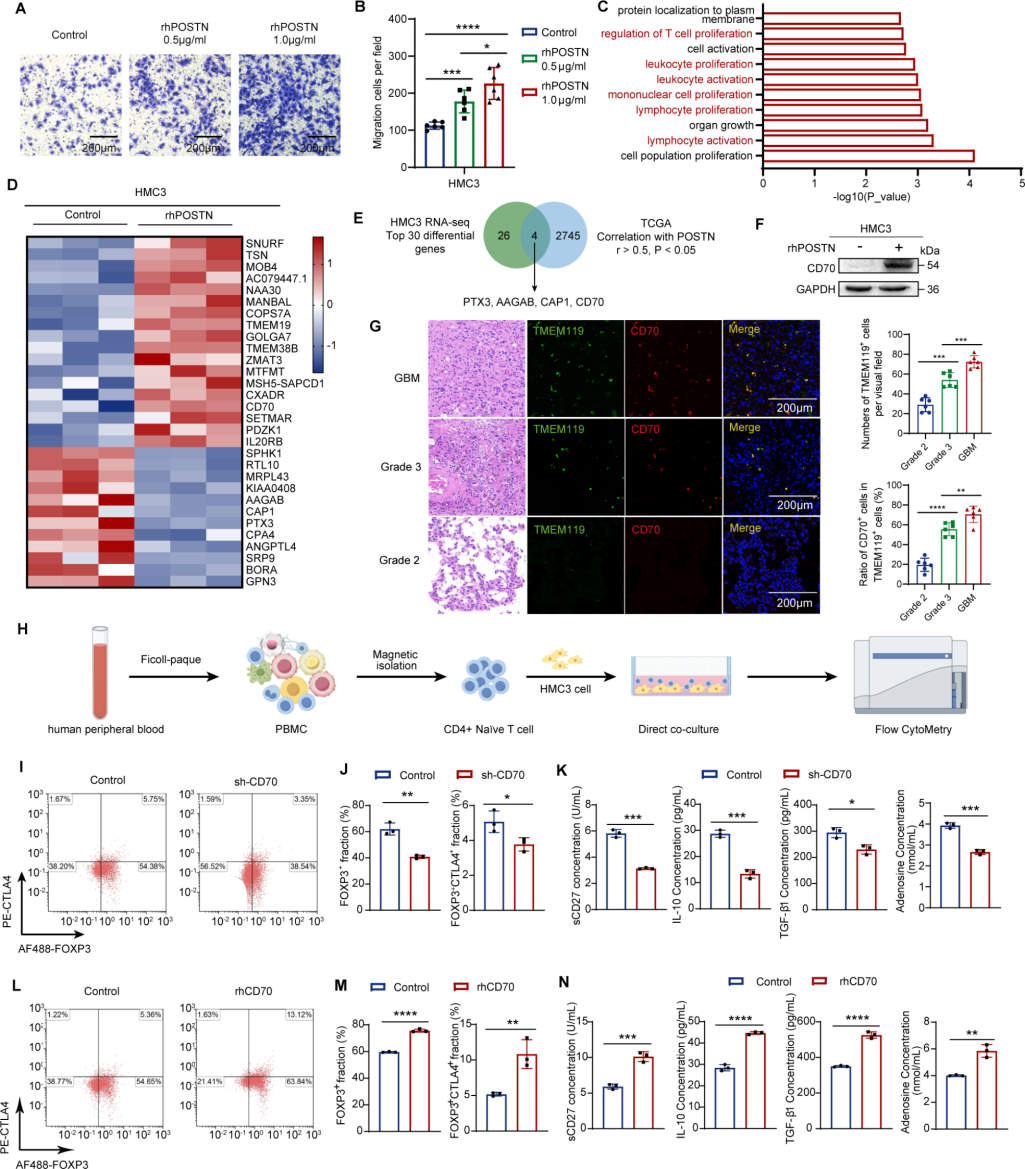
POSTN increases CD70 expression in microglia, thereby promoting Treg development and function. **A** and **B**, Representative images (**A**) and quantitative analysis (**B**) of the relative migration of HMC3 cells following stimulation with rhPOSTN; *n* = 6. Scale bars, 200 μm. **C**, Transcriptomic profiling of HMC3 cells following rhPOSTN (1 µg ml^− 1^) treatment for 48 h and Gene Ontology biological process analysis results showing the ten pathways affected by rhPOSTN treatment. **D**, RNA-seq analysis of the top 30 genes whose expression was differentially regulated in HMC3 cells after treatment with the control or rhPOSTN (1 µg ml^− 1^, 48 h). **E**, Venn diagram showing the overlap of HMC3 cell RNA-seq data and genes correlated with POSTN (correlation coefficient greater than 0.5) in the TCGA database. **F**, Immunoblots showing the levels of CD70 in HMC3 cells treated with the control or rhPOSTN (1 µg ml^− 1^, 48 h). **G**, Left: Representative images of HE staining and immunofluorescence staining of TMEM119 and CD70 in glioma samples. Scale bars, 200 μm. Right: The number of TMEM119^+^ cells in each field of view and the proportion of CD70^+^ cells among TMEM119^+^ cells. Grade 2: *n* = 6; Grade 3: *n* = 6; GBM: *n* = 6. **H**, Images of the in vitro direct coculture systems of HMC3 cells and naïve CD4^+^ T cells. **I** and **J**, Proportions of total FOXP3^+^ Tregs and FOXP3^+^CTLA4^+^ activated Tregs in direct coculture systems with HMC3 cells expressing control or sh-CD70. **J**, *n* = 3. **K**, Changes in the levels of sCD27 and the immunosuppressive factors IL-10, TGF-β1, and adenosine in the direct coculture system. sCD27, IL-10 and TGF-β1 were measured via ELISA, and adenosine was measured via a fluorescent adenosine assay; *n* = 3. **L** and **M**, Proportions of total FOXP3^+^ Tregs and FOXP3^+^CTLA4^+^ activated Tregs in direct coculture systems with HMC3 cells treated with control or rhPOSTN (1 µg ml^− 1^) for 48 h. **M**, *n* = 3. **N**, Changes in the levels of sCD27 and the immunosuppressive factors IL-10, TGF-β1, and adenosine in the direct coculture system. sCD27, IL-10 and TGF-β1 were measured via ELISA, and adenosine was measured via a fluorescent adenosine assay; *n* = 3. The error bars indicate the means ± SDs (**B**,** J**,** K**,** M** and **N**). Two-tailed Student’s t test (**B**,** J**,** K**,** M** and **N**). *p < 0.05, **p < 0.01 and ***p < 0.001

Previous studies have demonstrated that high stemness of GBM cells is positively correlated with increased activity of immunosuppressive pathways [31]. CSCs recruit and regulate microglia by secreting factors such as LGMN, sCSF-1, MIC-1, and WISP1, leading to the establishment of an immunosuppressive TME [14, 15, 32]. Since studies have indicated that POSTN secreted by GSCs recruits M2 TAMs and promotes the malignant growth of GBM [22], we hypothesized that POSTN might also recruit microglia to the tumor microenvironment. Transwell migration assays revealed that the migration of human microglial HMC3 cells increased significantly with increasing rhPOSTN concentration (Fig. 5A and B).

To further confirm whether POSTN modulates the immunophenotype of microglia, we performed RNA-seq analysis on microglia treated with or without rhPOSTN (Supplementary Fig. S4A). Gene Ontology biological process enrichment analysis of the differentially expressed genes revealed that six of the top 10 enriched biological processes were related to the proliferation and activation of immune cells (Fig. 5C), confirming that POSTN can regulate the immunophenotype of microglia. To further explore the role of microglia with immunophenotypic regulation by the POSTN protein in the immune microenvironment of GBM, we identified the 30 genes with the greatest changes in expression, as determined by RNA-seq analysis of HMC3 cells, and found that the expression levels of 4 of these genes were related to those of POSTN in the TCGA database (Fig. 5D and E, Supplementary Table S3). Among the four genes, only CD70 was related to the proliferation and activation of immune cells according to Gene Ontology biological process enrichment analysis of the differentially expressed genes. Analysis of the TCGA and CGGA data suggested that CD70 expression was significantly positively correlated with POSTN expression in GBM (Supplementary Fig. S4B). Immunoblot analysis revealed that the CD70 level in microglia was significantly increased after rhPOSTN treatment (Fig. 5F), suggesting that POSTN regulates the microglial immunophenotype through CD70.

CD70–CD27 signaling generates a costimulatory signal, and CD70–CD27 interactions facilitate cancer cell immune evasion and cancer progression by promoting Treg development and immunosuppressive activity [33, 34]. However, the function of CD70 in GBM remains unclear. To obtain insight into the function of CD70 in glioma, we analyzed the relationship between CD70 expression and patient prognosis. High CD70 expression was associated with poor prognosis in glioma patients (Supplementary Fig. S4C). Moreover, immunofluorescence staining of CD70 and the microglial marker TMEM119 in a tissue microarray revealed significantly increased microglial recruitment, accompanied by high expression of CD70, in GBM tissues (Fig. 5G). We further investigated the correlation between CD70 expression and Treg infiltration in glioma and found that CD70 expression was significantly positively correlated with Treg infiltration (Supplementary Fig. S4D). These results indicate that high expression of CD70 in microglia may be related to Treg infiltration.

Tregs account for 5–15% of CD4^+^ T cells and promote the immune tolerance of cancer cells. The immunosuppressive microenvironment of glioma induced by Tregs is an important reason for the poor prognosis of glioma patients [35]. Effector Tregs are the predominant subtype of Tregs in tumor tissues and are characterized by coexpression of the transcription factor FOXP3 and the transmembrane protein CTLA4 [36]. Effector Tregs induce defects in antitumor T-cell proliferation and cytotoxicity by producing various suppressive cytokines, such as TGF-β1, IL-10 and cyclic adenosine monophosphate, leading to dysregulation of antitumor T-cell immune homeostasis [33, 36]. To elucidate whether the expression of CD70 in microglia affects Treg development and activation, we first extracted mononuclear cells from the peripheral blood of healthy volunteers and further isolated naïve CD4^+^ T cells through magnetic bead sorting (Supplementary Fig. S5A). Following T-cell receptor (TCR) activation (Supplementary Fig. S5B) and the induction of T-cell differentiation (Supplementary Fig. S5C), flow cytometry revealed that approximately 35.45% of the cells were FOXP3^+^ and that approximately 3.27% were FOXP3^+^CTLA4^+^ (Supplementary Fig. S5D). We next established an in vitro coculture system to directly coculture naïve CD4^+^ T cells with CD70-knockdown (Supplementary Fig. S5E) or control HMC3 cells to determine the effect of CD70 on Treg development and function (Fig. 5H). For direct coculture of naïve CD4^+^ T cells with HMC3 cells, HMC3 cells were seeded in 24-well plates at a density of 5 × 10^4^ cells/well and cultured for 24 h until the HMC3 cells adhered to the plate. After HMC3 cells adhered to the plate, the supernatant in the 24-well plates was discarded, and 2 × 10^5^ naïve CD4 + T cells were seeded into 24-well plates for direct coculture with HMC3 cells. Compared with monoculture of naïve CD4^+^ T cells, direct coculture of naïve CD4^+^ T cells and HMC3 cells promoted the polarization of naïve CD4^+^ T cells to FOXP3^+^ Tregs and upregulated the expression of the important eTreg marker CTLA4 (Fig. 5I and J). CD70 knockdown in microglia decreased the expression of FOXP3 (*p* < 0.01) and CTLA4 (*p* < 0.05) in naïve CD4^+^ T cells through the CD70–CD27 axis (Fig. 5I and J) and inhibited the secretion of IL-10 (*p* < 0.001), TGF-β1 (*p* < 0.05), and adenosine (*p* < 0.001) in the coculture system (Fig. 5K). Correspondingly, treatment with recombinant human CD70 (rhCD70) protein promoted Treg development and activation and increased IL-10, TGF-β1, and adenosine secretion (Fig. 5L-N). Considering that microglia also produce IL-10, TGF-β1, and adenosine, we examined the effects of CD70 knockdown, rhCD70 treatment and rhPOSTN treatment on IL-10, TGF-β1, and adenosine secretion by microglia. The ELISA results confirmed that CD70 knockdown, rhCD70 treatment and rhPOSTN treatment did not affect the secretion of IL-10, TGF-β1 or adenosine by microglia (Supplementary Fig. S5F). CD70 is expressed in both tumor cells and microglia [33]. Our SHG141 GSC RNA-seq, SHG143 GSC RNA-seq and immunoblot analysis results revealed that neither POSTN knockdown nor rhPOSTN treatment significantly affected CD70 expression in GSCs (Supplementary Fig. S5G-I). Therefore, these results suggest that POSTN increases CD70 expression in microglia but not in GSCs, thereby promoting Treg development and function.

### CD70 is a key factor in POSTN promoting the development and function of Tregs through microglia

**Fig. 6:**
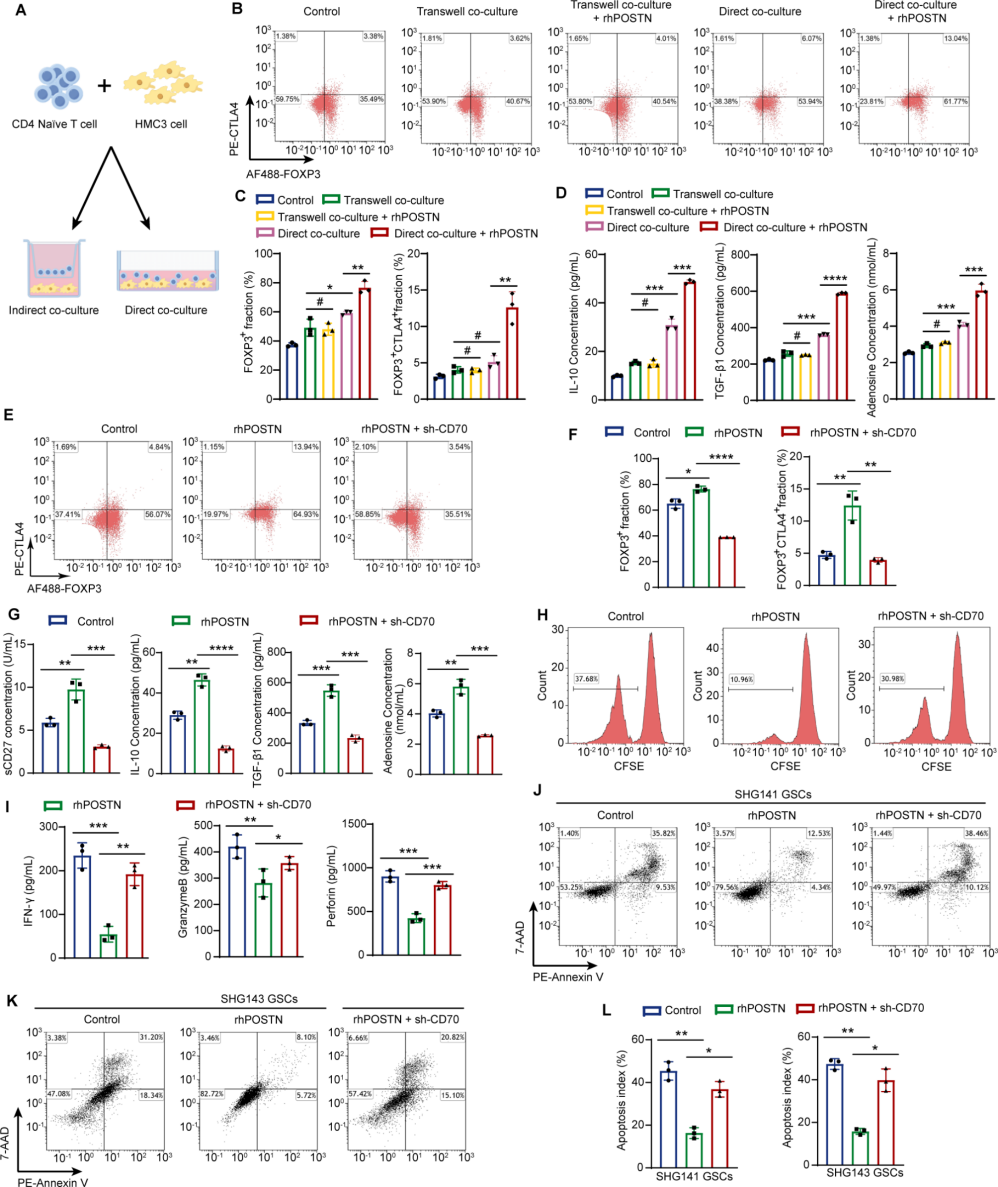
CD70 is a key factor through which POSTN promotes the development and function of Tregs through microglia. **A** and **B**, Images of the in vitro direct and indirect coculture systems of HMC3 cells and naïve CD4^+^ T cells. **B**, *n* = 3 **C**, Proportions of total FOXP3^+^ Tregs and activated FOXP3^+^CTLA4^+^ Tregs in the coculture systems. Before coculture, HMC3 cells were treated with or without rhPOSTN (1 µg ml^− 1^) for 48 h. **D**, Changes in the levels of the immunosuppressive factors IL-10, TGF-β1, and adenosine in the coculture systems. IL-10 and TGF-β1 levels were measured via ELISA, and adenosine levels were measured via a fluorescent adenosine assay; *n* = 3. **E** and **F**, Proportions of total FOXP3^+^ Tregs and FOXP3^+^CTLA4^+^ activated Tregs in the coculture system. Before coculture, HMC3 cells were treated with control, rhPOSTN (1 µg ml^− 1^), or rhPOSTN (1 µg ml^− 1^) + sh-CD70 for 48 h. F) *n* = 3. **G**, Changes in the levels of sCD27 and the immunosuppressive factors IL-10, TGF-β1, and adenosine in the direct coculture system. sCD27, IL-10 and TGF-β1 were measured via ELISA, and adenosine was measured via a fluorescent adenosine assay; *n* = 3. **H**, Flow cytometry plot showing CD8^+^ T-cell proliferation. **I**, IFN-γ, granzyme B and perforin levels in the cell supernatants were quantified via ELISA; *n* = 3. **J-L**, Apoptosis assay of SHG141 GSCs and SHG143 GSCs. **L**, *n* = 3. The error bars indicate the means ± SDs (**C**,** D**,** F**,** G**,** I**, and **L**). Two-tailed Student’s t test (**C**,** D**,** F**,** G**,** I**, and **L**). #, nonsignificant; *p < 0.05, **p < 0.01, ***p < 0.001, and ****p < 0.0001

To characterize the mechanism by which POSTN promotes Treg development and activation mediated by microglia, we established a coculture system in which HMC3 cells were directly or indirectly cocultured with naïve CD4^+^ T cells in vitro. (Fig. 6A). HMC3 cells were treated with or without rhPOSTN for 48 h before coculture. Compared with naïve CD4^+^ T cells cultured alone or indirectly cocultured with HMC3 cells, naïve CD4^+^ T cells directly cocultured with HMC3 cells presented significantly increased FOXP3 and CTLA4 expression and increased IL-10, TGF-β1, and adenosine secretion (Fig. 6B-D). Furthermore, treatment of HMC3 cells with rhPOSTN significantly promoted the activation of naïve CD4^+^ T cells and their differentiation into Tregs in the direct coculture system but not in the indirect coculture system (Fig. 6B-D). Afterward, we knocked down CD70 expression in HMC3 cells via lentiviral transduction (Supplementary Fig. S5E). CD70 knockdown significantly suppressed the activation of naïve CD4^+^ T cells and their differentiation into Tregs by blocking the CD70–CD27 axis and inhibiting IL-10, TGF-β1, and adenosine secretion (Fig. 6E-G). We mixed cocultured CD4^+^ T cells with CD8^+^ T cells isolated from the same donor at a ratio of 1:1 to perform a Treg suppression assay. The results confirmed that naïve CD4^+^ T cells significantly inhibited the proliferation of CD8^+^ T cells after coculture with rhPOSTN-treated HMC3 cells; CD70 knockdown reversed this inhibitory effect (Fig. 6H).

To assess the influence of Tregs on the tumoricidal activities of CD8^+^ T cells, we first seeded normal or CD70-knockdown HMC3 cells in 24-well plates at a density of 5 × 10^4^ cells/well and cultured them for 24 h until the HMC3 cells adhered to the plate. After HMC3 cells adhered to the plate, they were treated with or without rhPOSTN (1 µg ml^− 1^) for 48 h. The supernatant in the 24-well plate was then discarded, and 2 × 10^5^ naïve CD4^+^ T cells were seeded into 24-well plates for direct coculture with HMC3 cells for 3 days. After 3 days of coculture, the cocultured CD4^+^ T cells and CFSE-stained CD8^+^ T cells were mixed and cultured at a ratio of 1:1 for 48 h. Our results revealed that naïve CD4^+^ T cells significantly inhibited the secretion of IFN-γ, granzyme B and perforin by CD8^+^ T cells after coculture with rhPOSTN-treated HMC3 cells (Fig. 6I). Knockdown of CD70 in microglia inhibited Treg development and function, thereby increasing the secretion of IFN-γ and granzyme B. In addition, we added GSCs to the direct coculture system of CD4^+^ T cells and CD8^+^ T cells (the ratio of CD4^+^ T cells, CD8^+^ T cells and GSCs was 1:1:2). CD4^+^ T cells significantly inhibited the ability of CD8^+^ T cells to kill GSCs after coculture with rhPOSTN-treated HMC3 cells, and the knockdown of CD70 in microglia inhibited Treg development and function, thereby promoting the cytotoxic effect of CD8^+^ T cells on GSCs (Fig. 6J-L). Taken together, these findings demonstrate that CD70 in microglia is a key factor through which POSTN promotes Treg development and function.

### CD70 is regulated by NFκB via the POSTN/αvβ3/PI3K/AKT pathway

**Fig. 7:**
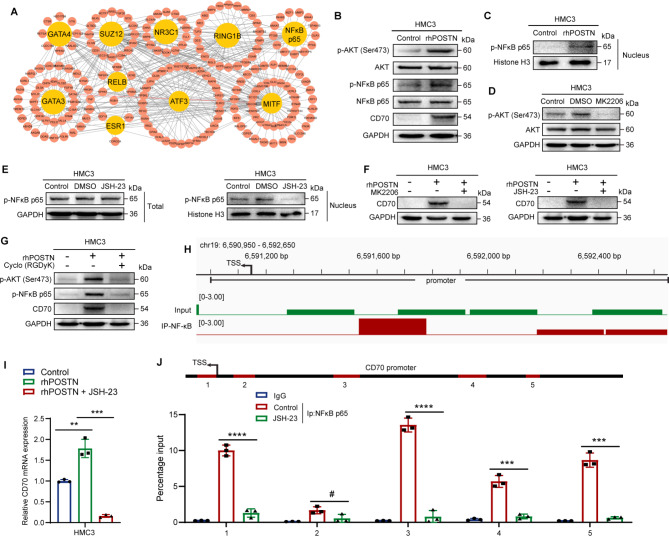
CD70 is regulated by NFκB in the POSTN/αvβ3/PI3K/AKT pathway. **A**, The correlations of differentially expressed genes in HMC3 cells after rhPOSTN (1 µg ml^− 1^) treatment for 48 h were analyzed via ChIP-seq enrichment analysis via the ChEA database in Enrichr, resulting in the identification of 10 transcription factors and their possible targets. **B**, Immunoblots showing p-AKT (Ser473), AKT, p-NFκB p65, NFκB p65 and CD70 levels in HMC3 cells treated with or without rhPOSTN (1 µg ml^− 1^) for 48 h. **C**, The nuclear lysate was used to determine the p-NFκB p65 level in HMC3 cells treated with or without rhPOSTN (1 µg ml^− 1^) for 48 h. **D**, Immunoblot analysis of р-AKT (Ser473) and AKT in HMC3 cells treated with the control, DMSO or MK2206 (10 µM) for 48 h. **E**, The total lysate and the nuclear fraction were used to determine the p-NFκB p65 level in HMC3 cells after treatment with JSH-23 (10 µM) for 48 h. **F**, Immunoblots showing CD70 expression in HMC3 cells treated with control, rhPOSTN (1 µg ml^− 1^) or rhPOSTN (1 µg ml^− 1^) + MK2206 (10 µM)/JSH-23 (10 µM) for 48 h. **G**, Immunoblot analysis of p-AKT (Ser473), p-NFκB p65, and CD70 in HMC3 cells treated with control, rhPOSTN (1 µg ml^− 1^) or rhPOSTN (1 µg ml^− 1^) + Cyclo(RGDyK) (100 nM) for 48 h. **H**, ChIP-seq data showing that p-NFκB p65 was enriched in the CD70 promoter in HMC3 cells. **I**, qRT‒PCR analysis of CD70 mRNA expression in HMC3 cells treated with control, rhPOSTN (1 µg ml^− 1^) or rhPOSTN (1 µg ml^− 1^) + Cyclo(RGDyK) (100 nM) for 48 h. **J**, ChIP‒qPCR analysis of p-NFκB p65 occupancy at the promoter of CD70. IgG was used as a reference. The error bars indicate the means ± SDs (**I**, and **J**). Two-tailed Student’s t test (**I**, and **J**). #, nonsignificant; **p < 0.01, ***p < 0.001, and ****p < 0.0001

To determine the molecular mechanism by which POSTN regulates CD70 expression in glioma, we performed chromatin immunoprecipitation enrichment analysis (ChEA) [37] of RNA-seq data from HMC3 cells to identify transcription factors that potentially mediate transcriptional changes in microglia. Through ChEA, we identified the top 10 transcription factors that may mediate POSTN-regulated microglial transcriptional changes, among which NFκB p65, SUZ12 and MITF might regulate CD70 transcription (Fig. 7A). qRT‒PCR and immunoblot analyses confirmed that the knockdown of NFκB p65 alone significantly inhibited the expression of CD70 (Supplementary Fig. S6A and S6B). Previous experiments have shown that POSTN activates the NF-κB pathway in different cells, but the mechanism by which POSTN activates the NFκB pathway is unclear [38]. Previous reports confirmed that NFKB can be activated by p-AKT [39]. Therefore, we hypothesized that POSTN affects CD70 expression through the αvβ3/PI3K/AKT/NFκB p65 pathway. Immunoblot analysis confirmed that the activation of AKT and NFκB p65 phosphorylation was dramatically increased (Fig. 7B) and that NF-κB p65 nuclear translocation was increased (Fig. 7C) by rhPOSTN treatment in microglia, indicating that POSTN regulates the activity of NFκB p65 in microglia. We subsequently investigated whether CD70 is regulated by p-AKT and p-NFκB p65. We used MK-2206 (10 µM, 48 h) to inhibit AKT phosphorylation (Fig. 7D) and JSH-23 (10 µM, 48 h) to inhibit the nuclear translocation of NFκB p65 (Fig. 7E). Immunoblot analysis revealed that the increase in CD70 expression induced by rhPOSTN was reversed by both MK-2206 and JSH-23 (Fig. 7F). We next investigated whether integrin αVβ3 is required for POSTN-mediated activation of the AKT/NFKB p65/CD70 pathway. Treatment of microglia with cyclo(RGDyK) (100 nM) for 48 h significantly attenuated the activation of the PI3K/AKT/NFκB p65/CD70 pathway induced by rhPOSTN (Fig. 7G). These observations suggest that CD70 expression is induced via the POSTN/αvβ3/PI3K/AKT/NFκB p65 axis.

Moreover, to determine the regulatory role of NFκB p65 in CD70 transcription, we performed ChIP-seq. Our results showed that NFκB p65 binds directly to the CD70 promoter. (Fig. 7H). To further confirm that NFκB p65 is a transcriptional enhancer of CD70, we inhibited the nuclear translocation of NFκB in microglia and found that the rhPOSTN-induced increase in CD70 mRNA expression was significantly attenuated (Fig. 7I). ChIP‒PCR analysis was then performed using IgG and an anti-NFκB p65 antibody and five pairs of CD70 promoter-specific genomic PCR primers. We detected enrichment of NFκB p65 at the CD70 promoter, and blocking the nuclear translocation of NFκB via JSH-23 abolished the enrichment of NFκB p65 (Fig. 7J). In addition to confirming its effect on HMC3 cells, we also validated the regulatory effect of POSTN on the αvβ3/PI3K/AKT/NFκB p65/CD70 pathway in the mouse-derived microglial cell line BV2, with results similar to those obtained for the HMC3 cell line (Supplementary Fig. S6C-K). Taken together, these findings indicate that CD70 transcription is regulated by NFκB p65 downstream of the POSTN/αvβ3/PI3K/AKT pathway.

### Knockdown of POSTN in glioma reverses tumor-associated immunosuppression and impairs tumor growth

**Fig. 8:**
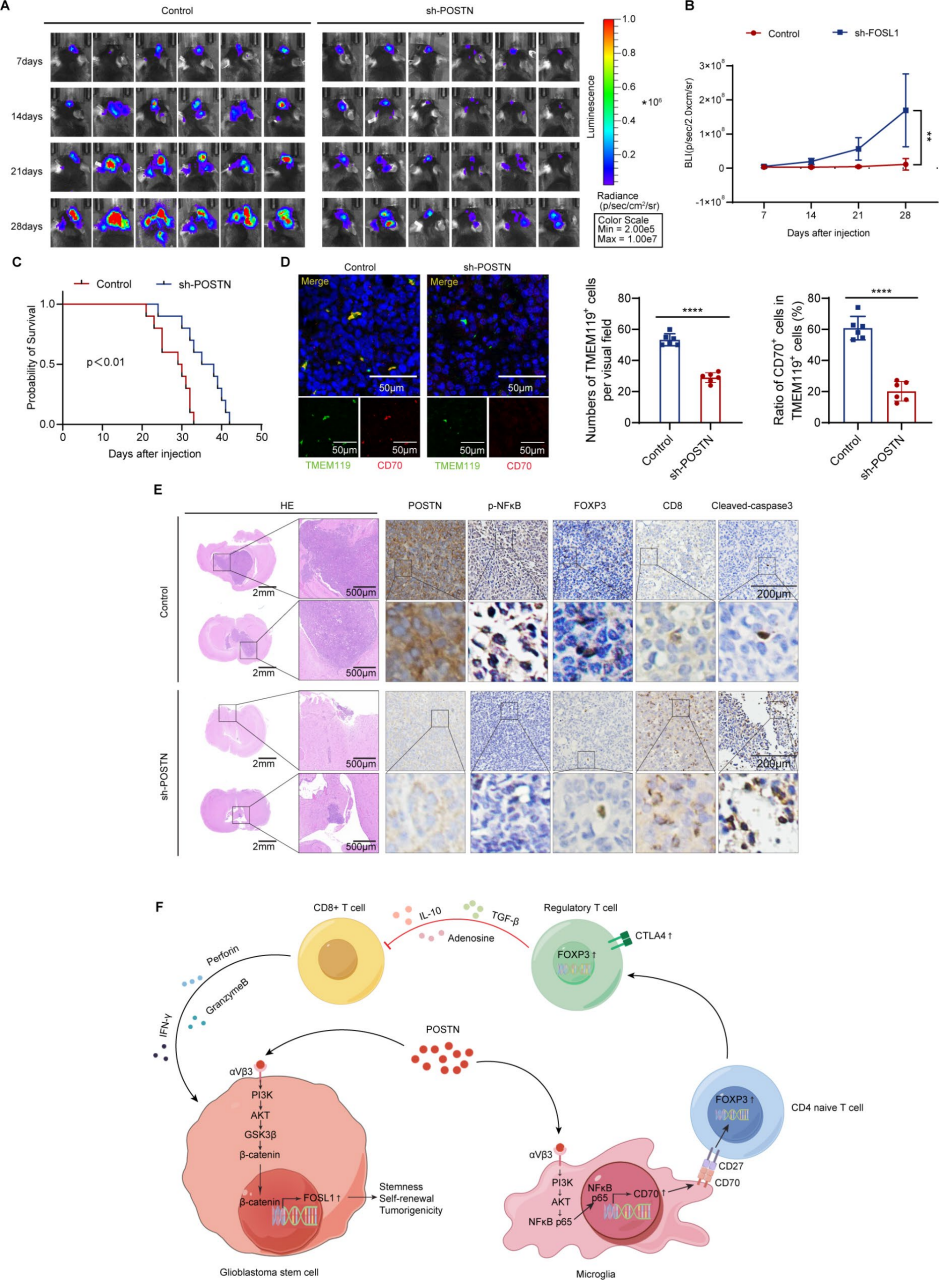
Knockdown of POSTN in glioma reverses tumor-associated immunosuppression and impairs tumor growth. **A** and **B**, In vivo bioluminescence images (**A**) and quantitative analysis (**B**) of xenografts derived from luciferase-labeled GL261 cells expressing control or sh-POSTN on the indicated days after implantation; *n* = 8 mice. The data are presented as the means ± SDs. Two-tailed Student’s t test. ***p < 0.001. **C**, Kaplan–Meier survival curves of mice implanted with GL261 cells expressing sh-Control or sh-POSTN; *n* = 10 mice. Log–rank test. **D**, Left: Immunofluorescence staining of the microglial markers TMEM119 (green) and CD70 (red) in tumors derived from GL261 cells expressing sh-Control or sh-POSTN. Scale bar, 50 μm. Right: The number of TMEM119^+^ cells in each field of view and the proportion of CD70^+^ TMEM119^+^ cells; *n* = 6. **E**, Representative images of HE staining and IHC staining for POSTN, p-NFκB p65, CD70, FOXP3, CD8 and cleaved caspase-3 in sections of GL261 cell xenografts from each group. Scale bars: 2 mm for HE staining (left), 500 μm for HE staining (right), 200 μm for IHC staining (top) and 200 μm for IHC staining (bottom). **F**, POSTN is a key mediator of GSC–microglia crosstalk in GBM. POSTN secreted from GSCs promotes GSC self-renewal and tumor growth via activation of the PI3K/AKT/β-catenin/FOSL1 pathway. In addition to its intrinsic effects on GSCs, POSTN can recruit microglia and upregulate CD70 expression in microglia through the PI3K/AKT/NFκB pathway, which in turn promotes Treg development and functionality and supports the formation of an immunosuppressive TME

To verify the tumor-promoting effect of POSTN in GBM, we knocked down POSTN in the murine glioma cell line GL261 and orthotopically injected normal and POSTN-knockdown GL261 cells into C57BL/6J mice. In vivo bioluminescence imaging indicated that POSTN knockdown significantly inhibited the growth of GL261-derived xenografts (Fig. 8A and B) and that, compared with the mice in the control group, the mice in the POSTN knockdown group had a significantly longer survival time (Fig. 8C). POSTN knockdown significantly suppressed microglial recruitment and CD70 expression in microglia, as demonstrated by TMEM119 and CD70 immunofluorescence analyses (Fig. 8D). In addition, HE and immunohistochemical staining revealed that POSTN knockdown reduced the number of Treg cells, as indicated by the presence of FOXP3, increased the number of CD8^+^ T cells, increased the number of apoptotic cells (as indicated by the presence of cleaved caspase-3) and reduced tumor growth (Fig. 8E). Collectively, these data indicate that the regulation of the PI3K/AKT/NFκB p65/CD70 signaling pathway in microglia by POSTN is an important mechanism underlying the Treg-mediated immunosuppressive microenvironment in GBM.

## Discussion

GBM is an immunosuppressive malignancy driven by GSCs. The interplay between GSCs and immunosuppressive microglia plays crucial roles in promoting GBM progression. This study aimed to clarify the molecular mechanisms underlying this crosstalk. In this study, we identified POSTN as a key mediator of GSC–microglia crosstalk in GBM (Fig. 8F). POSTN secreted from GSCs promotes GSC self-renewal and tumor growth via activation of the PI3K/AKT/β-catenin/FOSL1 pathway. In addition to its intrinsic effects on GSCs, POSTN can recruit microglia and upregulate CD70 expression in microglia through the PI3K/AKT/NFκB pathway, which in turn promotes Treg development and functionality and supports the development of an immunosuppressive TME. Taken together, our findings reveal the molecular mechanisms underlying GSC–microglia symbiosis and confirm that targeting POSTN constitutes an attractive therapeutic strategy.

POSTN is expressed at extremely low levels in healthy tissues but is overexpressed in cancers [20, 21, 40]. In this study, immunohistochemical, immunoblotting and PCR analyses of normal brain tissue and glioma tissue confirmed that the expression of POSTN was significantly greater in glioma tissues than in normal brain tissues. Moreover, among glioma tissues, GBM tissues presented the highest expression of POSTN. These results were verified via bioinformatics analysis of TCGA, CGGA and Rembrandt data. In addition, survival analysis revealed that high POSTN expression was associated with poor prognosis in glioma patients, suggesting that POSTN may play a protumor role in GBM.

GSCs are a subpopulation of GBM cells with self-renewal, multilineage differentiation and tumorigenic abilities. GSCs exhibit invasive and angiogenic potential, increased cellular plasticity, and resistance to radiotherapy and chemotherapy, and they also play critical roles in GBM progression [4, 7]. POSTN is highly expressed in GSCs, and POSTN knockdown significantly impairs the survival of xenografted GSCs [40, 41]; however, the underlying mechanism involved remains to be elucidated. The results of our gain- and loss-of-function experiments indicated that increasing POSTN expression in POSTN-low GSCs promoted their self-renewal and proliferation, whereas reducing POSTN expression in POSTN-high GSCs had the opposite effects. Through RNA-seq analysis and experimental verification, we confirmed that POSTN secreted by GSCs mainly upregulates the expression of FOSL1 through aberrant activation of the PI3K/AKT/β-catenin signaling pathway, thereby promoting the self-renewal and tumorigenicity of GSCs. PI3K/AKT signaling, β-catenin and FOSL1 are important factors that promote GSC proliferation and self-renewal [28, 29, 42]; thus, these results indicate that POSTN is a potential therapeutic target for regulating the stemness of GSCs.

GSCs and their interactions with microglia strongly influence GBM progression. On the one hand, factors secreted by GSCs, such as TFPI2 [31] and OLFML3 [43], actively recruit microglia to the tumor site and induce an immunosuppressive phenotype in microglia; on the other hand, microglia in tumors lead to the formation of an immunosuppressive glioma microenvironment by inhibiting antigen presentation to T lymphocytes [44], synthesizing the immunosuppressive cytokine IL-10 [12], and expressing the immune checkpoint molecules PD-L1/2, CLE2D and CD80/86 [9]. The immunosuppressive glioma microenvironment suppresses the killing of GSCs by immune cells and promotes the progression of GBM. Previous studies have shown that POSTN secreted by GSCs can recruit monocyte-derived macrophages from peripheral blood and promote the malignant growth of GBM [22]. However, the role of POSTN in regulating microglial properties has not yet been studied. Our study revealed that POSTN significantly increased microglial migration and recruitment into tumors. Moreover, we found that POSTN increases the expression of CD70 by activating the PI3K/AKT/NFκB signaling pathway in microglia to affect the GBM microenvironment. Recent studies have revealed high CD70 protein expression in numerous types of solid tumors. CD70 promotes the development of Tregs and promotes the long-term survival and suppressive activity of mature Tregs through the CD70–CD27 axis [33, 34]. However, most previous studies on CD70 have been limited to tumors, and whether the upregulation of CD70 expression in microglia affects the GBM microenvironment has not been reported.

In this study, we discovered that POSTN-mediated upregulation of CD70 promotes the development, activation and maintenance of Tregs in the GBM microenvironment. CD70 knockdown in microglia inhibits the development and immunosuppressive activity of Tregs, increases the expansion and tumoricidal capacity of CD8^+^ T cells, and ameliorates the immunosuppressive phenotype of the TME. Considering that in many tumors, the CD70 protein is also overexpressed by the tumor cells themselves, we further studied the effect of POSTN on the CD70 level in GSCs. Interestingly, we found that POSTN secreted by GSCs did not significantly affect the CD70 protein level in the GSCs themselves, indicating that POSTN modulates the biological responses of different cells in different manners in the complex GBM microenvironment.

In recent years, immunotherapeutic strategies have provided new hope for the treatment of GBM; however, the immunosuppressive TME limits the effectiveness of immunotherapy for GBM. For example, immunotherapy targeting the immunosuppressive checkpoint programmed cell death protein 1 (PD-1) has achieved promising results in several cancers, but anti-PD-1 antibodies have limited therapeutic efficacy in GBM patients [45, 46]. Despite the increased T-cell infiltration and remodeling of the GBM microenvironment observed after neoadjuvant anti-PD-1 treatment, the main immune cells infiltrating the GBM microenvironment are still macrophages and monocytes [45]. Importantly, all macrophages and monocyte populations maintain persistently high expression levels of T-cell-suppressive checkpoint molecules, such as CD86, which may drive an increase in the number of progenitor exhausted T cells and limit the magnitude and persistence of antitumor T-cell activity. In this study, we confirmed that POSTN recruits microglia and promotes the expression of the immune checkpoint CD70 in microglia. Considering these results and previous reports indicating that POSTN recruits M2 TAMs [22], we hypothesize that anti-POSTN/anti-PD-1 combination strategies will exhibit increased therapeutic efficacy, but this hypothesis needs verification.

## Conclusions

The interplay between GSCs and immunosuppressive microglia plays crucial roles in promoting the malignant growth of GBM, and disrupting the crosstalk between GSCs and immunosuppressive microglia is a promising option for treating GBM. In conclusion, we identified POSTN as a key regulator that mediates the molecular crosstalk between GSCs and immunosuppressive microglia in the GBM microenvironment. POSTN activates the PI3K/AKT/β-catenin/FOSL1 pathway in an autocrine manner to promote GSC self-renewal and tumor growth. In addition, POSTN recruits microglia in a paracrine manner and upregulates the expression of CD70 in microglia through the PI3K/AKT/NFκB pathway, thereby promoting the development and function of Tregs and facilitating the formation of an immunosuppressive TME. Our findings indicate that targeting the POSTN gene may be a promising approach for ablating GSCs, disrupting the immunosuppressive environment and overcoming treatment resistance in patients with GBM.

## Electronic supplementary material

Below is the link to the electronic supplementary material.

Supplementary Material 1: Supplementary Fig. 1. POSTN overexpression promotes GSC self-renewal and proliferation. **A**, UMAP of single-cell RNA-seq data from GBM tissue cells (*n* = 19995). **B**, Expression of POSTN in UMAP (left) and different cell types (right). **C**, UMAP of single-cell RNA-seq data of tumor cells in GBM tissue. **D**, Expression of POSTN in UMAP (left) and different cell types (right). **E**, UMAP of the single-cell analysis dataset (GSE139448), cells = 12152. **F**, Expression of POSTN in UMAP (left) and different cell types (right). **G**, UMAP of tumor cells in the single-cell analysis dataset (GSE139448). **H**, Expression of POSTN in UMAP (left) and different cell types (right). **I**, Immunoblot analysis of POSTN in lysates from SHG141 GSCs and SHG144 GSCs in the control, overexpression vector control (OE-Control) and OE-POSTN groups. **J**, ELISA for the detection of secreted POSTN in the culture supernatant of GSCs expressing control, OE-Control or OE-POSTN. *n* = 3 biological replicates. **K**, Representative images of tumorspheres formed by SHG141 GSCs and SHG144 GSCs in the control, OE-POSTN, or rhPOSTN (1 µg ml^− 1^, 48 h) groups. Scale bar, 200 μm. **L**, Quantification of the number and diameter of SHG141 GSC- and SHG144 GSC-derived tumorspheres treated with control, OE-POSTN, or rhPOSTN (1 µg ml^− 1^, 48 h). *n* = 6 biological replicates. **M**, CCK-8 assay of SHG141 GSCs and SHG144 GSCs in the control, OE-POSTN, and rhPOSTN (1 µg ml^− 1^, 48 h) groups. *n* = 6 biological replicates. **N**, Representative images of immunofluorescence staining for CD133 and Nestin in SHG141 GSC- and SHG144 GSC-derived tumorspheres treated with control, OE-POSTN, or rhPOSTN (1 µg ml^− 1^, 48 h). **O** and **P**, Immunoblot analysis of CD133, Nestin and SOX2 in SHG141 GSCs and SHG144 GSCs in the control, OE-POSTN, and rhPOSTN (1 µg ml^− 1^, 48 h) groups. CD133, Nestin and SOX2 protein levels were quantified (**G**). The error bars indicate the means ± SDs (**J**,** L** and **P**). Two-tailed Student’s t test (**J**,** L**,** M** and **P**). *p < 0.05, **p < 0.01, ***p < 0.001, and ****p < 0.0001.

Supplementary Material 2: Supplementary Fig. 2. FOSL1 overexpression promotes GSC self-renewal and proliferation. **A**, Correlations between the expression of POSTN and the expression of TNFSF13B, ARHGEF2, NR1D1, TIMM8B, and DNAAF4 in the TCGA dataset; *n* = 675. **B**, Images showing the expression of FOSL1 in human normal brain tissue and glioma tissue samples via immunohistochemical staining. Scale bar, 200 μm (top), 20 μm (bottom). **C**, Kaplan–Meier survival analysis of patients stratified by the median FOSL1 level in different glioma datasets: the TCGA, CGGA, and Rembrandt datasets. TCGA datasets: low POSTN expression, *n* = 258; high POSTN expression, *n* = 354. CGGA datasets: low POSTN expression, *n* = 419; high POSTN expression, *n* = 551. Rembrandt datasets: low POSTN expression, *n* = 228; high POSTN expression, *n* = 71. **D** and **E**, Immunoblot analysis of FOSL1 in lysates from SHG141 GSCs and SHG144 GSCs in the control, OE-Control and OE-FOSL1 groups. **F**, Representative images of tumorspheres formed by SHG141 GSCs and SHG144 GSCs in the control and OE-FOSL1 groups. Scale bar, 200 μm. **G**, Quantification of the number and diameter of SHG141 GSC-derived and SHG144 GSC-derived tumorspheres in the control and OE-FOSL1 groups. *n* = 6 biological replicates. **H**, CCK-8 assay of SHG141 GSCs and SHG144 GSCs in the control and OE-FOSL1 groups. *n* = 6 biological replicates. **I**, Representative images of immunofluorescence staining for CD133 and Nestin in SHG141 GSC- and SHG144 GSC-derived tumorspheres in the control and OE-FOSL1 groups. **J**, Immunoblot analysis of CD133, Nestin and SOX2 in SHG141 GSCs and SHG144 GSCs in the control and OE-FOSL1 groups.

Supplementary Material 3: Supplementary Fig. 3. FOSL1 is a crucial factor that mediates the ability of POSTN to promote GSC self-renewal. **A**, Representative images of tumorspheres formed by SHG141 GSCs and SHG144 GSCs expressing control or sh-FOSL1 and treated with or without rhPOSTN (1 µg ml^− 1^) for 48 h. Scale bar, 200 μm. **B**, Quantification of the number and diameter of SHG141 GSC- and SHG144 GSC-derived tumorspheres expressing control or sh-FOSL1 and treated with or without rhPOSTN (1 µg ml^− 1^) for 48 h; *n* = 6 biological replicates. **C**, CCK-8 assay of SHG141 GSCs and SHG144 GSCs expressing control or sh-FOSL1 and treated with or without rhPOSTN (1 µg ml^− 1^) for 48 h; *n* = 6 biological replicates. **D**, Representative images of immunofluorescence staining of CD133 and Nestin in SHG141 GSCs and SHG144 GSCs expressing control or sh-FOSL1 and treated with or without rhPOSTN (1 µg ml^− 1^) for 48 h. Scale bar, 50 μm. **E** and **F**, Immunoblot analysis of CD133, Nestin and SOX2 in SHG141 GSCs and SHG144 GSCs expressing control or sh-FOSL1 and treated with or without rhPOSTN (1 µg ml^− 1^) for 48 h. CD133, Nestin and SOX2 protein levels were quantified (**F**). The error bars indicate the means ± SDs (**B**,** C**, and **F**). Two-tailed Student’s t test (**B**,** C**, and **F**). *p < 0.05, **p < 0.01, ***p < 0.001, and ****p < 0.0001.

Supplementary Material 4: Supplementary Fig. 4. CD70 is highly expressed in GBM and is associated with poor outcomes in glioma patients. **A**, Volcano plot displaying the differentially expressed genes identified via RNA-seq analysis of HMC3 cells treated with or without rhPOSTN (1 µg ml^− 1^) for 48 h. **B**, Correlations between POSTN and CD70 expression in the TCGA and CGGA datasets. TCGA datasets: *n* = 675. CGGA datasets: *n* = 1018. R and P values were determined by Pearson correlation analysis. **C**, Kaplan–Meier survival analysis of patients stratified by CD70 expression in different glioma datasets: the TCGA, CGGA, and Rembrandt datasets. TCGA datasets: low POSTN expression, *n* = 308; high POSTN expression, *n* = 307. CGGA datasets: low POSTN expression, *n* = 485; high POSTN expression, *n* = 485. **D**, Correlation between CD70 expression and regulatory T-cell infiltration in the TCGA and CGGA datasets. TCGA datasets: *n* = 675. CGGA datasets: *n* = 1018. R and P values were determined by Pearson correlation analysis.

Supplementary Material 5: Supplementary Fig. 5. Treating microglia with rhPOSTN promotes treg development and function. **A**, Immunophenotyping of freshly isolated naïve CD4^+^ T cells according to CD4 expression. **B**, Immunophenotyping of TCR-activated naïve CD4^+^ T cells according to CD25 expression. **C**, Immunophenotyping of naïve CD4^+^ T cells after 3 days of induced Treg differentiation without HMC3 cell coculture. **D**, Proportions of FOXP3^+^ Tregs and FOXP3^+^CTLA4^+^ activated Tregs; *n* = 3. E, Immunoblots showing CD70 expression in HMC3 cells expressing control or sh-POSTN. **F**, Changes in the levels of the immunosuppressive factors IL-10, TGF-β1, and adenosine in HMC3 cells treated with the control, rhPOSTN (1 µg ml^− 1^), sh-CD70 or rhCD70 (800 ng ml^− 1^) for 48 h. IL-10 and TGF-β1 were measured by ELISA, and adenosine was measured via a fluorescent adenosine assay, *n* = 3 **G**, RNA-seq analysis of CD70 in SHG143 GSCs expressing control or sh-POSTN and in SHG141 GSCs treated with control or rhPOSTN (1 µg ml^− 1^) for 48 h. **H**, Immunoblots showing CD70 expression in SHG142 GSCs and SHG143 GSCs expressing control or sh-POSTN. **I**, Immunoblots showing CD70 expression in SHG141 GSCs and SHG144 GSCs treated with control or rhPOSTN (1 µg ml^− 1^) for 48 h. The error bars indicate the means ± SDs (**D**,** F** and **G**). Two-tailed Student’s t test (**F** and **G**). #, nonsignificant; **p < 0.01, ***p < 0.001, and ****p < 0.0001.

Supplementary Material 6: Supplementary Fig. 6. CD70 expression is regulated by NFκB in the POSTN/αvβ3/PI3K/AKT pathway in BV2 cells. **A**, qRT‒PCR analysis of CD70 mRNA expression in HMC3 cells expressing control, sh-NFκB p65, sh-SUZ12, or sh-MITF; *n* = 3 for all groups. **B**, Immunoblots showing CD70 expression in HMC3 cells treated with control, rhPOSTN (1 µg ml^− 1^) or rhPOSTN (1 µg ml^− 1^) + sh-NFκB p65/sh-SUZ12/sh-MITF for 48 h. **C** and **D**, Representative images (**C**) and quantitative analysis (**D**) of the relative migration of BV2 cells following stimulation with recombinant mouse POSTN (rmPOSTN); *n* = 6. Scale bars, 200 μm. **E**, Immunoblots showing the p-AKT (Ser473), AKT, p-NFκB p65, NFκB p65 and CD70 levels in BV2 cells treated with or without rmPOSTN (1 µg ml^− 1^) for 48 h. **F**, Nuclear lysate was used to determine the p-NFκB p65 level in BV2 cells treated with or without rmPOSTN (1 µg ml^− 1^) for 48 h. **G**, Representative images of immunofluorescence staining of CD70 in BV2 cells treated with control or rmPOSTN (1 µg ml^− 1^, 48 h). Scale bar, 50 μm. **H**, Immunoblot analysis of р-AKT (Ser473) and AKT in BV2 cells treated with the control, DMSO or MK2206 (10 µM) for 48 h. **I**, Total lysate and the nuclear fraction were used to measure p-NFκB p65 levels in BV2 cells after treatment with JSH-23 (10 µM) for 48 h. **J**, Immunoblots showing CD70 expression in BV2 cells treated with control, rmPOSTN (1 µg ml^− 1^) or rmPOSTN (1 µg ml^− 1^) + MK2206 (10 µM)/JSH-23 (10 µM) for 48 h. **K**, Immunoblot analysis of p-AKT (Ser473), p-NFκB p65, and CD70 in BV2 cells treated with control, rmPOSTN (1 µg ml^− 1^) or rmPOSTN (1 µg ml^− 1^) + Cyclo(RGDyK) (100 nM) for 48 h. The error bars indicate the means ± SDs (**A**, and **D**). Two-tailed Student’s t test (**A**, and **D**). #, nonsignificant; *p < 0.05, **p < 0.01, and ****p < 0.0001.

Supplementary Material 7

Supplementary Material 8

## Data Availability

The data generated in this study are available from the corresponding author upon reasonable request. RNA-seq and ChIP-seq data are available in Gene Expression Omnibus (GEO) with accession code GSE273464, GSE273250, GSE273251 and GSE273252.

## Abbreviations

GBM: Glioblastoma
TMZ: Temozolomide
OS: Overall Survival
GSC: Glioblastoma Stem Cell
TME: Tumor Microenvironment
TGF-β1: Transforming Growth Factor-beta1
mTOR: Mammalian Target Of Rapamycin
sCSF-1: Soluble Colony-Stimulating Factor
MIC-1: Macrophage-Inhibiting Cytokine-1
WISP1: Wnt1-Inducible Signaling Pathway Protein 1
TAMs: Tumor-Associated Macrophages
Treg: Regulatory T-cell
SD: Standard Deviation
qRT‒PCR: Quantitative Real-Time PCR
TCGA: The Cancer Genome Atlas
CGGA: Chinese Glioma Genome Atlas
rhPOSTN: Recombinant Human POSTN
rmPOSTN: Recombinant Mouse POSTN
rhCD70: Recombinant Human CD70
FOSL1: FOS-Like antigen 1
CCK-8: Cell Counting Kit-8
RNA-seq: RNA sequencing
KEGG: Kyoto Encyclopedia of Genes and Genomes
p-AKT: Phospho-AKT
p-GSK3β: Phospho-GSK3β
TCR: T-Cell Receptor
ChEA: Chromatin immunoprecipitation Enrichment Analysis
PD-1: Programmed cell Death protein 1

## References

[1] Ostrom QT, Price M, Neff C, Cioffi G, Waite KA, Kruchko C, Barnholtz-Sloan JS. CBTRUS Statistical Report: primary brain and other Central Nervous System tumors diagnosed in the United States in 2016–2020. Neuro Oncol. 2023;25:iv1–199. 37793125 10.1093/neuonc/noad149 PMC10550277

[2] Stupp R, Mason WP, van den Bent MJ, Weller M, Fisher B, Taphoorn MJ, Belanger K, Brandes AA, Marosi C, Bogdahn U, et al. Radiotherapy plus concomitant and adjuvant temozolomide for glioblastoma. N Engl J Med. 2005;352:987–96. 15758009 10.1056/NEJMoa043330

[3] Kreso A, Dick JE. Evolution of the cancer stem cell model. Cell Stem Cell. 2014;14:275–91. 24607403 10.1016/j.stem.2014.02.006

[4] Suvà ML, Tirosh I. The glioma stem cell model in the era of single-cell Genomics. Cancer Cell. 2020;37:630–6. 32396858 10.1016/j.ccell.2020.04.001

[5] Quail DF, Joyce JA. Microenvironmental regulation of tumor progression and metastasis. Nat Med. 2013;19:1423–37. 24202395 10.1038/nm.3394 PMC3954707

[6] Lathia JD, Heddleston JM, Venere M, Rich JN. Deadly teamwork: neural cancer stem cells and the tumor microenvironment. Cell Stem Cell. 2011;8:482–5. 21549324 10.1016/j.stem.2011.04.013 PMC3494093

[7] Cheng L, Huang Z, Zhou W, Wu Q, Donnola S, Liu JK, Fang X, Sloan AE, Mao Y, Lathia JD, et al. Glioblastoma stem cells generate vascular pericytes to support vessel function and tumor growth. Cell. 2013;153:139–52. 23540695 10.1016/j.cell.2013.02.021 PMC3638263

[8] Li D, Zhang Q, Li L, Chen K, Yang J, Dixit D, Gimple RC, Ci S, Lu C, Hu L, et al. β2-Microglobulin maintains Glioblastoma Stem cells and induces M2-like polarization of Tumor-Associated macrophages. Cancer Res. 2022;82:3321–34. 35841593 10.1158/0008-5472.CAN-22-0507

[9] Mirzaei R, Yong VW. Microglia-T cell conversations in brain cancer progression. Trends Mol Med. 2022;28:951–63. 36075812 10.1016/j.molmed.2022.08.006

[10] Yeo AT, Rawal S, Delcuze B, Christofides A, Atayde A, Strauss L, Balaj L, Rogers VA, Uhlmann EJ, Varma H, et al. Single-cell RNA sequencing reveals evolution of immune landscape during glioblastoma progression. Nat Immunol. 2022;23:971–84. 35624211 10.1038/s41590-022-01215-0 PMC9174057

[11] Ye XZ, Xu SL, Xin YH, Yu SC, Ping YF, Chen L, Xiao HL, Wang B, Yi L, Wang QL, et al. Tumor-associated microglia/macrophages enhance the invasion of glioma stem-like cells via TGF-β1 signaling pathway. J Immunol. 2012;189:444–53. 22664874 10.4049/jimmunol.1103248

[12] Ravi VM, Neidert N, Will P, Joseph K, Maier JP, Kückelhaus J, Vollmer L, Goeldner JM, Behringer SP, Scherer F, et al. T-cell dysfunction in the glioblastoma microenvironment is mediated by myeloid cells releasing interleukin-10. Nat Commun. 2022;13:925. 35177622 10.1038/s41467-022-28523-1 PMC8854421

[13] Dumas AA, Pomella N, Rosser G, Guglielmi L, Vinel C, Millner TO, Rees J, Aley N, Sheer D, Wei J, et al. Microglia promote glioblastoma via mTOR-mediated immunosuppression of the tumour microenvironment. EMBO J. 2020;39:e103790. 32567735 10.15252/embj.2019103790 PMC7396846

[14] Wu A, Wei J, Kong LY, Wang Y, Priebe W, Qiao W, Sawaya R, Heimberger AB. Glioma cancer stem cells induce immunosuppressive macrophages/microglia. Neuro Oncol. 2010;12:1113–25. 20667896 10.1093/neuonc/noq082 PMC3098021

[15] Tao W, Chu C, Zhou W, Huang Z, Zhai K, Fang X, Huang Q, Zhang A, Wang X, Yu X, et al. Dual role of WISP1 in maintaining glioma stem cells and tumor-supportive macrophages in glioblastoma. Nat Commun. 2020;11:3015. 32541784 10.1038/s41467-020-16827-z PMC7295765

[16] Wasik A, Ratajczak-Wielgomas K, Badzinski A, Dziegiel P, Podhorska-Okolow M. The role of Periostin in Angiogenesis and Lymphangiogenesis in Tumors. Cancers (Basel). 2022;14:4225. 36077762 10.3390/cancers14174225 PMC9454705

[17] Conway SJ, Izuhara K, Kudo Y, Litvin J, Markwald R, Ouyang G, Arron JR, Holweg CT, Kudo A. The role of periostin in tissue remodeling across health and disease. Cell Mol Life Sci. 2014;71:1279–88. 24146092 10.1007/s00018-013-1494-y PMC3949008

[18] Horiuchi K, Amizuka N, Takeshita S, Takamatsu H, Katsuura M, Ozawa H, Toyama Y, Bonewald LF, Kudo A. Identification and characterization of a novel protein, periostin, with restricted expression to periosteum and periodontal ligament and increased expression by transforming growth factor beta. J Bone Min Res. 1999;14:1239–49. 10.1359/jbmr.1999.14.7.1239 10404027

[19] Hakuno D, Kimura N, Yoshioka M, Mukai M, Kimura T, Okada Y, Yozu R, Shukunami C, Hiraki Y, Kudo A, et al. Periostin advances atherosclerotic and rheumatic cardiac valve degeneration by inducing angiogenesis and MMP production in humans and rodents. J Clin Invest. 2010;120:2292–306. 20551517 10.1172/JCI40973 PMC2898587

[20] Cui D, Huang Z, Liu Y, Ouyang G. The multifaceted role of periostin in priming the tumor microenvironments for tumor progression. Cell Mol Life Sci. 2017;74:4287–91. 28884337 10.1007/s00018-017-2646-2 PMC11107730

[21] Tian B, Zhang Y, Zhang J. Periostin is a new potential prognostic biomarker for glioma. Tumour Biol. 2014;35:5877–83. 24719188 10.1007/s13277-014-1778-3

[22] Zhou W, Ke SQ, Huang Z, Flavahan W, Fang X, Paul J, Wu L, Sloan AE, McLendon RE, Li X, et al. Periostin secreted by glioblastoma stem cells recruits M2 tumour-associated macrophages and promotes malignant growth. Nat Cell Biol. 2015;17:170–82. 25580734 10.1038/ncb3090 PMC4312504

[23] Castellani G, Buccarelli M, D’Alessandris QG, Ilari R, Cappannini A, Pedini F, Boe A, Lulli V, Parolini I, Giannetti S, et al. Extracellular vesicles produced by irradiated endothelial or Glioblastoma stem cells promote tumor growth and vascularization modulating tumor microenvironment. Cancer Cell Int. 2024;24:72. 38347567 10.1186/s12935-024-03253-0 PMC10863174

[24] Tao Z, Li X, Wang H, Chen G, Feng Z, Wu Y, Yin H, Zhao G, Deng Z, Zhao C, et al. BRD4 regulates self-renewal ability and tumorigenicity of glioma-initiating cells by enrichment in the Notch1 promoter region. Clin Transl Med. 2020;10:e181. 33135348 10.1002/ctm2.181 PMC7533052

[25] Xie Y, He L, Lugano R, Zhang Y, Cao H, He Q, Chao M, Liu B, Cao Q, Wang J, et al. Key molecular alterations in endothelial cells in human glioblastoma uncovered through single-cell RNA sequencing. JCI Insight. 2021;6:e150861. 34228647 10.1172/jci.insight.150861 PMC8410070

[26] Liu H, Sun Y, Zhang Q, Jin W, Gordon RE, Zhang Y, Wang J, Sun C, Wang ZJ, Qi X, et al. Pro-inflammatory and proliferative microglia drive progression of glioblastoma. Cell Rep. 2021;36:109718. 34525361 10.1016/j.celrep.2021.109718

[27] Marques C, Unterkircher T, Kroon P, Oldrini B, Izzo A, Dramaretska Y, Ferrarese R, Kling E, Schnell O, Nelander S, et al. NF1 regulates mesenchymal glioblastoma plasticity and aggressiveness through the AP-1 transcription factor FOSL1. Elife. 2021;10:e64846. 34399888 10.7554/eLife.64846 PMC8370767

[28] Chen Z, Wang S, Li HL, Luo H, Wu X, Lu J, Wang HW, Chen Y, Chen D, Wu WT, et al. FOSL1 promotes proneural-to-mesenchymal transition of glioblastoma stem cells via UBC9/CYLD/NF-κB axis. Mol Ther. 2022;30:2568–83. 35351656 10.1016/j.ymthe.2021.10.028 PMC9263249

[29] Pecce V, Verrienti A, Fiscon G, Sponziello M, Conte F, Abballe L, Durante C, Farina L, Filetti S, Paci P. The role of FOSL1 in stem-like cell reprogramming processes. Sci Rep. 2021;11:14677. 34282187 10.1038/s41598-021-94072-0 PMC8290037

[30] Nayakanti SR, Friedrich A, Sarode P, Jafari L, Maroli G, Boehm M, Bourgeois A, Grobs Y, Khassafi F, Kuenne C, et al. Targeting Wnt-β-Catenin-FOSL signaling ameliorates right ventricular remodeling. Circ Res. 2023;132:1468–85. 37042252 10.1161/CIRCRESAHA.122.321725

[31] Pang L, Dunterman M, Guo S, Khan F, Liu Y, Taefi E, Bahrami A, Geula C, Hsu WH, Horbinski C, et al. Kunitz-type protease inhibitor TFPI2 remodels stemness and immunosuppressive tumor microenvironment in glioblastoma. Nat Immunol. 2023;24:1654–70. 37667051 10.1038/s41590-023-01605-y PMC10775912

[32] Xuan W, Hsu WH, Khan F, Dunterman M, Pang L, Wainwright DA, Ahmed AU, Heimberger AB, Lesniak MS, Chen P. Circadian Regulator CLOCK drives immunosuppression in Glioblastoma. Cancer Immunol Res. 2022;10:770–84. 35413115 10.1158/2326-6066.CIR-21-0559 PMC9177794

[33] Gong L, Luo J, Zhang Y, Yang Y, Li S, Fang X, Zhang B, Huang J, Chow LK, Chung D, et al. Nasopharyngeal carcinoma cells promote regulatory T cell development and suppressive activity via CD70-CD27 interaction. Nat Commun. 2023;14:1912. 37024479 10.1038/s41467-023-37614-6 PMC10079957

[34] Flieswasser T, Van den Eynde A, Van Audenaerde J, De Waele J, Lardon F, Riether C, de Haard H, Smits E, Pauwels P, Jacobs J. The CD70-CD27 axis in oncology: the new kids on the block. J Exp Clin Cancer Res. 2022;41:12. 34991665 10.1186/s13046-021-02215-y PMC8734249

[35] Ooi YC, Tran P, Ung N, Thill K, Trang A, Fong BM, Nagasawa DT, Lim M, Yang I. The role of regulatory T-cells in glioma immunology. Clin Neurol Neurosurg. 2014;119:125–32. 24582432 10.1016/j.clineuro.2013.12.004

[36] Tanaka A, Sakaguchi S. Regulatory T cells in cancer immunotherapy. Cell Res. 2017;27:109–18. 27995907 10.1038/cr.2016.151 PMC5223231

[37] Kuleshov MV, Jones MR, Rouillard AD, Fernandez NF, Duan Q, Wang Z, Koplev S, Jenkins SL, Jagodnik KM, Lachmann A, et al. Enrichr: a comprehensive gene set enrichment analysis web server 2016 update. Nucleic Acids Res. 2016;44:W90–7. 27141961 10.1093/nar/gkw377 PMC4987924

[38] Wang Z, An J, Zhu D, Chen H, Lin A, Kang J, Liu W, Kang X. Periostin: an emerging activator of multiple signaling pathways. J Cell Commun Signal. 2022;16:515–30. 35412260 10.1007/s12079-022-00674-2 PMC9733775

[39] Huang K, Liu X, Li Y, Wang Q, Zhou J, Wang Y, Dong F, Yang C, Sun Z, Fang C, et al. Genome-wide CRISPR-Cas9 screening identifies NF-κB/E2F6 responsible for EGFRvIII-Associated Temozolomide Resistance in Glioblastoma. Adv Sci (Weinh). 2019;6:1900782. 31508283 10.1002/advs.201900782 PMC6724471

[40] Ratajczak-Wielgomas K, Dziegiel P. The role of periostin in neoplastic processes. Folia Histochem Cytobiol. 2015;53:120–32. 26150285 10.5603/FHC.a2015.0014

[41] Mikheev AM, Mikheeva SA, Trister AD, Tokita MJ, Emerson SN, Parada CA, Born DE, Carnemolla B, Frankel S, Kim DH, et al. Periostin is a novel therapeutic target that predicts and regulates glioma malignancy. Neuro Oncol. 2015;17:372–82. 25140038 10.1093/neuonc/nou161 PMC4483094

[42] Nasrolahi A, Azizidoost S, Radoszkiewicz K, Najafi S, Ghaedrahmati F, Anbiyaee O, Khoshnam SE, Farzaneh M, Uddin S. Signaling pathways governing glioma cancer stem cells behavior. Cell Signal. 2023;101:110493. 36228964 10.1016/j.cellsig.2022.110493

[43] Pang L, Dunterman M, Xuan W, Gonzalez A, Lin Y, Hsu WH, Khan F, Hagan RS, Muller WA, Heimberger AB, et al. Circadian regulator CLOCK promotes tumor angiogenesis in glioblastoma. Cell Rep. 2023;42:112127. 36795563 10.1016/j.celrep.2023.112127 PMC10423747

[44] Qian J, Luo F, Yang J, Liu J, Liu R, Wang L, Wang C, Deng Y, Lu Z, Wang Y, et al. TLR2 promotes Glioma Immune Evasion by downregulating MHC class II molecules in Microglia. Cancer Immunol Res. 2018;6:1220–33. 30131377 10.1158/2326-6066.CIR-18-0020

[45] Lee AH, Sun L, Mochizuki AY, Reynoso JG, Orpilla J, Chow F, Kienzler JC, Everson RG, Nathanson DA, Bensinger SJ, et al. Neoadjuvant PD-1 blockade induces T cell and cDC1 activation but fails to overcome the immunosuppressive tumor associated macrophages in recurrent glioblastoma. Nat Commun. 2021;12:6938. 34836966 10.1038/s41467-021-26940-2 PMC8626557

[46] Chen RQ, Liu F, Qiu XY, Chen XQ. The Prognostic and Therapeutic Value of PD-L1 in Glioma. Front Pharmacol. 2018;9:1503. 30687086 10.3389/fphar.2018.01503 PMC6333638

