# Supplementary figures and images for: The dual role of POSTN in maintaining glioblastoma stem cells and the immunosuppressive phenotype of microglia in glioblastoma
Source: J Exp Clin Cancer Res. 2024 Sep 4;43:252. doi: 10.1186/s13046-024-03175-9 (PMC11373117; doi:10.1186/s13046-024-03175-9)

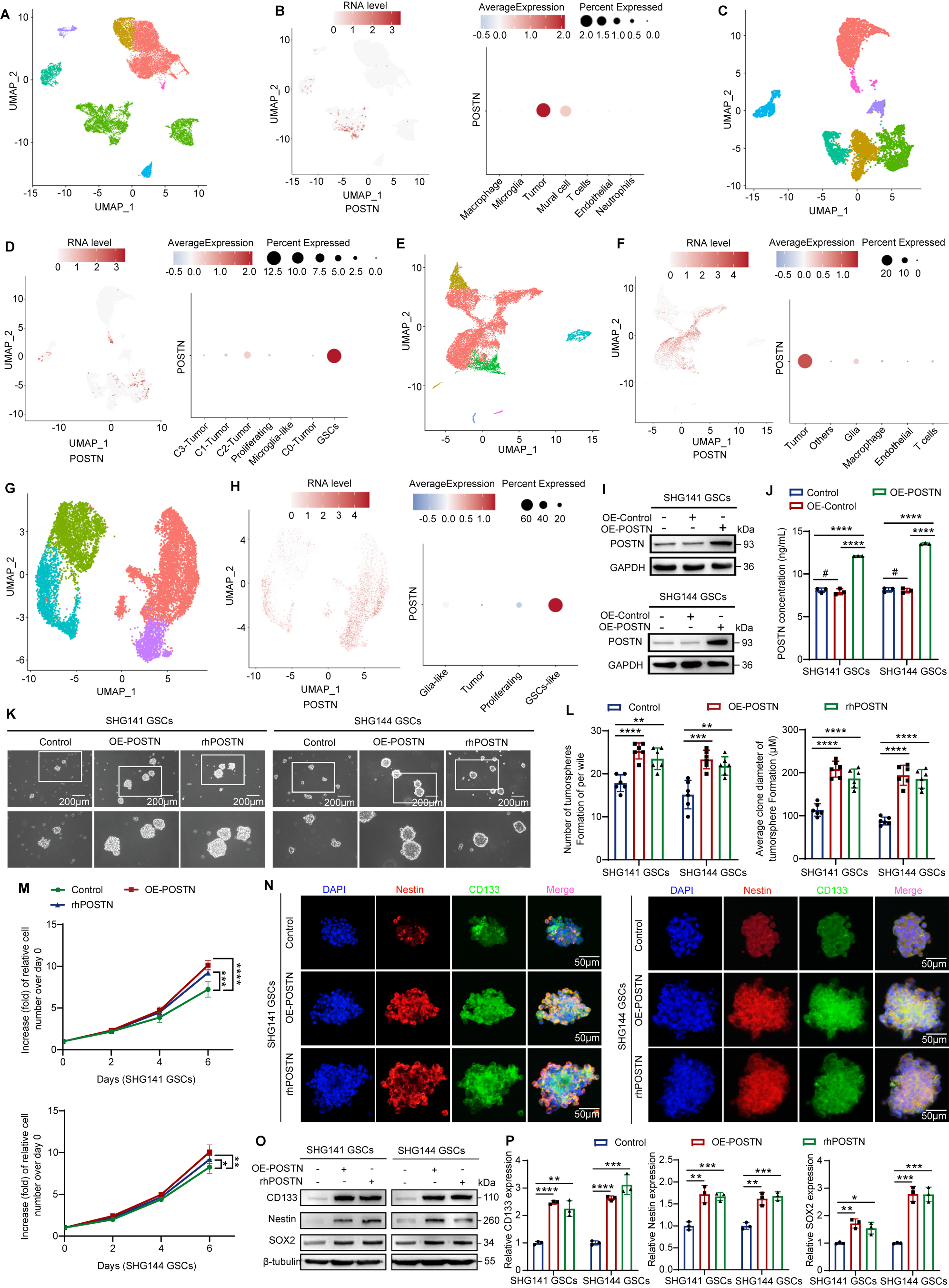

Supplement: Supplementary file 1 — Supplementary Material 1: Supplementary Fig. 1. POSTN overexpression promotes GSC self-renewal and proliferation. A, UMAP of single-cell RNA-seq data from GBM tissue cells (n = 19995). B, Expression of POSTN in UMAP (left) and different cell types (right). C, UMAP of single-cell RNA-seq data of tumor cells in GBM tissue. D, Expression of POSTN in UMAP (left) and different cell types (right). E, UMAP of the single-cell analysis dataset (GSE139448), cells = 12152. F, Expression of POSTN in UMAP (left) and different cell types (right). G, UMAP of tumor cells in the single-cell analysis dataset (GSE139448). H, Expression of POSTN in UMAP (left) and different cell types (right). I, Immunoblot analysis of POSTN in lysates from SHG141 GSCs and SHG144 GSCs in the control, overexpression vector control (OE-Control) and OE-POSTN groups. J, ELISA for the detection of secreted POSTN in the culture supernatant of GSCs expressing control, OE-Control or OE-POSTN. n = 3 biological replicates. K, Representative images of tumorspheres formed by SHG141 GSCs and SHG144 GSCs in the control, OE-POSTN, or rhPOSTN (1 µg ml− 1, 48 h) groups. Scale bar, 200 μm. L, Quantification of the number and diameter of SHG141 GSC- and SHG144 GSC-derived tumorspheres treated with control, OE-POSTN, or rhPOSTN (1 µg ml− 1, 48 h). n = 6 biological replicates. M, CCK-8 assay of SHG141 GSCs and SHG144 GSCs in the control, OE-POSTN, and rhPOSTN (1 µg ml− 1, 48 h) groups. n = 6 biological replicates. N, Representative images of immunofluorescence staining for CD133 and Nestin in SHG141 GSC- and SHG144 GSC-derived tumorspheres treated with control, OE-POSTN, or rhPOSTN (1 µg ml− 1, 48 h). O and P, Immunoblot analysis of CD133, Nestin and SOX2 in SHG141 GSCs and SHG144 GSCs in the control, OE-POSTN, and rhPOSTN (1 µg ml− 1, 48 h) groups. CD133, Nestin and SOX2 protein levels were quantified (G). The error bars indicate the means ± SDs (J, L and P). Two-tailed Student’s t test (J, L, M and P). *p < 0.05, **p < 0 [file 13046_2024_3175_MOESM1_ESM.tif]

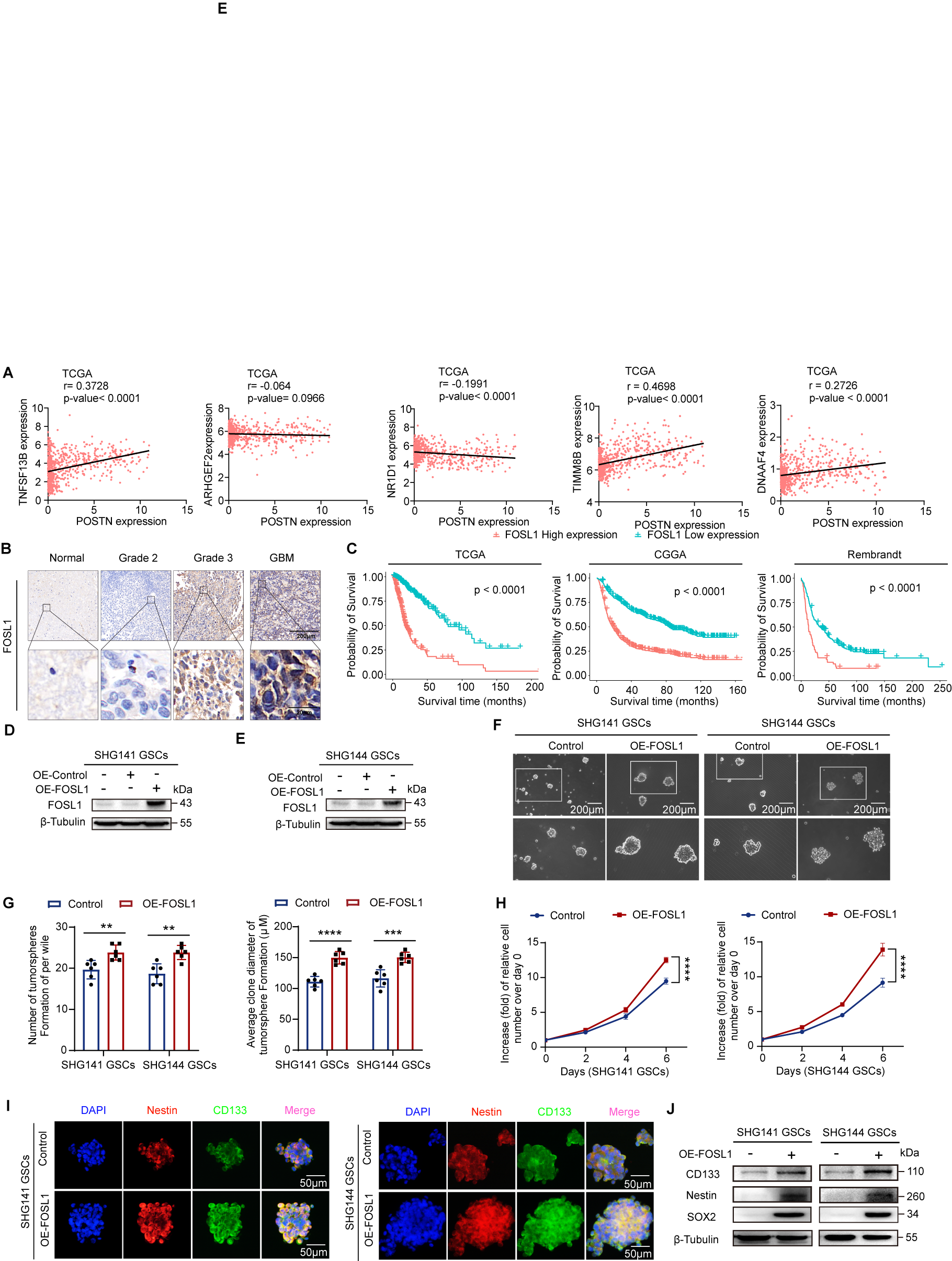

Supplement: Supplementary file 2 — Supplementary Material 2: Supplementary Fig. 2. FOSL1 overexpression promotes GSC self-renewal and proliferation. A, Correlations between the expression of POSTN and the expression of TNFSF13B, ARHGEF2, NR1D1, TIMM8B, and DNAAF4 in the TCGA dataset; n = 675. B, Images showing the expression of FOSL1 in human normal brain tissue and glioma tissue samples via immunohistochemical staining. Scale bar, 200 μm (top), 20 μm (bottom). C, Kaplan–Meier survival analysis of patients stratified by the median FOSL1 level in different glioma datasets: the TCGA, CGGA, and Rembrandt datasets. TCGA datasets: low POSTN expression, n = 258; high POSTN expression, n = 354. CGGA datasets: low POSTN expression, n = 419; high POSTN expression, n = 551. Rembrandt datasets: low POSTN expression, n = 228; high POSTN expression, n = 71. D and E, Immunoblot analysis of FOSL1 in lysates from SHG141 GSCs and SHG144 GSCs in the control, OE-Control and OE-FOSL1 groups. F, Representative images of tumorspheres formed by SHG141 GSCs and SHG144 GSCs in the control and OE-FOSL1 groups. Scale bar, 200 μm. G, Quantification of the number and diameter of SHG141 GSC-derived and SHG144 GSC-derived tumorspheres in the control and OE-FOSL1 groups. n = 6 biological replicates. H, CCK-8 assay of SHG141 GSCs and SHG144 GSCs in the control and OE-FOSL1 groups. n = 6 biological replicates. I, Representative images of immunofluorescence staining for CD133 and Nestin in SHG141 GSC- and SHG144 GSC-derived tumorspheres in the control and OE-FOSL1 groups. J, Immunoblot analysis of CD133, Nestin and SOX2 in SHG141 GSCs and SHG144 GSCs in the control and OE-FOSL1 groups. [file 13046_2024_3175_MOESM2_ESM.tif]

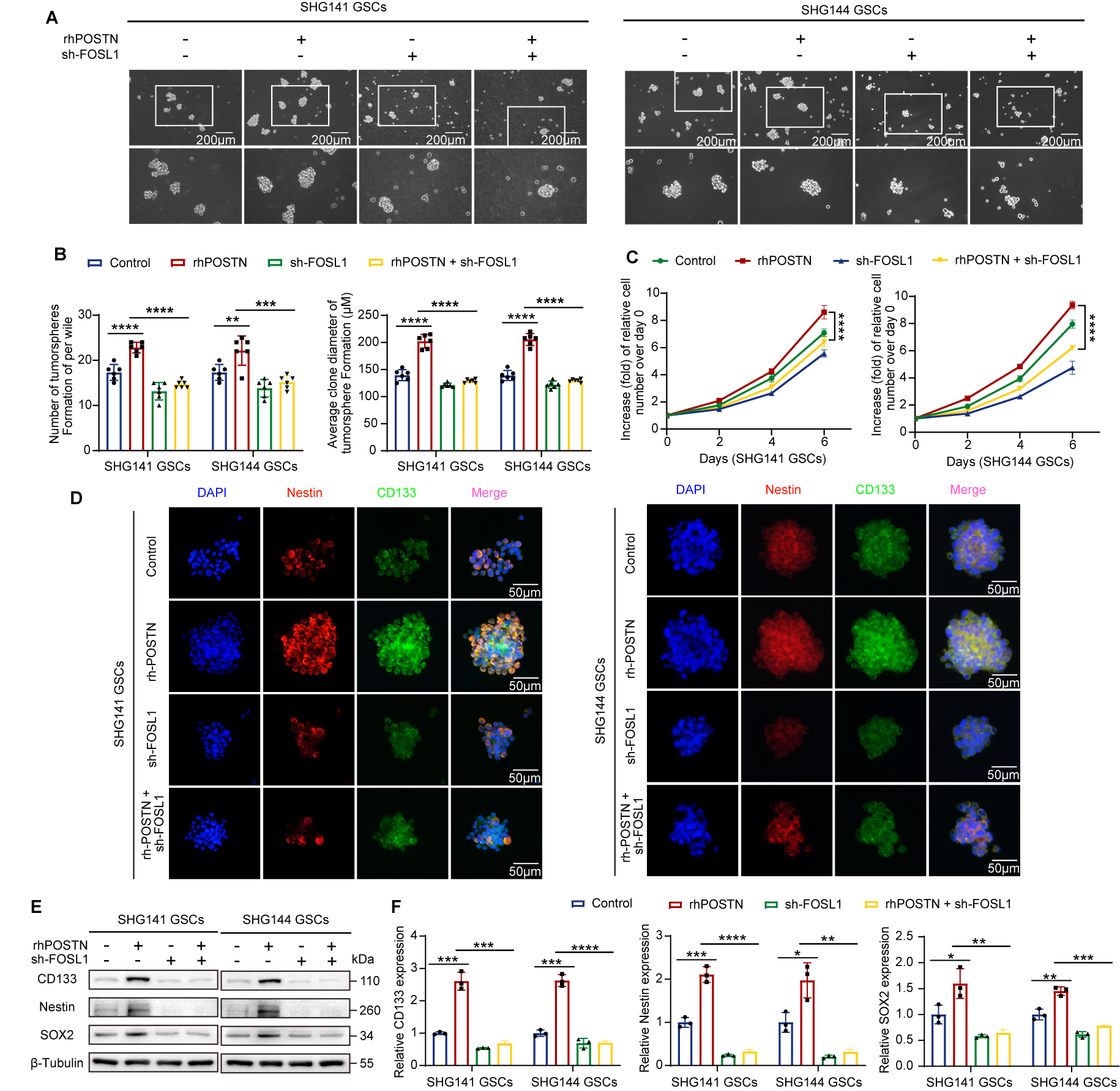

Supplement: Supplementary file 3 — Supplementary Material 3: Supplementary Fig. 3. FOSL1 is a crucial factor that mediates the ability of POSTN to promote GSC self-renewal. A, Representative images of tumorspheres formed by SHG141 GSCs and SHG144 GSCs expressing control or sh-FOSL1 and treated with or without rhPOSTN (1 µg ml− 1) for 48 h. Scale bar, 200 μm. B, Quantification of the number and diameter of SHG141 GSC- and SHG144 GSC-derived tumorspheres expressing control or sh-FOSL1 and treated with or without rhPOSTN (1 µg ml− 1) for 48 h; n = 6 biological replicates. C, CCK-8 assay of SHG141 GSCs and SHG144 GSCs expressing control or sh-FOSL1 and treated with or without rhPOSTN (1 µg ml− 1) for 48 h; n = 6 biological replicates. D, Representative images of immunofluorescence staining of CD133 and Nestin in SHG141 GSCs and SHG144 GSCs expressing control or sh-FOSL1 and treated with or without rhPOSTN (1 µg ml− 1) for 48 h. Scale bar, 50 μm. E and F, Immunoblot analysis of CD133, Nestin and SOX2 in SHG141 GSCs and SHG144 GSCs expressing control or sh-FOSL1 and treated with or without rhPOSTN (1 µg ml− 1) for 48 h. CD133, Nestin and SOX2 protein levels were quantified (F). The error bars indicate the means ± SDs (B, C, and F). Two-tailed Student’s t test (B, C, and F). *p < 0.05, **p < 0.01, ***p < 0.001, and ****p < 0.0001. [file 13046_2024_3175_MOESM3_ESM.tif]

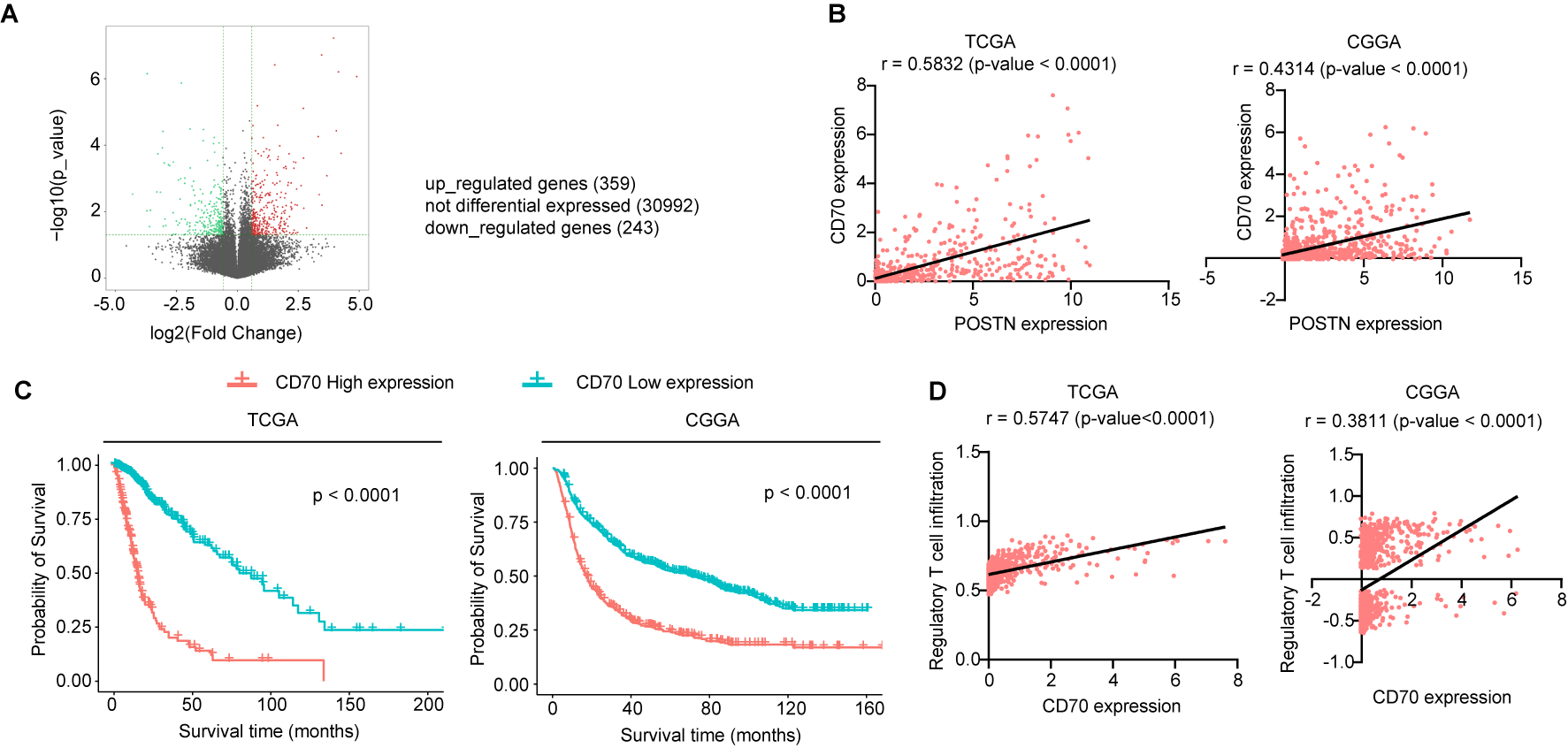

Supplement: Supplementary file 4 — Supplementary Material 4: Supplementary Fig. 4. CD70 is highly expressed in GBM and is associated with poor outcomes in glioma patients. A, Volcano plot displaying the differentially expressed genes identified via RNA-seq analysis of HMC3 cells treated with or without rhPOSTN (1 µg ml− 1) for 48 h. B, Correlations between POSTN and CD70 expression in the TCGA and CGGA datasets. TCGA datasets: n = 675. CGGA datasets: n = 1018. R and P values were determined by Pearson correlation analysis. C, Kaplan–Meier survival analysis of patients stratified by CD70 expression in different glioma datasets: the TCGA, CGGA, and Rembrandt datasets. TCGA datasets: low POSTN expression, n = 308; high POSTN expression, n = 307. CGGA datasets: low POSTN expression, n = 485; high POSTN expression, n = 485. D, Correlation between CD70 expression and regulatory T-cell infiltration in the TCGA and CGGA datasets. TCGA datasets: n = 675. CGGA datasets: n = 1018. R and P values were determined by Pearson correlation analysis. [file 13046_2024_3175_MOESM4_ESM.tif]

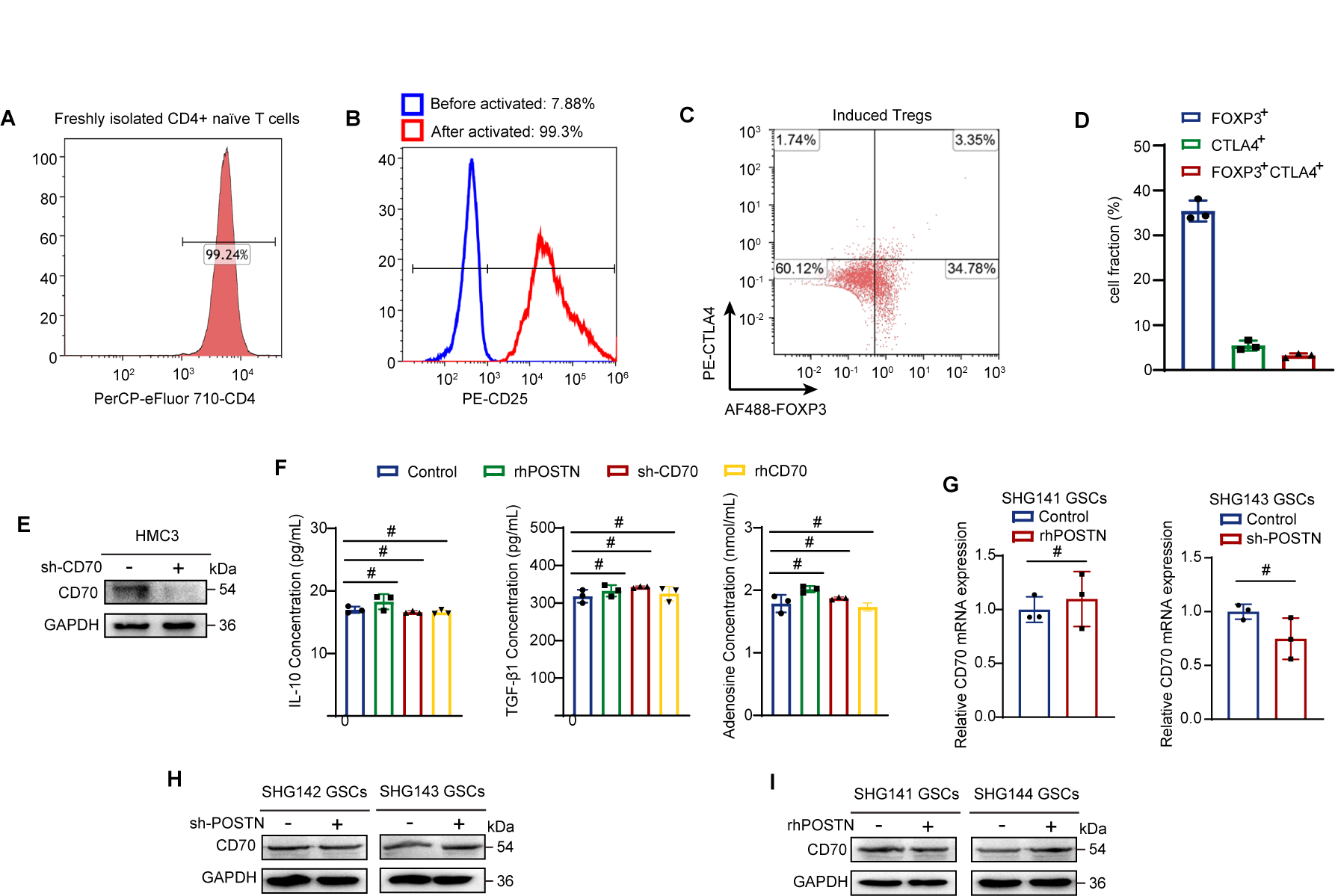

Supplement: Supplementary file 5 — Supplementary Material 5: Supplementary Fig. 5. Treating microglia with rhPOSTN promotes treg development and function. A, Immunophenotyping of freshly isolated naïve CD4+ T cells according to CD4 expression. B, Immunophenotyping of TCR-activated naïve CD4+ T cells according to CD25 expression. C, Immunophenotyping of naïve CD4+ T cells after 3 days of induced Treg differentiation without HMC3 cell coculture. D, Proportions of FOXP3+ Tregs and FOXP3+CTLA4+ activated Tregs; n = 3. E, Immunoblots showing CD70 expression in HMC3 cells expressing control or sh-POSTN. F, Changes in the levels of the immunosuppressive factors IL-10, TGF-β1, and adenosine in HMC3 cells treated with the control, rhPOSTN (1 µg ml− 1), sh-CD70 or rhCD70 (800 ng ml− 1) for 48 h. IL-10 and TGF-β1 were measured by ELISA, and adenosine was measured via a fluorescent adenosine assay, n = 3 G, RNA-seq analysis of CD70 in SHG143 GSCs expressing control or sh-POSTN and in SHG141 GSCs treated with control or rhPOSTN (1 µg ml− 1) for 48 h. H, Immunoblots showing CD70 expression in SHG142 GSCs and SHG143 GSCs expressing control or sh-POSTN. I, Immunoblots showing CD70 expression in SHG141 GSCs and SHG144 GSCs treated with control or rhPOSTN (1 µg ml− 1) for 48 h. The error bars indicate the means ± SDs (D, F and G). Two-tailed Student’s t test (F and G). #, nonsignificant; **p < 0.01, ***p < 0.001, and ****p < 0.0001. [file 13046_2024_3175_MOESM5_ESM.tif]

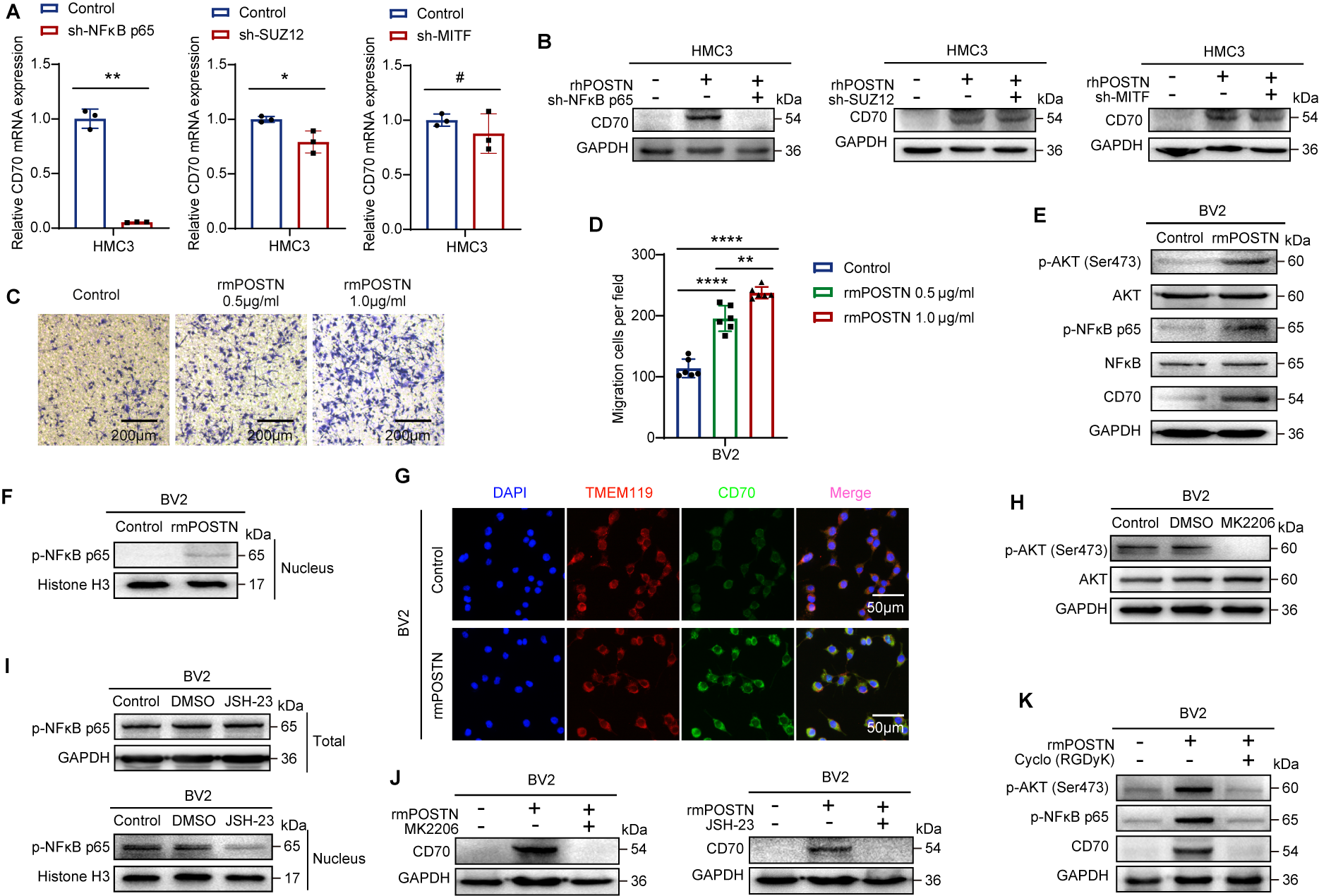

Supplement: Supplementary file 6 — Supplementary Material 6: Supplementary Fig. 6. CD70 expression is regulated by NFκB in the POSTN/αvβ3/PI3K/AKT pathway in BV2 cells. A, qRT‒PCR analysis of CD70 mRNA expression in HMC3 cells expressing control, sh-NFκB p65, sh-SUZ12, or sh-MITF; n = 3 for all groups. B, Immunoblots showing CD70 expression in HMC3 cells treated with control, rhPOSTN (1 µg ml− 1) or rhPOSTN (1 µg ml− 1) + sh-NFκB p65/sh-SUZ12/sh-MITF for 48 h. C and D, Representative images (C) and quantitative analysis (D) of the relative migration of BV2 cells following stimulation with recombinant mouse POSTN (rmPOSTN); n = 6. Scale bars, 200 μm. E, Immunoblots showing the p-AKT (Ser473), AKT, p-NFκB p65, NFκB p65 and CD70 levels in BV2 cells treated with or without rmPOSTN (1 µg ml− 1) for 48 h. F, Nuclear lysate was used to determine the p-NFκB p65 level in BV2 cells treated with or without rmPOSTN (1 µg ml− 1) for 48 h. G, Representative images of immunofluorescence staining of CD70 in BV2 cells treated with control or rmPOSTN (1 µg ml− 1, 48 h). Scale bar, 50 μm. H, Immunoblot analysis of р-AKT (Ser473) and AKT in BV2 cells treated with the control, DMSO or MK2206 (10 µM) for 48 h. I, Total lysate and the nuclear fraction were used to measure p-NFκB p65 levels in BV2 cells after treatment with JSH-23 (10 µM) for 48 h. J, Immunoblots showing CD70 expression in BV2 cells treated with control, rmPOSTN (1 µg ml− 1) or rmPOSTN (1 µg ml− 1) + MK2206 (10 µM)/JSH-23 (10 µM) for 48 h. K, Immunoblot analysis of p-AKT (Ser473), p-NFκB p65, and CD70 in BV2 cells treated with control, rmPOSTN (1 µg ml− 1) or rmPOSTN (1 µg ml− 1) + Cyclo(RGDyK) (100 nM) for 48 h. The error bars indicate the means ± SDs (A, and D). Two-tailed Student’s t test (A, and D). #, nonsignificant; *p < 0.05, **p < 0.01, and ****p < 0.0001. [file 13046_2024_3175_MOESM6_ESM.tif]

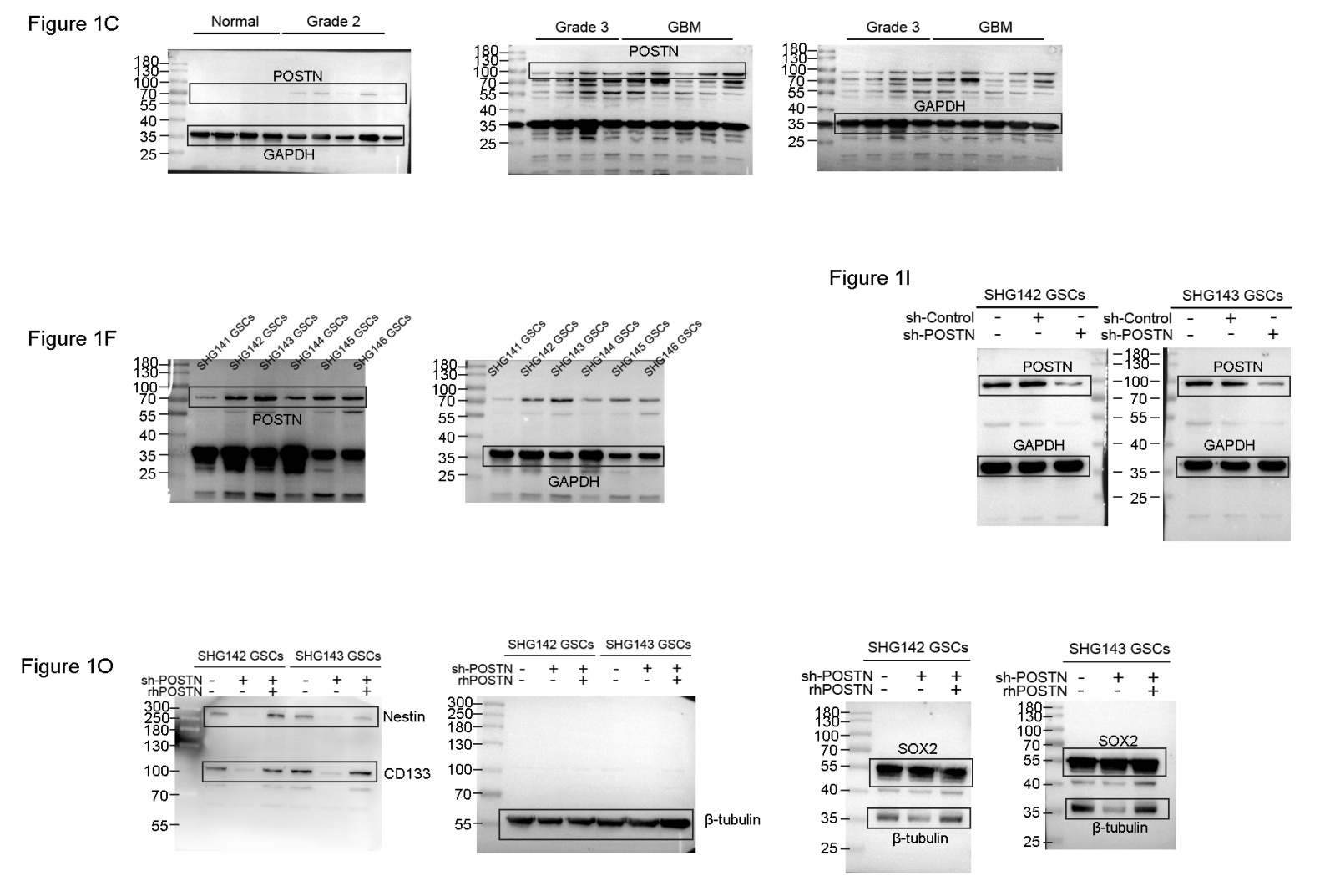


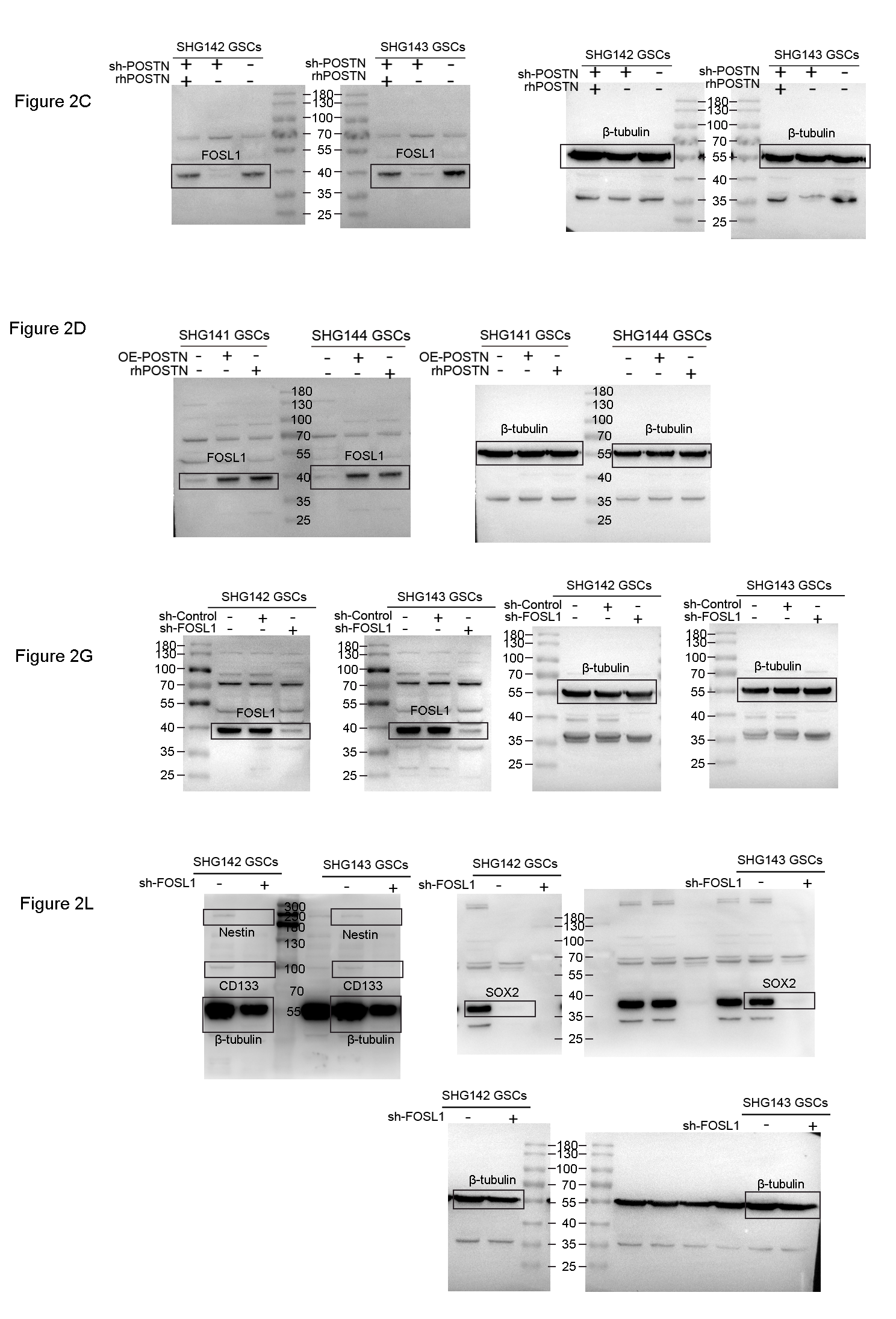


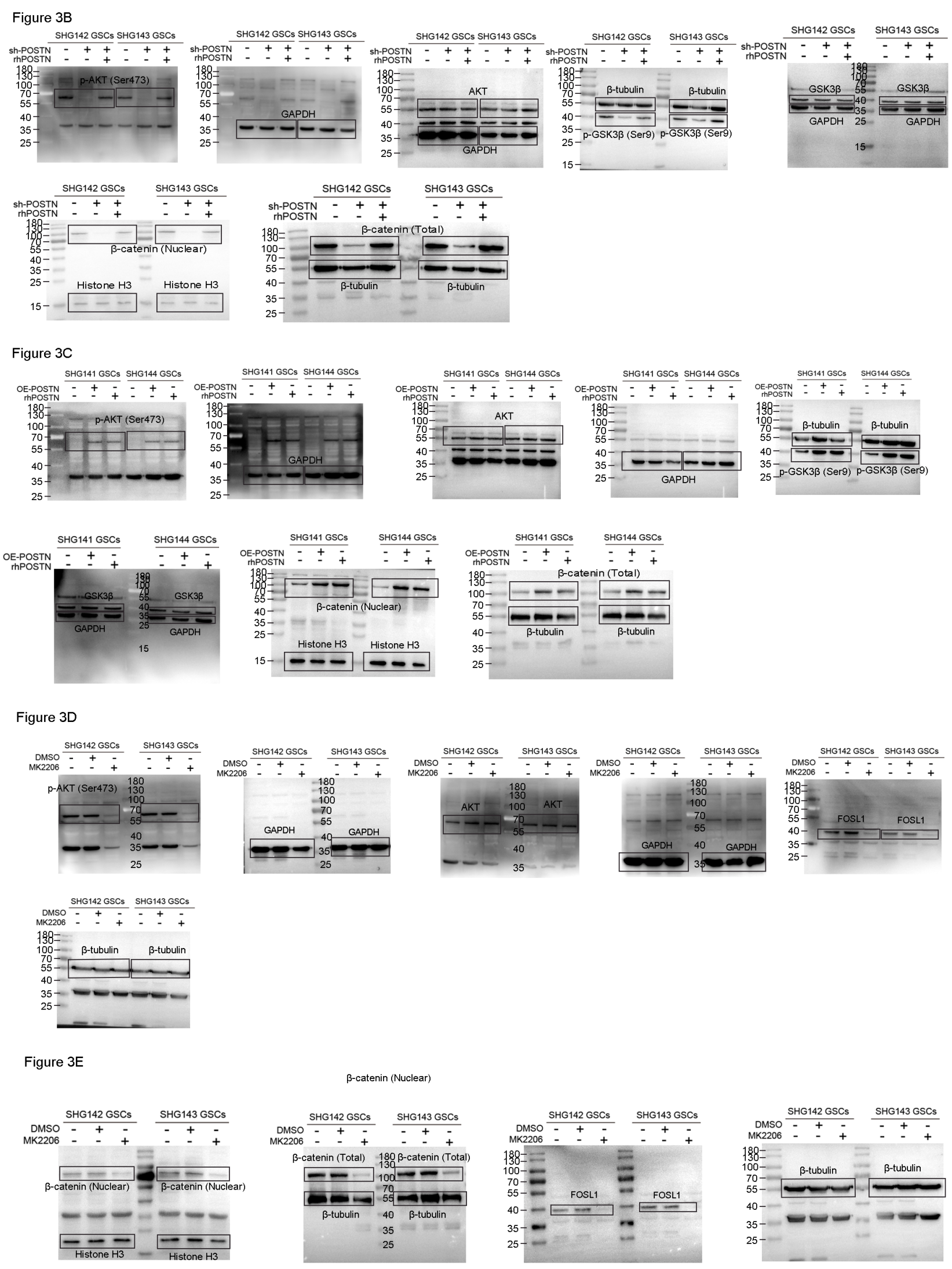


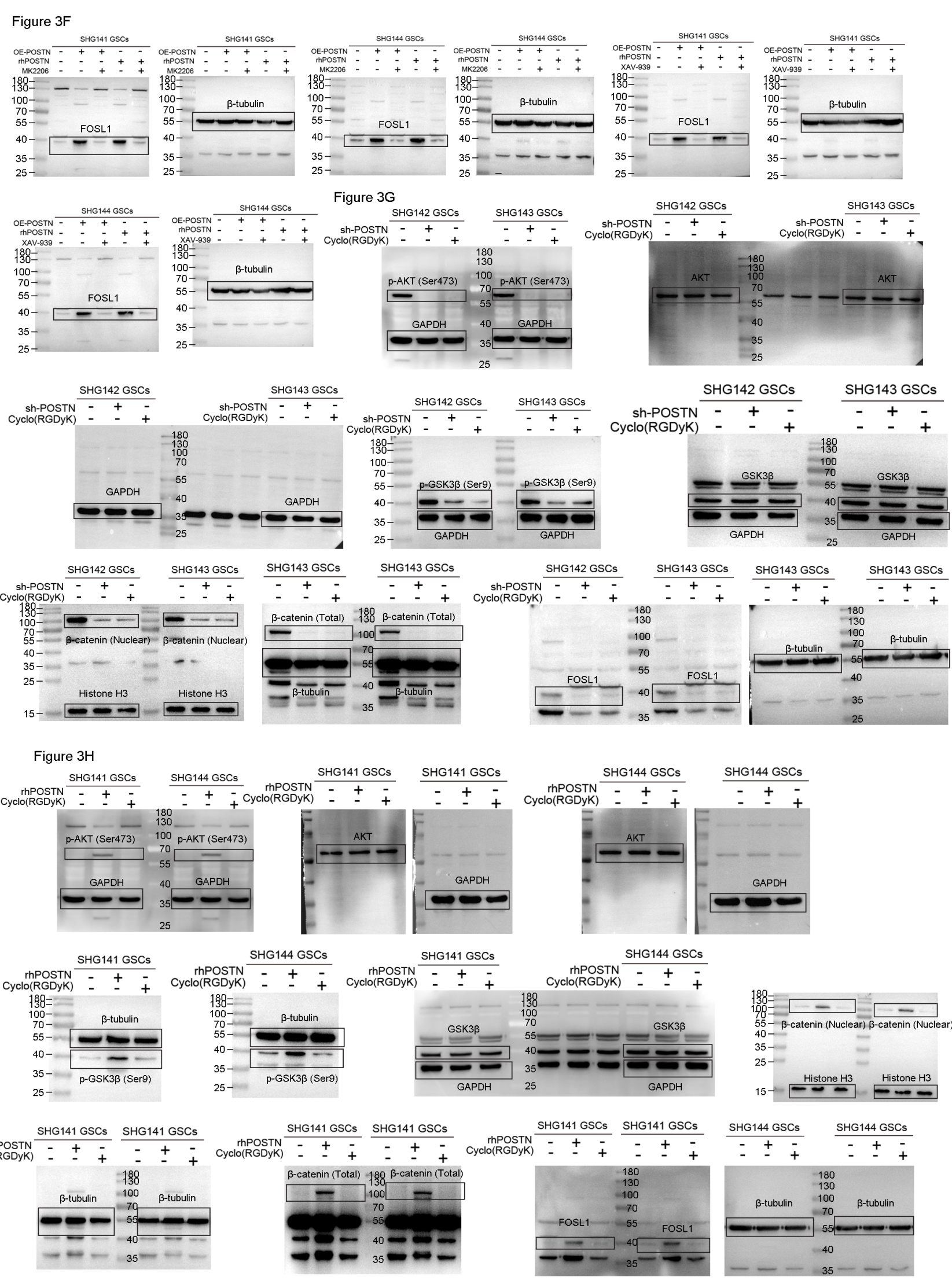


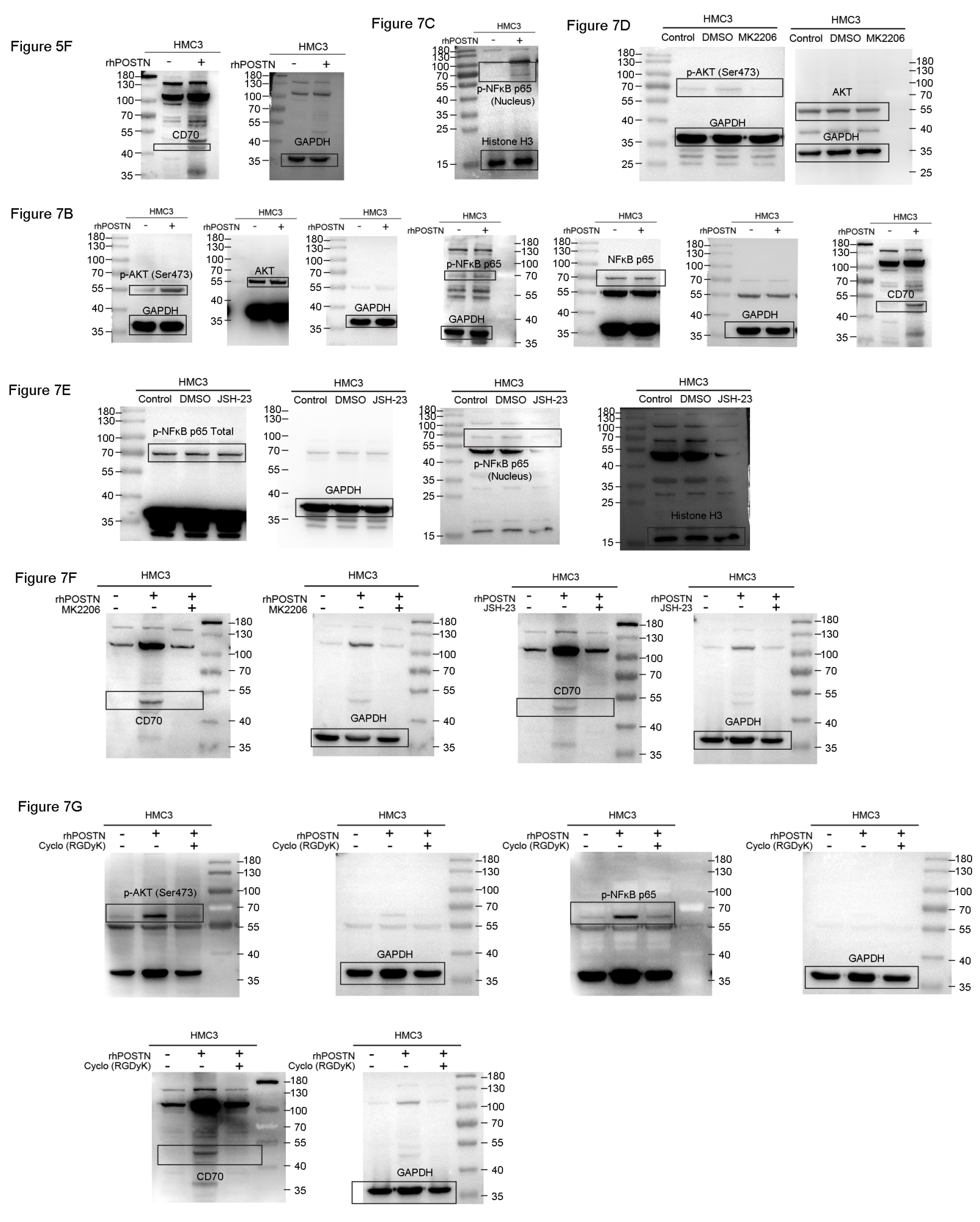


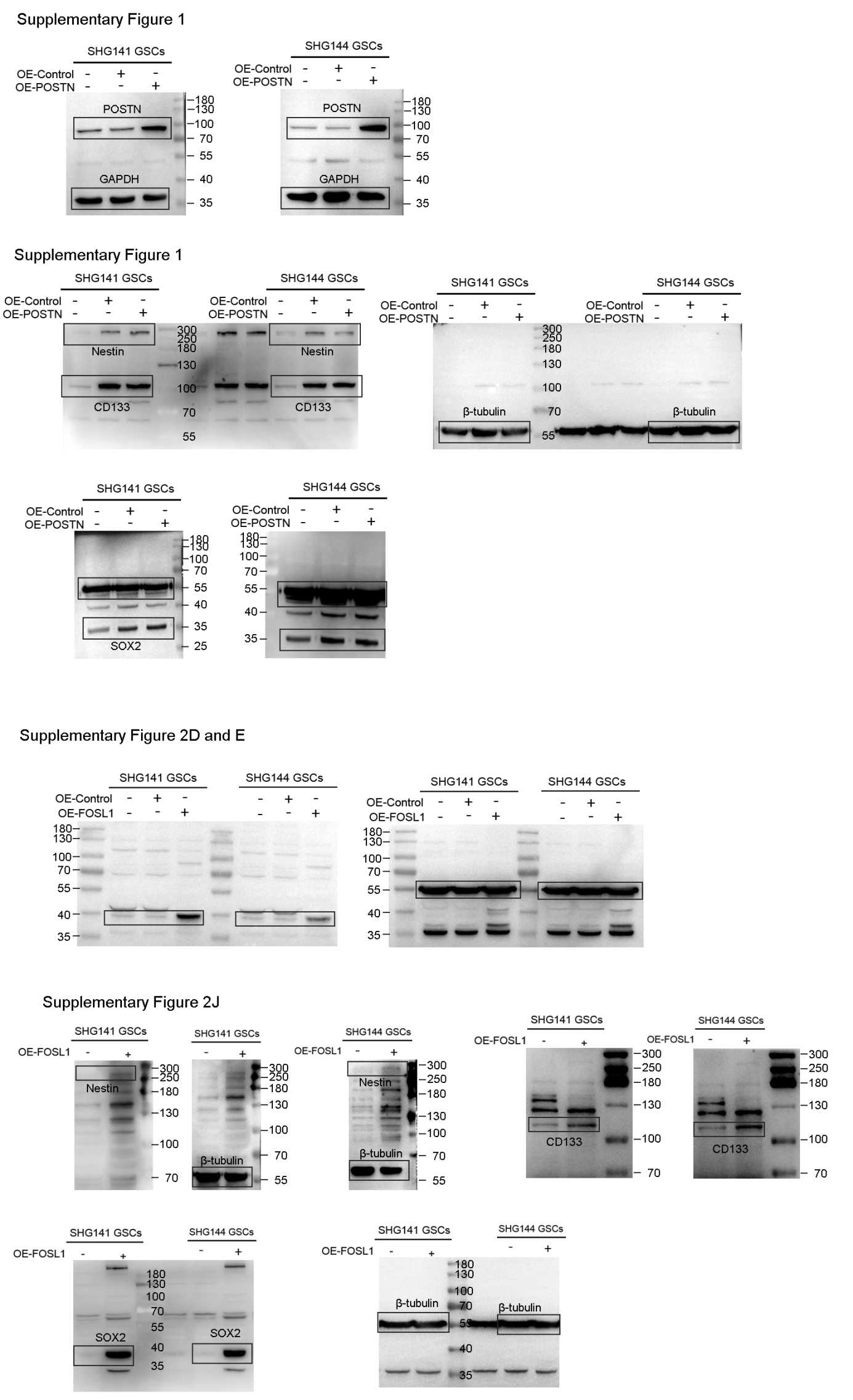


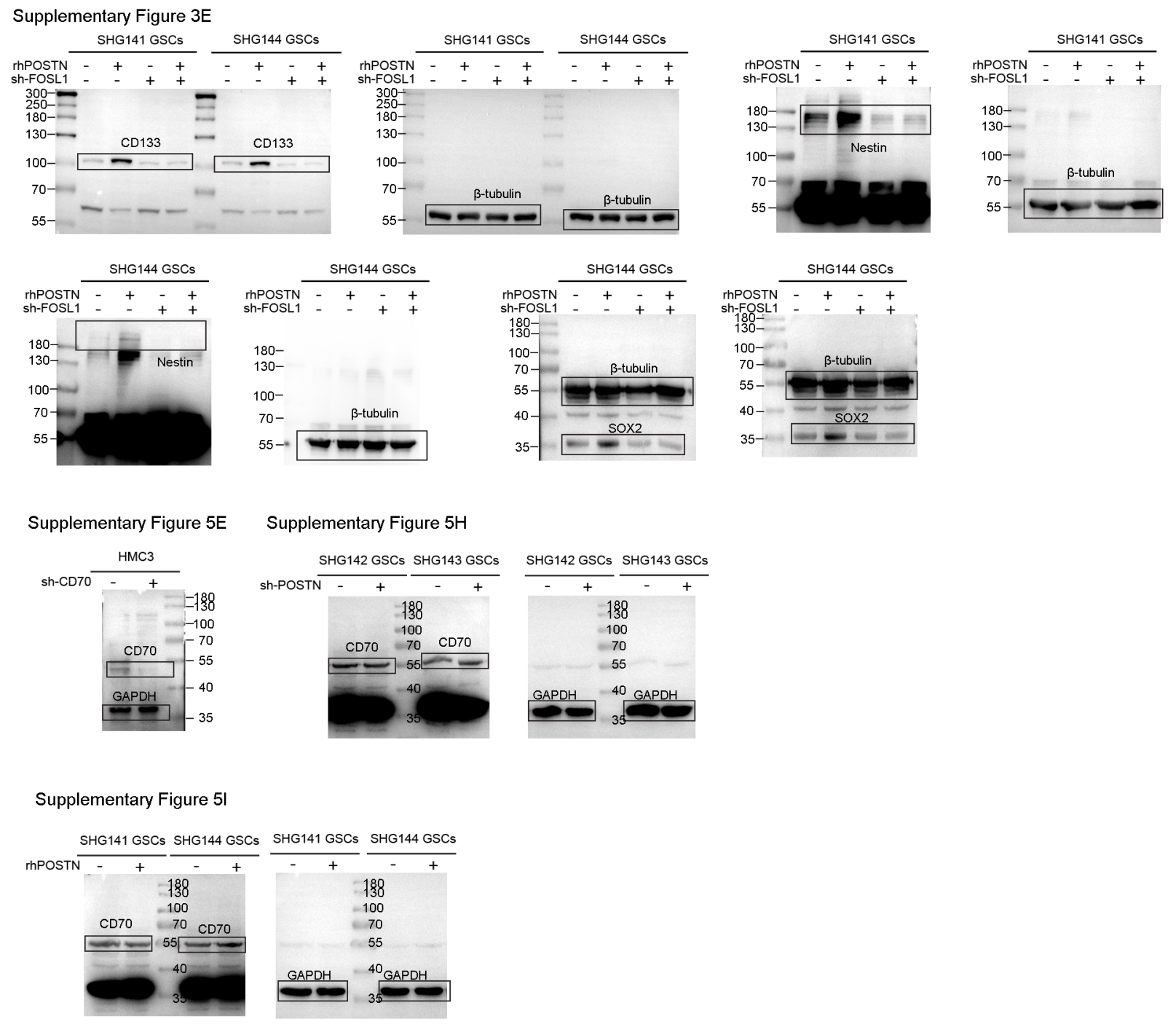


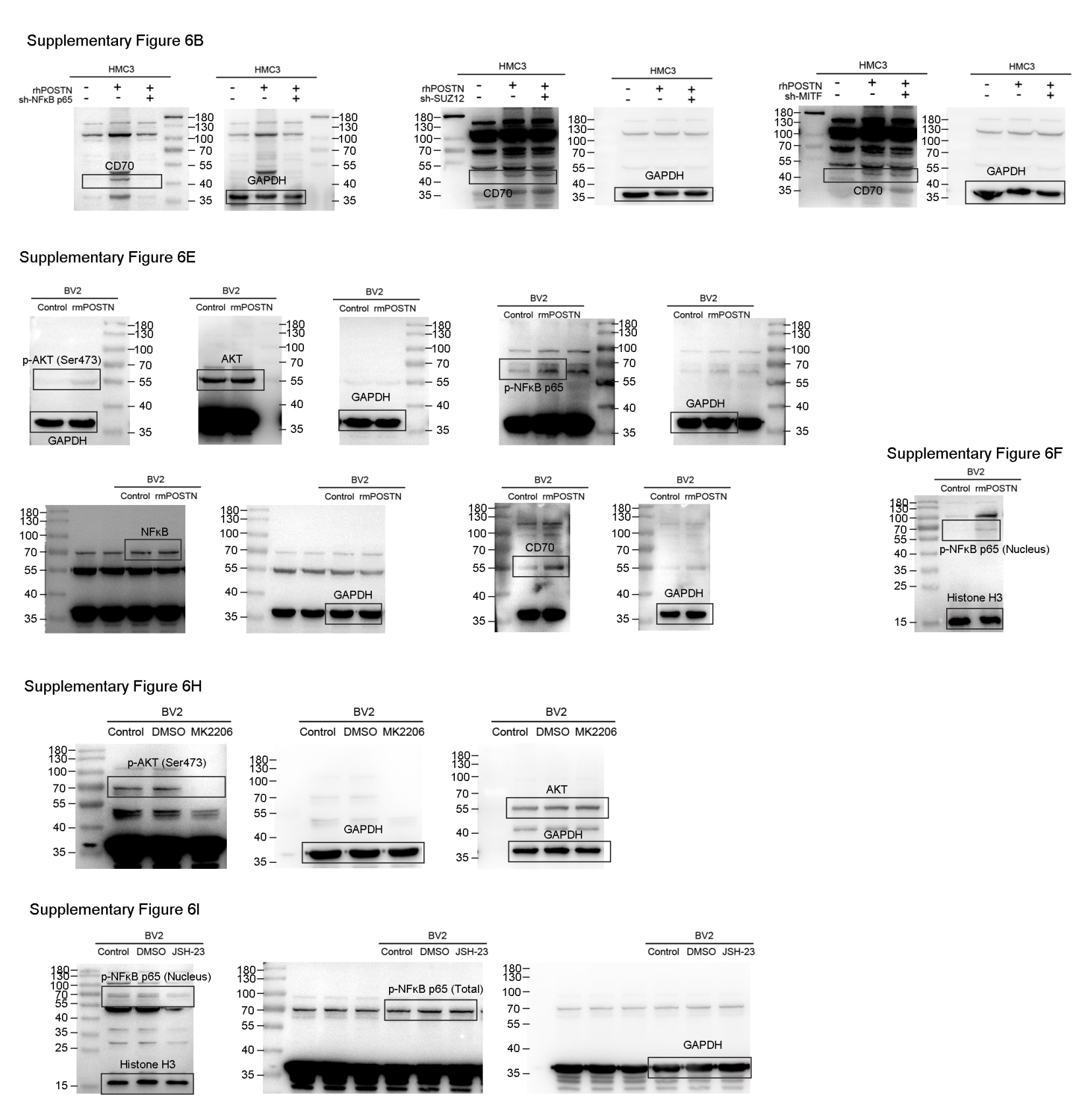


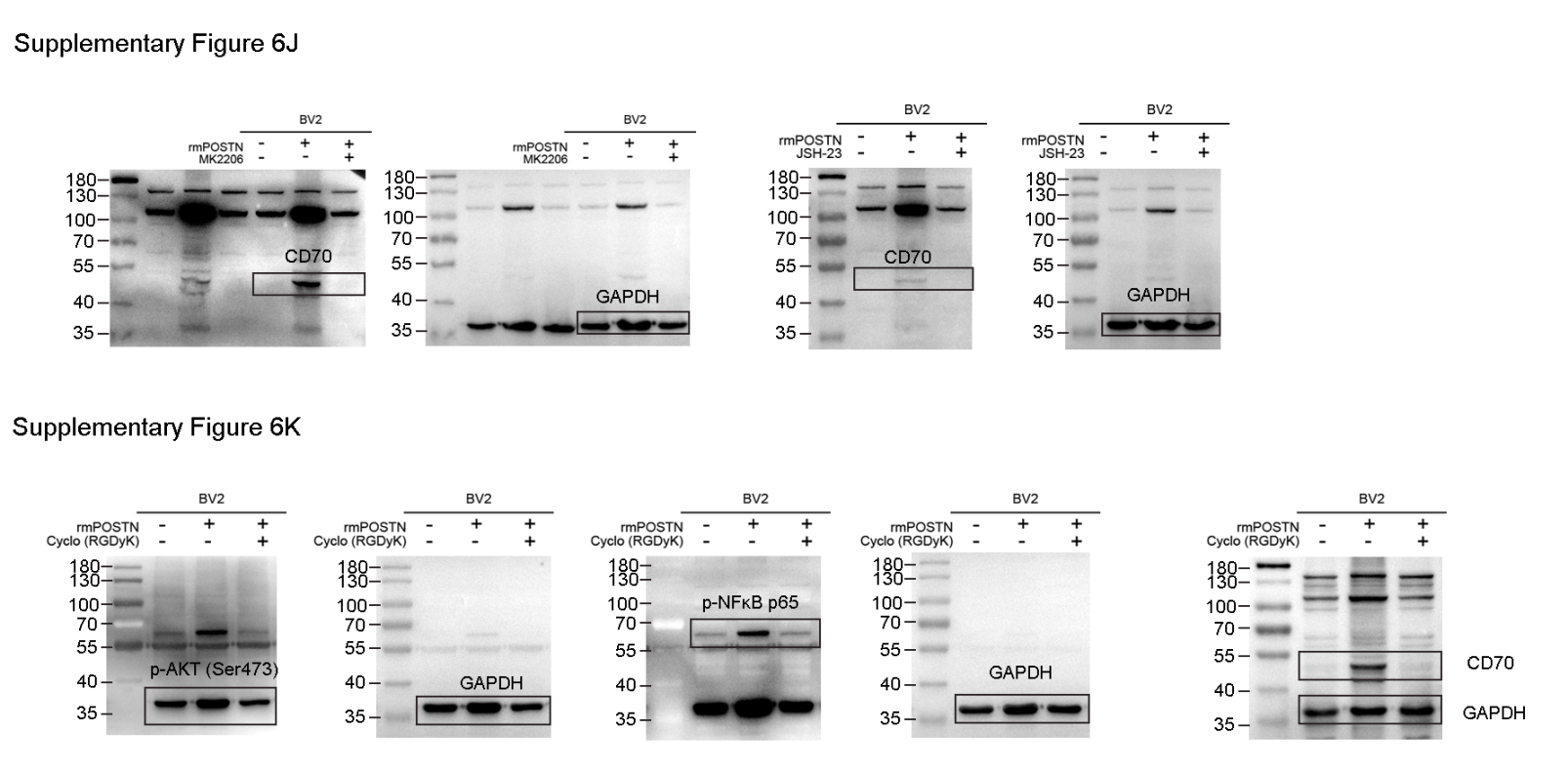

Supplement: Supplementary file 8 — Supplementary Material 8 [file 13046_2024_3175_MOESM8_ESM.docx]
