# Supplementary material for: The dual role of POSTN in maintaining glioblastoma stem cells and the immunosuppressive phenotype of microglia in glioblastoma
Source: J Exp Clin Cancer Res. 2024 Sep 4;43:252. doi: 10.1186/s13046-024-03175-9 (PMC11373117; doi:10.1186/s13046-024-03175-9)
Supplement: Supplementary file 7 — Supplementary Material 7 [file 13046_2024_3175_MOESM7_ESM.docx]

**Supplementary Table S1: The clinical information of the analyzed glioma cells.**

| **GSC cell** | **Histology** | **Tissue source site** | **Gender** | **Age** |
| --- | --- | --- | --- | --- |
| SHG141 GSCs | Glioblastoma | Right frontal lobe | Male | 42 |
| SHG142 GSCs | Glioblastoma | Right temporal lobe | Female | 70 |
| SHG143 GSCs | Glioblastoma | Left frontotemporal lobe | Male | 61 |
| SHG144 GSCs | Glioblastoma | Left temporoparietal lobe | Female | 52 |
| SHG145 GSCs | Glioblastoma | Left temporal lobe | Female | 65 |
| SHG146 GSCs | Glioblastoma | Right temporooccipital lobe | Male | 66 |

**Supplementary Table S2: Antibody information.**

| **Primary antibody** | **Specificity** | **Usage in this study (dilution)** | **Citation** | **SOURCE** |
| --- | --- | --- | --- | --- |
| Rabbit-anti-POSTN | Human, Mouse | IB (1:1000),  IHC (1:200) | ab92460 | Abcam |
| Rabbit-anti-CD133 | Human | IB (1:1000), IHC (1:200), IF (1:200) | ab19898 | Abcam |
| Mouse-anti-Nestin | Human | IB (1:1000), IHC (1:400), IF (1:2000) | 33475S | Cell Signalling Technology |
| Rabbit-anti-SOX2 | Human | IB (1:1000) | ab97959 | Abcam |
| Rabbit-anti-FOSL1 | Human | IB (1:1000),  IHC (1:200) | DF3096 | Affinity Biosciences |
| Rabbit-anti-AKT | Human | IB (1:1000) | 9272S | Cell Signalling Technology |
| Rabbit-anti-p-AKT (Ser473) | Human | IB (1:1000) | 4060S | Cell Signalling Technology |
| Rabbit-anti-GSK3β | Human | IB (1:1000) | 12456S | Cell Signalling Technology |
| Rabbit-anti-p-GSK3β (Ser9) | Human | IB (1:1000) | 5558S | Cell Signalling Technology |
| β-catenin | Human | IB (1:1000), IHC (1:400), ChIP (1 μg for each sample) | 8480S | Cell Signalling Technology |
| Rabbit-anti-Histone H3 | Human | IB (1:2000) | A2348 | ABclonal |
| Rabbit-anti-CD70 | Human, Mouse | IB (1:1000),  IF (1:100) | A2032 | ABclonal |
| Mouse-anti-TMEM119 | Human | IF (1:200) | MA5-  35043 | Thermo Fisher Scientific |
| Mouse-anti-TMEM119 | Mouse | IF (1:200) | 98778S | Cell Signalling Technology |
| Rabbit-anti-NFκB p65 | Human | IB (1:1000), ChIP (1 μg for each sample) | 8242S | Cell Signalling Technology |
| Rabbit-anti-p-NFκB p65 | Human, Mouse | IB (1:1000),  IHC (1:400) | AP0475 | ABclonal |
| GAPDH | Human, Mouse | IB (1:1000) | 2118S | Cell Signalling Technology |
| β-Tubulin | Human, Mouse | IB (1:1000) | 2146S | Cell Signalling Technology |
| Normal Rabbit IgG | Human | ChIP (1 μg for each sample) | 2729S | Cell Signalling Technology |

**Supplementary Table S3: Genes correlated with POSTN in the TCGA database**

| **Gene** | **cor** | **pvalue** | **Gene** | **cor** | **pvalue** |
| --- | --- | --- | --- | --- | --- |
| ADAM12 | 0.808 | 1.18E-162 | GAPT | 0.549 | 2.23E-56 |
| TIMP1 | 0.807 | 4.59E-162 | CD70 | 0.549 | 2.27E-56 |
| COL5A2 | 0.791 | 2.14E-151 | SNAPC2 | 0.549 | 2.28E-56 |
| SMAGP | 0.791 | 3.00E-151 | AC079944.1 | 0.549 | 2.33E-56 |
| GPX8 | 0.789 | 4.57E-150 | BCAP31 | 0.549 | 2.39E-56 |
| COL5A1 | 0.788 | 1.87E-149 | TRIM21 | 0.549 | 2.41E-56 |
| KDELR3 | 0.788 | 3.39E-149 | NIP7 | 0.548 | 2.61E-56 |
| SPRY1 | 0.786 | 5.81E-148 | SUMO1P3 | 0.548 | 2.71E-56 |
| PHETA2 | 0.780 | 1.07E-144 | CST7 | 0.548 | 2.72E-56 |
| GDF15 | 0.775 | 3.24E-141 | GBP1 | 0.548 | 2.80E-56 |
| IGFBP2 | 0.773 | 2.26E-140 | AC131235.1 | 0.548 | 3.11E-56 |
| TNFAIP6 | 0.772 | 2.05E-139 | BIRC5 | 0.548 | 3.23E-56 |
| ANXA2P2 | 0.770 | 1.97E-138 | SPCS3 | 0.548 | 3.34E-56 |
| TAGLN2 | 0.769 | 8.90E-138 | SOCS2 | 0.548 | 3.40E-56 |
| SERPINH1 | 0.766 | 3.61E-136 | MRPL22 | 0.548 | 3.45E-56 |
| RAB34 | 0.760 | 4.99E-133 | PEBP1P2 | 0.548 | 3.45E-56 |
| COL3A1 | 0.760 | 1.00E-132 | SOD2 | 0.548 | 3.60E-56 |
| SMIM3 | 0.760 | 1.08E-132 | ATP6V0B | 0.548 | 4.15E-56 |
| AC083855.2 | 0.758 | 4.18E-132 | OIP5 | 0.548 | 4.39E-56 |
| SRPX2 | 0.758 | 4.51E-132 | PDGFRL | 0.548 | 4.41E-56 |
| EMP3 | 0.758 | 4.80E-132 | HOXC6 | 0.547 | 4.96E-56 |
| COL6A2 | 0.756 | 6.60E-131 | TLNRD1 | 0.547 | 5.00E-56 |
| PCOLCE | 0.753 | 6.10E-129 | IFT22 | 0.547 | 5.19E-56 |
| COL1A2 | 0.752 | 1.01E-128 | UACA | 0.547 | 5.21E-56 |
| AC064875.1 | 0.752 | 1.96E-128 | ADAM9 | 0.547 | 5.27E-56 |
| PTX3 | 0.752 | 2.17E-128 | GZMA | 0.547 | 5.31E-56 |
| CLIC1 | 0.751 | 2.25E-128 | PALLD | 0.547 | 5.36E-56 |
| LAMC1 | 0.751 | 5.56E-128 | SLAMF9 | 0.547 | 5.36E-56 |
| CTHRC1 | 0.750 | 1.78E-127 | UNC45A | 0.547 | 5.54E-56 |
| ARSJ | 0.748 | 8.34E-127 | AC078819.1 | 0.547 | 5.79E-56 |
| LOXL1 | 0.748 | 1.91E-126 | PRXL2C | 0.547 | 6.08E-56 |
| ANXA2 | 0.746 | 1.10E-125 | AGAP2-AS1 | 0.547 | 6.22E-56 |
| FKBP9 | 0.746 | 2.35E-125 | FTH1P7 | 0.547 | 6.69E-56 |
| CD248 | 0.744 | 2.64E-124 | CCL20 | 0.547 | 6.80E-56 |
| LUM | 0.744 | 2.82E-124 | AC092718.4 | 0.547 | 6.82E-56 |
| NTAN1 | 0.743 | 3.68E-124 | AL117332.1 | 0.547 | 6.82E-56 |
| COL1A1 | 0.743 | 3.86E-124 | MYBL2 | 0.547 | 7.52E-56 |
| CHRNA9 | 0.743 | 4.68E-124 | AC004453.1 | 0.547 | 7.68E-56 |
| LGALS3 | 0.743 | 5.20E-124 | NAT1 | 0.547 | 7.74E-56 |
| TPM4 | 0.743 | 6.05E-124 | PIGB | 0.546 | 7.81E-56 |
| MYL12A | 0.743 | 7.80E-124 | ECT2 | 0.546 | 8.00E-56 |
| EFEMP2 | 0.743 | 8.51E-124 | TECRP1 | 0.546 | 8.15E-56 |
| TAGLN2P1 | 0.741 | 3.76E-123 | ACAA2 | 0.546 | 8.19E-56 |
| OCIAD2 | 0.740 | 1.31E-122 | EEF1A1P8 | 0.546 | 8.86E-56 |
| MCUB | 0.739 | 3.18E-122 | MORF4L1P1 | 0.546 | 9.92E-56 |
| FBLIM1 | 0.735 | 3.23E-120 | PELO | 0.546 | 1.01E-55 |
| IFNGR2 | 0.735 | 3.49E-120 | HILPDA | 0.546 | 1.01E-55 |
| TMEM154 | 0.735 | 5.47E-120 | BUB1 | 0.546 | 1.01E-55 |
| BCAT1 | 0.735 | 7.04E-120 | PHTF1 | 0.546 | 1.02E-55 |
| ARAP3 | 0.734 | 1.51E-119 | RPL7AP6 | 0.546 | 1.05E-55 |
| ITGA5 | 0.734 | 2.38E-119 | MICALL2 | 0.546 | 1.06E-55 |
| AC108463.1 | 0.733 | 3.76E-119 | FLNA | 0.546 | 1.07E-55 |
| CCN4 | 0.732 | 2.23E-118 | HSPB1 | 0.546 | 1.07E-55 |
| IL1RAP | 0.731 | 3.74E-118 | AC002401.4 | 0.546 | 1.12E-55 |
| LGALS1 | 0.730 | 1.61E-117 | GGT8P | 0.546 | 1.25E-55 |
| TEAD3 | 0.730 | 1.98E-117 | EEF1A1P25 | 0.546 | 1.29E-55 |
| CHI3L1 | 0.729 | 4.14E-117 | CPVL | 0.545 | 1.40E-55 |
| ABCC3 | 0.728 | 1.11E-116 | CENPU | 0.545 | 1.47E-55 |
| NNMT | 0.727 | 2.13E-116 | RALB | 0.545 | 1.49E-55 |
| PLAT | 0.727 | 3.44E-116 | AC079140.2 | 0.545 | 1.59E-55 |
| PHLDA2 | 0.726 | 6.41E-116 | FTL | 0.545 | 1.65E-55 |
| FMOD | 0.726 | 1.11E-115 | LMO2 | 0.545 | 1.69E-55 |
| TUBA1C | 0.726 | 1.39E-115 | MSTO1 | 0.545 | 1.82E-55 |
| FNDC3B | 0.725 | 2.19E-115 | AL049637.1 | 0.545 | 1.83E-55 |
| DPP4 | 0.725 | 2.32E-115 | B2M | 0.545 | 1.84E-55 |
| PLAU | 0.725 | 2.96E-115 | SEC61A1 | 0.545 | 1.85E-55 |
| ULBP3 | 0.724 | 6.52E-115 | HNRNPA3P6 | 0.545 | 1.90E-55 |
| RHOJ | 0.724 | 1.27E-114 | TCF19 | 0.545 | 1.92E-55 |
| ZYX | 0.723 | 2.40E-114 | EID3 | 0.545 | 1.96E-55 |
| OSTC | 0.723 | 2.46E-114 | FCGRT | 0.545 | 2.10E-55 |
| PDPN | 0.722 | 5.83E-114 | AC015922.3 | 0.545 | 2.25E-55 |
| ITGB1P1 | 0.722 | 5.98E-114 | DESI2 | 0.544 | 2.34E-55 |
| CD63 | 0.721 | 1.66E-113 | SERINC2 | 0.544 | 2.56E-55 |
| SLC25A24 | 0.721 | 1.73E-113 | AC090602.1 | 0.544 | 2.58E-55 |
| CASP4 | 0.720 | 4.36E-113 | NCF1C | 0.544 | 2.66E-55 |
| SH2D4A | 0.720 | 7.61E-113 | FTH1P23 | 0.544 | 2.67E-55 |
| SEC24D | 0.719 | 1.09E-112 | RPSAP61 | 0.544 | 3.03E-55 |
| MMP11 | 0.719 | 1.32E-112 | ACTG1 | 0.544 | 3.06E-55 |
| PLEK2 | 0.719 | 2.10E-112 | RPS15P4 | 0.544 | 3.07E-55 |
| RBMS1 | 0.718 | 2.54E-112 | TMEM70 | 0.544 | 3.21E-55 |
| CD151 | 0.718 | 4.36E-112 | PHB | 0.544 | 3.33E-55 |
| IKBIP | 0.716 | 3.34E-111 | S100A9 | 0.544 | 3.47E-55 |
| COL4A1 | 0.715 | 1.03E-110 | ZBTB8OS | 0.544 | 3.75E-55 |
| VAV3 | 0.714 | 2.39E-110 | SLC9A1 | 0.544 | 3.79E-55 |
| IGF2BP2 | 0.713 | 5.07E-110 | AC005400.1 | 0.544 | 3.83E-55 |
| CLEC5A | 0.713 | 7.45E-110 | CEBPD | 0.544 | 3.89E-55 |
| LAMB1 | 0.713 | 7.86E-110 | BMP1 | 0.543 | 4.03E-55 |
| FSTL1 | 0.713 | 7.94E-110 | NCMAP | 0.543 | 4.31E-55 |
| CALU | 0.712 | 2.07E-109 | HP | 0.543 | 4.31E-55 |
| AF201337.1 | 0.712 | 2.49E-109 | BICD1 | 0.543 | 4.64E-55 |
| P3H1 | 0.711 | 3.74E-109 | ARID5A | 0.543 | 4.73E-55 |
| COL8A1 | 0.711 | 7.90E-109 | AL355309.1 | 0.543 | 4.76E-55 |
| LIF | 0.710 | 1.37E-108 | AEBP1 | 0.543 | 4.87E-55 |
| HTRA3 | 0.710 | 1.66E-108 | LRRN4CL | 0.543 | 4.88E-55 |
| ITGB3 | 0.710 | 1.81E-108 | AC098935.1 | 0.543 | 4.91E-55 |
| FKBP1C | 0.709 | 3.28E-108 | ORC1 | 0.543 | 5.03E-55 |
| COL4A2 | 0.709 | 5.18E-108 | DOK2 | 0.543 | 5.12E-55 |
| LOX | 0.708 | 6.83E-108 | CENPW | 0.543 | 5.17E-55 |
| AL049871.1 | 0.708 | 7.84E-108 | GALNS | 0.543 | 5.33E-55 |
| SPON2 | 0.707 | 2.88E-107 | FZD5 | 0.543 | 5.34E-55 |
| NOX4 | 0.706 | 5.05E-107 | EEF1B2P3 | 0.543 | 5.66E-55 |
| FABP5 | 0.706 | 5.60E-107 | THCAT158 | 0.543 | 6.78E-55 |
| TNFRSF12A | 0.706 | 6.58E-107 | RPS27P21 | 0.542 | 7.03E-55 |
| FKBP1A | 0.706 | 1.20E-106 | H2BC12 | 0.542 | 7.05E-55 |
| HEXB | 0.705 | 1.48E-106 | AL022718.1 | 0.542 | 7.25E-55 |
| SLC43A3 | 0.705 | 1.83E-106 | HDAC7 | 0.542 | 7.50E-55 |
| SHC1 | 0.705 | 1.97E-106 | SLC5A6 | 0.542 | 8.12E-55 |
| TMSB4XP4 | 0.704 | 7.69E-106 | GPRC5A | 0.542 | 8.18E-55 |
| KDELR1 | 0.703 | 9.45E-106 | TCIM | 0.542 | 8.33E-55 |
| EFNB2 | 0.703 | 1.09E-105 | AC079250.1 | 0.542 | 8.85E-55 |
| MEOX2 | 0.703 | 1.29E-105 | EEF1A1P22 | 0.542 | 8.98E-55 |
| HS3ST3B1 | 0.703 | 1.58E-105 | HOTAIRM1 | 0.542 | 9.01E-55 |
| PRICKLE3 | 0.703 | 1.70E-105 | SH2B3 | 0.542 | 9.06E-55 |
| TMEM71 | 0.703 | 1.88E-105 | TSPAN17 | 0.542 | 9.17E-55 |
| LDHA | 0.703 | 1.91E-105 | ULK4 | 0.542 | 9.48E-55 |
| STC1 | 0.702 | 6.28E-105 | TRIP4 | 0.542 | 9.70E-55 |
| FABP5P7 | 0.701 | 1.14E-104 | AC024293.1 | 0.542 | 9.72E-55 |
| ASPN | 0.701 | 1.15E-104 | FCGR3A | 0.542 | 9.83E-55 |
| TENT5A | 0.700 | 3.01E-104 | RPL5P23 | 0.542 | 1.05E-54 |
| EMILIN2 | 0.700 | 4.24E-104 | EMP2 | 0.542 | 1.09E-54 |
| FBXO17 | 0.699 | 5.47E-104 | WBP1P1 | 0.542 | 1.09E-54 |
| PLAUR | 0.699 | 6.79E-104 | CNN3 | 0.542 | 1.14E-54 |
| PINLYP | 0.699 | 1.09E-103 | CRYZL2P | 0.542 | 1.16E-54 |
| GPR157 | 0.698 | 1.27E-103 | FRZB | 0.542 | 1.17E-54 |
| DUSP6 | 0.697 | 5.83E-103 | PTPN2 | 0.541 | 1.24E-54 |
| PLP2 | 0.697 | 5.95E-103 | DSN1 | 0.541 | 1.25E-54 |
| FAM114A1 | 0.697 | 7.31E-103 | BRI3 | 0.541 | 1.28E-54 |
| S100A4 | 0.697 | 7.41E-103 | MED18 | 0.541 | 1.33E-54 |
| RBP1 | 0.696 | 8.20E-103 | MUL1 | 0.541 | 1.36E-54 |
| SPRY4 | 0.696 | 1.76E-102 | GJA4 | 0.541 | 1.47E-54 |
| PKIB | 0.695 | 2.17E-102 | RPL19P21 | 0.541 | 1.58E-54 |
| DPYD | 0.695 | 2.21E-102 | RAD51 | 0.541 | 1.59E-54 |
| TMSB10 | 0.695 | 3.20E-102 | MGAT1 | 0.541 | 1.66E-54 |
| EXOC3L2 | 0.695 | 3.94E-102 | RPL31P63 | 0.541 | 1.68E-54 |
| CD276 | 0.695 | 3.97E-102 | PITX1 | 0.541 | 1.79E-54 |
| TMSB4XP1 | 0.694 | 7.60E-102 | QPCT | 0.541 | 1.82E-54 |
| G0S2 | 0.694 | 9.85E-102 | SPCS2P4 | 0.540 | 2.02E-54 |
| APOBEC3F | 0.693 | 1.98E-101 | SUMO2P21 | 0.540 | 2.09E-54 |
| FAM20C | 0.693 | 1.99E-101 | CEMIP2 | 0.540 | 2.12E-54 |
| COL6A3 | 0.693 | 2.12E-101 | CTF1 | 0.540 | 2.18E-54 |
| BCL3 | 0.692 | 4.48E-101 | CMC1 | 0.540 | 2.23E-54 |
| SNRPGP10 | 0.691 | 9.24E-101 | SRP14P2 | 0.540 | 2.39E-54 |
| EMILIN1 | 0.691 | 9.42E-101 | NDC80 | 0.540 | 2.42E-54 |
| TMSB10P1 | 0.691 | 1.24E-100 | SGSH | 0.540 | 2.46E-54 |
| CD58 | 0.691 | 1.99E-100 | DPH3 | 0.540 | 2.70E-54 |
| OSMR | 0.691 | 2.01E-100 | UBE2C | 0.540 | 2.72E-54 |
| MIR155HG | 0.690 | 2.62E-100 | MMADHC | 0.540 | 2.88E-54 |
| KDELR2 | 0.690 | 4.03E-100 | AC061999.1 | 0.540 | 3.02E-54 |
| SHOX2 | 0.689 | 7.19E-100 | STK40 | 0.540 | 3.02E-54 |
| TMSB4XP8 | 0.688 | 1.47E-99 | HLA-DRA | 0.539 | 3.45E-54 |
| RUBCNL | 0.688 | 1.52E-99 | GSS | 0.539 | 3.48E-54 |
| SDC1 | 0.688 | 1.53E-99 | MAP3K20 | 0.539 | 3.58E-54 |
| SLC27A3 | 0.688 | 2.70E-99 | COL18A1 | 0.539 | 3.97E-54 |
| PDLIM1 | 0.688 | 3.07E-99 | ZNF584 | 0.539 | 4.26E-54 |
| RAB32 | 0.687 | 3.72E-99 | TMEM176B | 0.539 | 4.54E-54 |
| MYDGF | 0.687 | 4.39E-99 | C6orf15 | 0.539 | 4.58E-54 |
| CASP6 | 0.687 | 5.30E-99 | YWHAZP3 | 0.539 | 4.68E-54 |
| PPIC | 0.687 | 6.46E-99 | ORC5 | 0.539 | 5.33E-54 |
| COLGALT1 | 0.686 | 9.47E-99 | AL139100.1 | 0.539 | 5.81E-54 |
| STEAP3 | 0.686 | 1.08E-98 | BET1 | 0.538 | 5.87E-54 |
| METTL7B | 0.686 | 1.12E-98 | MREG | 0.538 | 5.95E-54 |
| MSN | 0.686 | 1.20E-98 | CDC45 | 0.538 | 6.07E-54 |
| TMSB4XP2 | 0.685 | 2.16E-98 | RPL39P15 | 0.538 | 6.19E-54 |
| PIGT | 0.685 | 2.42E-98 | AC110611.2 | 0.538 | 6.77E-54 |
| NIBAN1 | 0.684 | 1.02E-97 | SNRNP40 | 0.538 | 6.81E-54 |
| YIPF1 | 0.683 | 2.02E-97 | ISL2 | 0.538 | 6.88E-54 |
| TES | 0.683 | 2.90E-97 | ZNF416 | 0.538 | 7.40E-54 |
| P4HB | 0.682 | 3.27E-97 | CAMK2D | 0.538 | 7.64E-54 |
| RARRES1 | 0.682 | 5.38E-97 | AL034379.1 | 0.538 | 8.05E-54 |
| AC098614.2 | 0.682 | 5.44E-97 | PRMT6 | 0.538 | 8.25E-54 |
| HFE | 0.682 | 6.32E-97 | RPS26P6 | 0.538 | 8.44E-54 |
| RAP1B | 0.682 | 6.80E-97 | CXCL9 | 0.538 | 8.75E-54 |
| CAVIN3 | 0.681 | 9.43E-97 | SLC35C1 | 0.538 | 9.28E-54 |
| MARVELD1 | 0.681 | 1.05E-96 | UAP1 | 0.538 | 9.49E-54 |
| MXRA7 | 0.681 | 1.15E-96 | TRPV2 | 0.538 | 9.52E-54 |
| PODNL1 | 0.681 | 1.45E-96 | ZNF436 | 0.537 | 1.00E-53 |
| AC113404.3 | 0.681 | 1.59E-96 | TUBB2BP1 | 0.537 | 1.02E-53 |
| AL354714.2 | 0.681 | 1.77E-96 | PRMT1 | 0.537 | 1.03E-53 |
| TMSB4X | 0.680 | 1.82E-96 | NDC1 | 0.537 | 1.11E-53 |
| MYL12B | 0.680 | 1.96E-96 | H2AJ | 0.537 | 1.19E-53 |
| C6orf141 | 0.680 | 2.11E-96 | SSH3 | 0.537 | 1.21E-53 |
| MRC2 | 0.680 | 2.67E-96 | IQGAP3 | 0.537 | 1.21E-53 |
| LINC01614 | 0.680 | 3.37E-96 | RPL23P8 | 0.537 | 1.29E-53 |
| AC243960.2 | 0.679 | 5.93E-96 | COPB1 | 0.537 | 1.30E-53 |
| MMP9 | 0.679 | 7.66E-96 | ZNF561 | 0.537 | 1.32E-53 |
| MXRA7P1 | 0.677 | 2.53E-95 | MTFR1 | 0.537 | 1.36E-53 |
| GLB1 | 0.677 | 3.79E-95 | SCPEP1 | 0.537 | 1.46E-53 |
| PLA2G2A | 0.677 | 4.47E-95 | ATP1B3 | 0.537 | 1.50E-53 |
| CAVIN1 | 0.676 | 7.86E-95 | DMAC2 | 0.537 | 1.55E-53 |
| DDOST | 0.676 | 8.13E-95 | PRELID1P5 | 0.537 | 1.55E-53 |
| PLBD1 | 0.676 | 8.64E-95 | GPX1P1 | 0.537 | 1.57E-53 |
| ADGRE5 | 0.676 | 8.81E-95 | CDKN2C | 0.537 | 1.62E-53 |
| ACTBP2 | 0.676 | 1.08E-94 | VWA1 | 0.537 | 1.65E-53 |
| MPZL3 | 0.675 | 1.46E-94 | LAMTOR5 | 0.536 | 1.71E-53 |
| MAP3K7CL | 0.675 | 1.55E-94 | CNPY4 | 0.536 | 1.81E-53 |
| AC093673.1 | 0.675 | 1.56E-94 | GTF3A | 0.536 | 1.91E-53 |
| PKMP1 | 0.675 | 3.18E-94 | SLC38A6 | 0.536 | 2.11E-53 |
| AL035411.1 | 0.675 | 3.19E-94 | ESYT1 | 0.536 | 2.21E-53 |
| KCNE4 | 0.674 | 3.46E-94 | NCF1 | 0.536 | 2.24E-53 |
| ESM1 | 0.674 | 3.60E-94 | PDCL3 | 0.536 | 2.32E-53 |
| B3GNT7 | 0.674 | 4.20E-94 | HSPB6 | 0.536 | 2.34E-53 |
| VASN | 0.674 | 5.10E-94 | KRT10 | 0.536 | 2.46E-53 |
| GUSB | 0.674 | 5.72E-94 | ZNF816 | 0.536 | 2.48E-53 |
| MAP2K3 | 0.674 | 5.92E-94 | AC092958.1 | 0.536 | 2.54E-53 |
| RAB42 | 0.674 | 7.29E-94 | TMEM109 | 0.536 | 2.84E-53 |
| FTLP3 | 0.673 | 1.25E-93 | SPINT1 | 0.535 | 2.96E-53 |
| GAS2L3 | 0.673 | 1.33E-93 | SPPL2A | 0.535 | 3.33E-53 |
| EOGT | 0.672 | 2.51E-93 | GBP1P1 | 0.535 | 3.37E-53 |
| S100A11 | 0.672 | 3.17E-93 | C1orf174 | 0.535 | 3.62E-53 |
| DIRAS3 | 0.672 | 4.01E-93 | MIPEP | 0.535 | 3.70E-53 |
| CLCF1 | 0.671 | 5.65E-93 | DNAJB1 | 0.535 | 3.82E-53 |
| MXRA5 | 0.671 | 6.96E-93 | ZNF230 | 0.535 | 3.83E-53 |
| TNFRSF11B | 0.671 | 7.22E-93 | RHBDF1 | 0.535 | 4.36E-53 |
| FTLP2 | 0.671 | 8.09E-93 | LINC02609 | 0.535 | 4.63E-53 |
| MPZL2 | 0.670 | 1.16E-92 | MAGT1 | 0.534 | 4.91E-53 |
| CISH | 0.670 | 1.23E-92 | RDH5 | 0.534 | 4.99E-53 |
| NECTIN2 | 0.670 | 1.45E-92 | LOXL3 | 0.534 | 5.25E-53 |
| LSP1 | 0.669 | 4.23E-92 | ETV2 | 0.534 | 5.33E-53 |
| FKBP9P1 | 0.668 | 6.88E-92 | EMP1 | 0.534 | 5.34E-53 |
| AL596087.2 | 0.668 | 7.53E-92 | PTP4A2P1 | 0.534 | 6.00E-53 |
| LAMA2 | 0.668 | 1.08E-91 | ERGIC1 | 0.534 | 6.00E-53 |
| GAPDHP63 | 0.668 | 1.17E-91 | GINS4 | 0.534 | 6.59E-53 |
| CALD1 | 0.667 | 1.95E-91 | PDIA3P1 | 0.534 | 7.23E-53 |
| EHD2 | 0.667 | 2.07E-91 | PSMC1 | 0.534 | 7.53E-53 |
| PFN1P1 | 0.666 | 3.51E-91 | SLC25A39 | 0.533 | 8.51E-53 |
| PYGL | 0.666 | 4.11E-91 | AL441992.1 | 0.533 | 8.66E-53 |
| BCL2L12 | 0.666 | 4.40E-91 | PSMB8 | 0.533 | 8.70E-53 |
| GLA | 0.666 | 4.52E-91 | WTAP | 0.533 | 9.20E-53 |
| MMP14 | 0.666 | 4.60E-91 | CMAHP | 0.533 | 9.30E-53 |
| EDEM2 | 0.666 | 4.69E-91 | RILP | 0.533 | 9.40E-53 |
| LATS2 | 0.666 | 4.90E-91 | ISYNA1 | 0.533 | 9.53E-53 |
| RUNX1 | 0.666 | 6.17E-91 | TRADD | 0.533 | 9.62E-53 |
| NME2P1 | 0.666 | 7.15E-91 | LRRC36 | 0.533 | 9.72E-53 |
| PPIA | 0.665 | 8.63E-91 | POGLUT2 | 0.533 | 9.93E-53 |
| PRKAR1B-AS1 | 0.665 | 8.71E-91 | PGAP6 | 0.533 | 1.01E-52 |
| AP2S1 | 0.665 | 9.82E-91 | IRAK4 | 0.533 | 1.02E-52 |
| TPT1P4 | 0.665 | 1.06E-90 | THSD1 | 0.533 | 1.09E-52 |
| LXN | 0.665 | 1.14E-90 | YWHAEP5 | 0.533 | 1.10E-52 |
| PDIA4 | 0.665 | 1.27E-90 | CD163 | 0.533 | 1.13E-52 |
| GLMP | 0.664 | 2.08E-90 | TXN | 0.533 | 1.17E-52 |
| MDK | 0.664 | 3.56E-90 | ATG4A | 0.533 | 1.18E-52 |
| SERPING1 | 0.663 | 3.86E-90 | POLR2J4 | 0.533 | 1.24E-52 |
| NUCB1 | 0.663 | 4.29E-90 | PSMB4 | 0.532 | 1.42E-52 |
| SYDE1 | 0.663 | 4.65E-90 | CCZ1 | 0.532 | 1.42E-52 |
| SPAG4 | 0.663 | 4.76E-90 | ERP44 | 0.532 | 1.45E-52 |
| ANXA1 | 0.663 | 7.51E-90 | LINC02525 | 0.532 | 1.52E-52 |
| ADAM19 | 0.662 | 1.30E-89 | HLA-B | 0.532 | 1.59E-52 |
| GAPDHP72 | 0.662 | 1.68E-89 | SETD9 | 0.532 | 1.67E-52 |
| XKR8 | 0.662 | 1.99E-89 | STING1 | 0.532 | 1.96E-52 |
| SNAI2 | 0.661 | 2.27E-89 | KCTD9 | 0.532 | 1.97E-52 |
| SMS | 0.661 | 2.41E-89 | TMEM220-AS1 | 0.532 | 2.04E-52 |
| MIR34AHG | 0.661 | 2.47E-89 | TTK | 0.532 | 2.05E-52 |
| FN1 | 0.661 | 3.15E-89 | OPN3 | 0.532 | 2.08E-52 |
| ACTBP11 | 0.661 | 3.16E-89 | PPP4C | 0.532 | 2.09E-52 |
| CDC42P6 | 0.661 | 3.36E-89 | HDAC1 | 0.532 | 2.11E-52 |
| ANXA5 | 0.661 | 3.42E-89 | TOR3A | 0.532 | 2.23E-52 |
| POLR1D | 0.661 | 3.71E-89 | RPL15P3 | 0.531 | 2.35E-52 |
| ITGA4 | 0.660 | 4.64E-89 | FBXO4 | 0.531 | 2.35E-52 |
| BGN | 0.660 | 5.01E-89 | SERPINB6 | 0.531 | 2.44E-52 |
| RAB27A | 0.659 | 1.05E-88 | CENPE | 0.531 | 2.51E-52 |
| SLC66A3 | 0.659 | 1.14E-88 | PTMAP12 | 0.531 | 2.58E-52 |
| VMP1 | 0.659 | 1.41E-88 | ADGRL4 | 0.531 | 2.64E-52 |
| GAPDHP65 | 0.659 | 1.46E-88 | HMOX1 | 0.531 | 2.72E-52 |
| MYL6P5 | 0.659 | 1.71E-88 | HLA-DQA1 | 0.531 | 2.87E-52 |
| SERPINE1 | 0.659 | 1.75E-88 | RUNX3 | 0.531 | 2.88E-52 |
| CAPZA1 | 0.659 | 2.20E-88 | LDHBP2 | 0.531 | 2.98E-52 |
| NAMPT | 0.658 | 2.83E-88 | SELENOS | 0.531 | 3.13E-52 |
| NFKBIZ | 0.658 | 3.23E-88 | EEF1A1P16 | 0.531 | 3.26E-52 |
| RPS2P32 | 0.658 | 3.57E-88 | WDR77 | 0.531 | 3.34E-52 |
| GPX7 | 0.658 | 3.99E-88 | RNF7 | 0.531 | 3.34E-52 |
| GAPDHP60 | 0.658 | 5.22E-88 | ICAM1 | 0.531 | 3.39E-52 |
| RPL22P2 | 0.657 | 6.15E-88 | RPL7AP11 | 0.531 | 3.88E-52 |
| EIF4A1P10 | 0.657 | 9.78E-88 | CCL26 | 0.530 | 4.08E-52 |
| PTMAP2 | 0.656 | 1.73E-87 | RANBP1P1 | 0.530 | 4.41E-52 |
| NRP1 | 0.656 | 1.81E-87 | TNFAIP3 | 0.530 | 4.56E-52 |
| IGF2BP3 | 0.656 | 1.85E-87 | BUD31 | 0.530 | 4.82E-52 |
| PLOD1 | 0.656 | 1.93E-87 | TOP1P1 | 0.530 | 5.01E-52 |
| BOLA3 | 0.656 | 2.00E-87 | AL354710.1 | 0.530 | 5.28E-52 |
| LCTL | 0.656 | 2.31E-87 | RPL39P3 | 0.530 | 5.51E-52 |
| CNIH4 | 0.656 | 2.43E-87 | COMMD7 | 0.530 | 5.83E-52 |
| ACTBP7 | 0.655 | 3.38E-87 | SMIM4 | 0.530 | 5.86E-52 |
| OSBPL10 | 0.655 | 4.63E-87 | TMEM45A | 0.530 | 6.35E-52 |
| TMEM255B | 0.655 | 4.84E-87 | AC096533.1 | 0.530 | 6.43E-52 |
| SDF4 | 0.655 | 5.08E-87 | BTG3 | 0.530 | 6.45E-52 |
| CHPF2 | 0.655 | 5.52E-87 | UBALD2 | 0.529 | 7.21E-52 |
| C1RL | 0.655 | 5.78E-87 | IL2RB | 0.529 | 7.37E-52 |
| ARPC3P3 | 0.654 | 6.09E-87 | MRPL37 | 0.529 | 7.47E-52 |
| ABRACL | 0.654 | 7.03E-87 | PCSK5 | 0.529 | 8.22E-52 |
| LYPLA1 | 0.654 | 8.10E-87 | S100A8 | 0.529 | 8.60E-52 |
| APOBEC3G | 0.654 | 8.84E-87 | AC092535.5 | 0.529 | 8.76E-52 |
| HEBP2 | 0.654 | 9.39E-87 | RPS2P5 | 0.529 | 8.78E-52 |
| ISG20 | 0.654 | 1.20E-86 | LINC00601 | 0.529 | 8.83E-52 |
| PKMP4 | 0.653 | 1.48E-86 | NRM | 0.529 | 9.11E-52 |
| MOXD1 | 0.653 | 1.67E-86 | SPP1 | 0.529 | 9.41E-52 |
| TPM3 | 0.653 | 1.89E-86 | MED31 | 0.529 | 9.49E-52 |
| SOCS3 | 0.653 | 1.98E-86 | CIAO2A | 0.529 | 9.71E-52 |
| ENSAP2 | 0.653 | 2.27E-86 | AL158166.2 | 0.529 | 9.94E-52 |
| HOXB3 | 0.653 | 2.32E-86 | HS3ST3A1 | 0.529 | 1.01E-51 |
| HMGN1P38 | 0.653 | 2.34E-86 | CRYZ | 0.529 | 1.03E-51 |
| STAC | 0.652 | 2.98E-86 | WBP1P2 | 0.529 | 1.09E-51 |
| LINC02587 | 0.652 | 3.31E-86 | RWDD4 | 0.528 | 1.11E-51 |
| TMED9 | 0.652 | 3.49E-86 | RPL3P7 | 0.528 | 1.15E-51 |
| PTGFRN | 0.652 | 3.80E-86 | TROAP | 0.528 | 1.17E-51 |
| PRELID1P6 | 0.652 | 3.93E-86 | IMPDH1 | 0.528 | 1.20E-51 |
| SLC10A3 | 0.652 | 4.65E-86 | COTL1 | 0.528 | 1.23E-51 |
| CHST2 | 0.652 | 5.21E-86 | TRPV4 | 0.528 | 1.23E-51 |
| SOCS1 | 0.652 | 5.45E-86 | PIGC | 0.528 | 1.31E-51 |
| RPS19P1 | 0.652 | 5.83E-86 | TXLNA | 0.528 | 1.37E-51 |
| SERBP1P5 | 0.652 | 6.42E-86 | CCL5 | 0.528 | 1.66E-51 |
| SEMA3F | 0.651 | 6.78E-86 | VPS29 | 0.528 | 1.72E-51 |
| EIF4EP2 | 0.651 | 8.68E-86 | HSPE1P3 | 0.528 | 1.74E-51 |
| SHROOM3 | 0.651 | 9.02E-86 | FREM2 | 0.527 | 2.04E-51 |
| PIK3CD-AS2 | 0.651 | 9.61E-86 | EIF2B3 | 0.527 | 2.11E-51 |
| CDC42EP5 | 0.651 | 1.15E-85 | GALE | 0.527 | 2.14E-51 |
| ARHGAP18 | 0.651 | 1.15E-85 | EOLA1 | 0.527 | 2.20E-51 |
| ARSD | 0.651 | 1.42E-85 | TDO2 | 0.527 | 2.28E-51 |
| BZW1P2 | 0.650 | 1.49E-85 | H3-3A | 0.527 | 2.32E-51 |
| RANP1 | 0.650 | 1.60E-85 | AP1S2 | 0.527 | 2.40E-51 |
| FUCA2 | 0.650 | 1.67E-85 | GMFG | 0.527 | 2.45E-51 |
| GAPDHP38 | 0.650 | 2.66E-85 | NAGA | 0.527 | 2.62E-51 |
| PPIAP31 | 0.650 | 2.73E-85 | CAPZA2 | 0.527 | 2.76E-51 |
| WEE1 | 0.650 | 3.10E-85 | PDK1 | 0.527 | 2.78E-51 |
| ARPC2 | 0.649 | 3.21E-85 | H3P14 | 0.527 | 3.03E-51 |
| PLS3 | 0.649 | 3.72E-85 | HMGN2P6 | 0.526 | 3.07E-51 |
| POGLUT3 | 0.649 | 4.71E-85 | SGO2 | 0.526 | 3.19E-51 |
| EIF4A1P2 | 0.649 | 5.71E-85 | RAN | 0.526 | 3.36E-51 |
| PMM2 | 0.649 | 6.50E-85 | LILRA6 | 0.526 | 3.80E-51 |
| PSMD8 | 0.649 | 6.84E-85 | AC009245.1 | 0.526 | 4.22E-51 |
| HMGN2P5 | 0.648 | 6.97E-85 | SELENOH | 0.526 | 4.59E-51 |
| TPT1P9 | 0.648 | 7.23E-85 | PLXDC1 | 0.526 | 4.60E-51 |
| MCAM | 0.648 | 7.29E-85 | DDB2 | 0.526 | 4.68E-51 |
| B3GNT5 | 0.648 | 7.62E-85 | TACC3 | 0.526 | 4.77E-51 |
| MYO1B | 0.648 | 7.64E-85 | GPNMB | 0.526 | 4.93E-51 |
| GAPDHP40 | 0.648 | 7.72E-85 | RPL35P1 | 0.526 | 4.97E-51 |
| CARD16 | 0.648 | 8.74E-85 | DDIAS | 0.526 | 5.02E-51 |
| CALHM2 | 0.648 | 1.36E-84 | CALM2P2 | 0.525 | 5.24E-51 |
| CASP3 | 0.647 | 1.74E-84 | EXO1 | 0.525 | 5.47E-51 |
| FBLN7 | 0.647 | 2.49E-84 | CSTB | 0.525 | 5.59E-51 |
| FAM20A | 0.647 | 2.91E-84 | PSMA1 | 0.525 | 6.27E-51 |
| VEGFA | 0.646 | 3.41E-84 | COQ2 | 0.525 | 6.29E-51 |
| THBS1 | 0.646 | 3.81E-84 | AP3S1 | 0.525 | 6.33E-51 |
| EYA4 | 0.646 | 6.66E-84 | CXCR4 | 0.525 | 6.50E-51 |
| FCGR2B | 0.645 | 8.27E-84 | ARF1 | 0.525 | 6.75E-51 |
| CAV2 | 0.645 | 1.01E-83 | GBE1 | 0.525 | 7.08E-51 |
| ZNF888 | 0.645 | 1.02E-83 | CNEP1R1 | 0.525 | 7.55E-51 |
| UPP1 | 0.645 | 1.10E-83 | GINS1 | 0.525 | 7.75E-51 |
| AC022968.1 | 0.645 | 1.12E-83 | KCNN4 | 0.525 | 8.04E-51 |
| PTMAP5 | 0.645 | 1.12E-83 | TRIM6 | 0.525 | 8.11E-51 |
| TGIF1 | 0.645 | 1.37E-83 | NEU1 | 0.525 | 8.23E-51 |
| FKBP10 | 0.644 | 1.73E-83 | AC015911.1 | 0.524 | 8.51E-51 |
| VDAC1P1 | 0.644 | 2.68E-83 | NME1 | 0.524 | 9.50E-51 |
| TRAM1 | 0.644 | 3.16E-83 | NRBP1 | 0.524 | 9.55E-51 |
| KIAA0040 | 0.643 | 3.37E-83 | EFNB1 | 0.524 | 9.95E-51 |
| PRDX4 | 0.643 | 3.73E-83 | RPS10P16 | 0.524 | 1.09E-50 |
| XRCC6P2 | 0.643 | 4.10E-83 | HDHD3 | 0.524 | 1.11E-50 |
| IGFBP7 | 0.643 | 4.34E-83 | JHY | 0.524 | 1.13E-50 |
| CTSA | 0.643 | 4.71E-83 | HLA-DRB1 | 0.524 | 1.16E-50 |
| TP73-AS1 | 0.643 | 5.95E-83 | ETV6 | 0.524 | 1.28E-50 |
| SLC2A4RG | 0.643 | 6.00E-83 | UBE2A | 0.523 | 1.43E-50 |
| PDLIM4 | 0.643 | 6.36E-83 | ANTXR2 | 0.523 | 1.52E-50 |
| SPATS2L | 0.643 | 6.40E-83 | C11orf24 | 0.523 | 1.53E-50 |
| POLR2L | 0.643 | 7.14E-83 | PKD2 | 0.523 | 1.62E-50 |
| HSPB1P2 | 0.642 | 7.55E-83 | SERPINA3 | 0.523 | 1.67E-50 |
| DMRTA2 | 0.642 | 9.32E-83 | ORAI1 | 0.523 | 1.73E-50 |
| DAP | 0.642 | 9.86E-83 | SZRD1 | 0.523 | 1.85E-50 |
| C1R | 0.642 | 1.01E-82 | FKBP11 | 0.523 | 1.87E-50 |
| CALR | 0.642 | 1.13E-82 | CKAP2L | 0.523 | 1.89E-50 |
| TPI1P1 | 0.642 | 1.13E-82 | POPDC2 | 0.523 | 1.92E-50 |
| GSAP | 0.642 | 1.31E-82 | SLC50A1 | 0.523 | 1.96E-50 |
| EVC2 | 0.641 | 1.57E-82 | SRA1 | 0.523 | 2.29E-50 |
| PPIAP11 | 0.641 | 1.63E-82 | RPL18AP3 | 0.523 | 2.30E-50 |
| RAB38 | 0.641 | 2.19E-82 | GALNT12 | 0.523 | 2.30E-50 |
| UBE2D3P1 | 0.641 | 2.32E-82 | HOXC4 | 0.522 | 2.54E-50 |
| CUL7 | 0.641 | 2.57E-82 | GAL3ST4 | 0.522 | 2.65E-50 |
| ASB9 | 0.641 | 2.66E-82 | PPIAP29 | 0.522 | 2.69E-50 |
| VASP | 0.641 | 2.70E-82 | LSM12 | 0.522 | 2.70E-50 |
| ECM1 | 0.641 | 2.77E-82 | DNPEP | 0.522 | 2.72E-50 |
| SLC20A1 | 0.641 | 3.06E-82 | CDC25C | 0.522 | 3.02E-50 |
| OLFML2A | 0.641 | 3.25E-82 | AL139421.1 | 0.522 | 3.27E-50 |
| PTMAP9 | 0.640 | 3.46E-82 | TAF13 | 0.522 | 3.43E-50 |
| DEPDC1 | 0.640 | 3.88E-82 | UQCRQ | 0.522 | 3.70E-50 |
| FABP5P1 | 0.640 | 4.01E-82 | SPC24 | 0.521 | 4.00E-50 |
| ATF4P3 | 0.640 | 4.16E-82 | LAMTOR2 | 0.521 | 4.31E-50 |
| RINL | 0.640 | 5.78E-82 | EIF6 | 0.521 | 4.33E-50 |
| ANPEP | 0.640 | 5.91E-82 | MICA | 0.521 | 4.47E-50 |
| PDLIM7 | 0.639 | 7.18E-82 | AREG | 0.521 | 4.74E-50 |
| PABPC3 | 0.639 | 7.22E-82 | TMEM258 | 0.521 | 4.79E-50 |
| TMEM230 | 0.639 | 8.15E-82 | RGS19 | 0.521 | 4.83E-50 |
| GPR3 | 0.639 | 8.80E-82 | AEN | 0.521 | 5.11E-50 |
| FCGR2C | 0.639 | 8.88E-82 | RPS15AP1 | 0.521 | 5.21E-50 |
| CD164 | 0.639 | 1.06E-81 | TNC | 0.521 | 5.22E-50 |
| TUBAP2 | 0.639 | 1.18E-81 | TMEM50B | 0.521 | 5.35E-50 |
| CCNB1 | 0.639 | 1.18E-81 | SLFN12 | 0.521 | 5.39E-50 |
| ATP8B1 | 0.639 | 1.48E-81 | FILIP1L | 0.521 | 5.65E-50 |
| CASP8 | 0.638 | 1.77E-81 | HOXA3 | 0.521 | 5.84E-50 |
| FZD6 | 0.638 | 1.84E-81 | HLA-DPB1 | 0.521 | 5.86E-50 |
| ENG | 0.638 | 2.50E-81 | METRNL | 0.521 | 6.22E-50 |
| MICOS10P2 | 0.638 | 2.53E-81 | CDK7 | 0.520 | 6.57E-50 |
| WIPI1 | 0.638 | 2.65E-81 | FAM78B | 0.520 | 6.74E-50 |
| AC007877.1 | 0.638 | 3.10E-81 | DNAAF5 | 0.520 | 7.12E-50 |
| PPIB | 0.637 | 3.49E-81 | GGCX | 0.520 | 7.23E-50 |
| ELK3 | 0.637 | 4.14E-81 | CLIC4 | 0.520 | 7.54E-50 |
| PLOD3 | 0.637 | 4.75E-81 | MIR222HG | 0.520 | 7.94E-50 |
| SLC2A10 | 0.637 | 4.82E-81 | AL590867.2 | 0.520 | 8.06E-50 |
| PDIA3 | 0.637 | 6.72E-81 | DOK3 | 0.520 | 8.35E-50 |
| ENPEP | 0.636 | 7.26E-81 | ARHGAP11A | 0.520 | 8.47E-50 |
| HSPA7 | 0.636 | 7.69E-81 | ACP2 | 0.520 | 8.64E-50 |
| AL669983.1 | 0.636 | 8.12E-81 | TRIM5 | 0.520 | 8.64E-50 |
| EIF5AP4 | 0.636 | 8.92E-81 | PLK1 | 0.520 | 8.65E-50 |
| RPL5P34 | 0.636 | 9.72E-81 | ATP5F1E | 0.520 | 9.15E-50 |
| PSMC1P1 | 0.636 | 1.07E-80 | GINS2 | 0.520 | 9.76E-50 |
| PLK3 | 0.636 | 1.08E-80 | GPR84 | 0.520 | 9.77E-50 |
| ACTN1 | 0.636 | 1.21E-80 | AC022018.1 | 0.520 | 9.92E-50 |
| RER1 | 0.636 | 1.26E-80 | FBP1 | 0.519 | 1.09E-49 |
| SNHG18 | 0.636 | 1.31E-80 | CLEC11A | 0.519 | 1.21E-49 |
| TSPAN4 | 0.636 | 1.37E-80 | LILRB2 | 0.519 | 1.29E-49 |
| TRDC | 0.636 | 1.41E-80 | NPNT | 0.519 | 1.42E-49 |
| TNFAIP8 | 0.635 | 2.22E-80 | PLXND1 | 0.519 | 1.52E-49 |
| BACE2 | 0.635 | 2.27E-80 | SNRPG | 0.519 | 1.68E-49 |
| STEAP1 | 0.635 | 2.44E-80 | FAM43A | 0.519 | 1.69E-49 |
| DCBLD2 | 0.635 | 2.45E-80 | AL049597.1 | 0.519 | 1.70E-49 |
| CAPNS1 | 0.635 | 2.65E-80 | EEF1A1P12 | 0.519 | 1.71E-49 |
| TMEM183B | 0.635 | 2.80E-80 | RPL13P12 | 0.518 | 1.73E-49 |
| AF279873.1 | 0.635 | 2.88E-80 | H2AC20 | 0.518 | 1.73E-49 |
| TOM1L1 | 0.634 | 3.45E-80 | TM4SF18 | 0.518 | 1.88E-49 |
| NUDT19 | 0.634 | 4.28E-80 | AL591846.1 | 0.518 | 1.91E-49 |
| SH2D2A | 0.634 | 4.61E-80 | HSPB11 | 0.518 | 1.92E-49 |
| BAK1 | 0.634 | 5.93E-80 | EMC3 | 0.518 | 2.01E-49 |
| EIF1P3 | 0.634 | 6.14E-80 | HAUS1 | 0.518 | 2.22E-49 |
| RPN2 | 0.633 | 6.87E-80 | LAP3 | 0.518 | 2.32E-49 |
| IQGAP1 | 0.633 | 7.41E-80 | SNRPB | 0.518 | 2.59E-49 |
| STK17A | 0.633 | 7.55E-80 | NOS2 | 0.518 | 2.75E-49 |
| MRPL36 | 0.633 | 9.47E-80 | MPV17 | 0.517 | 3.03E-49 |
| SAT1 | 0.633 | 1.11E-79 | ERVK9-11 | 0.517 | 3.10E-49 |
| CFI | 0.633 | 1.17E-79 | NEDD8P1 | 0.517 | 3.11E-49 |
| CDR2 | 0.633 | 1.29E-79 | AL512785.1 | 0.517 | 3.20E-49 |
| CARD8-AS1 | 0.632 | 1.45E-79 | SGO1 | 0.517 | 3.39E-49 |
| LMAN2 | 0.632 | 1.70E-79 | SLC35B1 | 0.517 | 3.41E-49 |
| HMGN2P4 | 0.632 | 2.27E-79 | NKG7 | 0.517 | 3.47E-49 |
| RPS2P17 | 0.632 | 2.36E-79 | HLA-DMB | 0.517 | 3.68E-49 |
| SLC30A7 | 0.632 | 2.54E-79 | ARHGDIB | 0.517 | 3.82E-49 |
| TCEA3 | 0.632 | 2.55E-79 | AC004854.2 | 0.517 | 4.10E-49 |
| SPRY2 | 0.632 | 2.67E-79 | TRAPPC3 | 0.517 | 4.22E-49 |
| TMEM214 | 0.631 | 3.00E-79 | AC002075.2 | 0.517 | 4.28E-49 |
| GNG5P2 | 0.631 | 3.34E-79 | CDK1 | 0.517 | 4.32E-49 |
| SELENOTP1 | 0.631 | 3.45E-79 | NMNAT1 | 0.517 | 4.39E-49 |
| CBX3P9 | 0.631 | 3.54E-79 | AC018450.1 | 0.517 | 4.55E-49 |
| SELENOF | 0.631 | 4.30E-79 | CD3E | 0.516 | 5.28E-49 |
| ASL | 0.631 | 5.07E-79 | ALG6 | 0.516 | 5.60E-49 |
| TNFRSF14 | 0.631 | 5.11E-79 | H3P47 | 0.516 | 5.61E-49 |
| SERPINB8 | 0.631 | 5.57E-79 | HMMR | 0.516 | 5.84E-49 |
| AL391416.1 | 0.630 | 6.37E-79 | UNC93B1 | 0.516 | 6.19E-49 |
| PRECSIT | 0.630 | 7.85E-79 | NAB2 | 0.516 | 6.61E-49 |
| CFH | 0.630 | 8.30E-79 | NPM1P6 | 0.516 | 6.77E-49 |
| COL15A1 | 0.630 | 8.50E-79 | RAB7B | 0.516 | 6.99E-49 |
| PARPBP | 0.630 | 8.70E-79 | FXYD5 | 0.516 | 7.06E-49 |
| NDUFA4P1 | 0.630 | 1.13E-78 | PTGES3P1 | 0.515 | 8.07E-49 |
| ITGA3 | 0.629 | 1.28E-78 | C5AR1 | 0.515 | 8.19E-49 |
| EIF2S2P4 | 0.629 | 1.31E-78 | MRPL34 | 0.515 | 8.20E-49 |
| UGCG | 0.629 | 1.36E-78 | MEIS3P1 | 0.515 | 8.54E-49 |
| AC007560.1 | 0.629 | 1.41E-78 | ADAMTS14 | 0.515 | 8.70E-49 |
| MRPS12 | 0.629 | 1.58E-78 | CD52 | 0.515 | 9.57E-49 |
| UBE2SP1 | 0.629 | 1.75E-78 | YDJC | 0.515 | 9.91E-49 |
| MAP1LC3C | 0.629 | 2.14E-78 | TRAM2 | 0.515 | 1.01E-48 |
| UBE2SP2 | 0.629 | 2.14E-78 | ZNF529-AS1 | 0.515 | 1.08E-48 |
| PLIN2 | 0.629 | 2.28E-78 | TUBA1B | 0.515 | 1.15E-48 |
| MMP19 | 0.629 | 2.28E-78 | GAPDHP1 | 0.515 | 1.20E-48 |
| ATP5F1EP2 | 0.628 | 2.75E-78 | BTN2A3P | 0.515 | 1.20E-48 |
| EPHA2 | 0.628 | 3.06E-78 | GIMAP2 | 0.515 | 1.21E-48 |
| FHL3 | 0.628 | 3.07E-78 | TGFB3 | 0.514 | 1.26E-48 |
| TM4SF1 | 0.628 | 3.08E-78 | NUF2 | 0.514 | 1.30E-48 |
| FAS | 0.628 | 3.35E-78 | APOL4 | 0.514 | 1.49E-48 |
| LAMA4 | 0.628 | 3.40E-78 | NCSTN | 0.514 | 1.52E-48 |
| AC131097.3 | 0.628 | 4.17E-78 | SSBP1 | 0.514 | 1.55E-48 |
| RAB1C | 0.628 | 4.88E-78 | RAB8A | 0.514 | 1.71E-48 |
| TMBIM1 | 0.628 | 4.97E-78 | DEPDC1B | 0.514 | 1.72E-48 |
| AC034102.1 | 0.627 | 5.23E-78 | DLL4 | 0.514 | 1.78E-48 |
| IGFBP3 | 0.627 | 5.26E-78 | ALG14 | 0.514 | 1.83E-48 |
| SAA1 | 0.627 | 5.29E-78 | AL356535.1 | 0.514 | 1.83E-48 |
| PFN1 | 0.627 | 5.78E-78 | SMIM10 | 0.514 | 1.94E-48 |
| ZCCHC9 | 0.627 | 7.04E-78 | GTSE1 | 0.514 | 1.94E-48 |
| RPS18P12 | 0.627 | 7.29E-78 | CAP1 | 0.514 | 1.95E-48 |
| LTBR | 0.627 | 7.52E-78 | CLPTM1L | 0.514 | 1.96E-48 |
| CDKN3 | 0.627 | 7.64E-78 | PARP9 | 0.514 | 1.99E-48 |
| KCNE3 | 0.627 | 7.69E-78 | AL078596.1 | 0.513 | 2.06E-48 |
| GJC1 | 0.627 | 7.87E-78 | SKA3 | 0.513 | 2.12E-48 |
| APOBEC3C | 0.627 | 7.89E-78 | HOXC11 | 0.513 | 2.13E-48 |
| SNAI1 | 0.627 | 7.89E-78 | RPS26P3 | 0.513 | 2.19E-48 |
| ARL9 | 0.627 | 8.21E-78 | CKS2 | 0.513 | 2.32E-48 |
| PPP1R3B | 0.627 | 8.71E-78 | RPL37AP8 | 0.513 | 2.45E-48 |
| RPL5P4 | 0.627 | 8.87E-78 | PLVAP | 0.513 | 2.48E-48 |
| AL158050.1 | 0.627 | 9.93E-78 | CCR5 | 0.513 | 2.51E-48 |
| IBSP | 0.626 | 1.13E-77 | IL4I1 | 0.513 | 2.59E-48 |
| UBA52P5 | 0.626 | 1.39E-77 | SCNN1B | 0.513 | 2.60E-48 |
| PPM1M | 0.626 | 1.63E-77 | SSBP4 | 0.513 | 2.81E-48 |
| VKORC1 | 0.626 | 1.71E-77 | ZNF613 | 0.513 | 2.99E-48 |
| SEC61G | 0.626 | 1.80E-77 | IL17RC | 0.513 | 2.99E-48 |
| SKP1P2 | 0.626 | 1.84E-77 | RPL39L | 0.513 | 3.07E-48 |
| SHCBP1 | 0.626 | 2.13E-77 | ELMOD2 | 0.513 | 3.08E-48 |
| RDH10 | 0.626 | 2.17E-77 | HSPE1 | 0.513 | 3.18E-48 |
| CSTA | 0.625 | 2.23E-77 | SLC4A7 | 0.513 | 3.20E-48 |
| SEPTIN2P1 | 0.625 | 2.98E-77 | LEPROT | 0.513 | 3.24E-48 |
| H3P6 | 0.625 | 3.31E-77 | AL049872.1 | 0.512 | 3.43E-48 |
| YBX1P2 | 0.625 | 3.47E-77 | DNM3OS | 0.512 | 3.48E-48 |
| RPL26P36 | 0.624 | 5.19E-77 | ZNF700 | 0.512 | 3.51E-48 |
| COL6A1 | 0.624 | 5.27E-77 | ARMC9 | 0.512 | 3.67E-48 |
| RIN1 | 0.624 | 5.30E-77 | SLC35G2 | 0.512 | 3.68E-48 |
| PGK1P1 | 0.624 | 5.68E-77 | STK32B | 0.512 | 3.88E-48 |
| MYADM | 0.624 | 5.86E-77 | LAPTM4A | 0.512 | 4.32E-48 |
| PLSCR1 | 0.624 | 5.93E-77 | TBC1D1 | 0.512 | 4.55E-48 |
| FOSL1 | 0.624 | 8.01E-77 | SNUPN | 0.512 | 4.68E-48 |
| AK2 | 0.624 | 8.26E-77 | RPS6P25 | 0.512 | 4.99E-48 |
| EIF1P7 | 0.624 | 8.50E-77 | AL138724.1 | 0.512 | 5.35E-48 |
| REXO2 | 0.623 | 9.80E-77 | COL8A2 | 0.511 | 5.47E-48 |
| RPL31P49 | 0.623 | 1.14E-76 | PDGFRB | 0.511 | 5.53E-48 |
| LOXL2 | 0.623 | 1.20E-76 | RPS26P15 | 0.511 | 5.69E-48 |
| TNFRSF1A | 0.623 | 1.25E-76 | DDIT4L | 0.511 | 6.31E-48 |
| ADPGK | 0.623 | 1.36E-76 | CLPP | 0.511 | 6.32E-48 |
| CD93 | 0.622 | 2.03E-76 | SOCS2-AS1 | 0.511 | 6.45E-48 |
| RPS15AP24 | 0.622 | 2.17E-76 | CUBN | 0.511 | 6.48E-48 |
| MANF | 0.622 | 2.18E-76 | DPM1 | 0.511 | 6.61E-48 |
| SLC30A5 | 0.622 | 2.29E-76 | TBXA2R | 0.511 | 6.89E-48 |
| ADPRH | 0.622 | 2.37E-76 | AC008026.1 | 0.511 | 6.91E-48 |
| TRMT112P6 | 0.622 | 2.92E-76 | AC044787.1 | 0.511 | 6.96E-48 |
| PRDX1P1 | 0.622 | 3.00E-76 | HAPLN3 | 0.511 | 7.32E-48 |
| ADH5P4 | 0.622 | 3.14E-76 | TMEM248 | 0.511 | 7.88E-48 |
| RPS19P3 | 0.622 | 3.31E-76 | MS4A6A | 0.511 | 8.08E-48 |
| MRPS15 | 0.622 | 3.42E-76 | RBM47 | 0.511 | 8.34E-48 |
| GSDMD | 0.621 | 4.08E-76 | AC091825.1 | 0.511 | 8.70E-48 |
| AC004057.1 | 0.621 | 4.43E-76 | PDE6G | 0.510 | 9.02E-48 |
| ITGA1 | 0.621 | 4.60E-76 | SDF2L1 | 0.510 | 9.37E-48 |
| PDIA6 | 0.621 | 5.00E-76 | CLP1 | 0.510 | 9.47E-48 |
| NSUN7 | 0.621 | 5.18E-76 | UQCRHL | 0.510 | 9.93E-48 |
| TWF2 | 0.621 | 5.29E-76 | GYS1 | 0.510 | 1.04E-47 |
| FAM126A | 0.621 | 5.47E-76 | FRMD8 | 0.510 | 1.06E-47 |
| MYL9 | 0.621 | 6.22E-76 | HOTAIR | 0.510 | 1.12E-47 |
| JAG1 | 0.621 | 6.67E-76 | FHL2 | 0.510 | 1.13E-47 |
| AC004067.1 | 0.621 | 6.93E-76 | H2BC11 | 0.510 | 1.14E-47 |
| TUBB6 | 0.621 | 7.00E-76 | COMMD8 | 0.510 | 1.15E-47 |
| PSMC2 | 0.620 | 9.13E-76 | MMRN1 | 0.510 | 1.20E-47 |
| SUSD2 | 0.620 | 9.34E-76 | CHEK1 | 0.510 | 1.23E-47 |
| BTF3P10 | 0.620 | 9.73E-76 | AC007182.2 | 0.510 | 1.24E-47 |
| TMEM165 | 0.620 | 9.75E-76 | GMPPB | 0.510 | 1.26E-47 |
| AC110994.2 | 0.620 | 9.89E-76 | FO393411.1 | 0.510 | 1.30E-47 |
| EHD4 | 0.620 | 1.03E-75 | FZD8 | 0.510 | 1.34E-47 |
| QPCTL | 0.620 | 1.04E-75 | TMEM176A | 0.510 | 1.35E-47 |
| TWIST1 | 0.620 | 1.08E-75 | AAGAB | 0.510 | 1.40E-47 |
| ADM | 0.620 | 1.10E-75 | GPR82 | 0.509 | 1.46E-47 |
| AURKA | 0.620 | 1.13E-75 | PDRG1 | 0.509 | 1.50E-47 |
| PSME2P2 | 0.620 | 1.17E-75 | TFB2M | 0.509 | 1.54E-47 |
| RBM8B | 0.620 | 1.24E-75 | HOXA6 | 0.509 | 1.56E-47 |
| H3P16 | 0.620 | 1.41E-75 | C2 | 0.509 | 1.56E-47 |
| ECSCR | 0.620 | 1.51E-75 | PRRX2 | 0.509 | 1.59E-47 |
| C5orf15 | 0.620 | 1.58E-75 | SDHC | 0.509 | 1.70E-47 |
| ARPC5 | 0.619 | 1.64E-75 | APOL1 | 0.509 | 1.77E-47 |
| ACTB | 0.619 | 1.68E-75 | RAB33B | 0.509 | 1.81E-47 |
| FAM3C | 0.619 | 1.96E-75 | AL390755.2 | 0.509 | 1.97E-47 |
| CTSB | 0.619 | 2.32E-75 | BX679664.3 | 0.509 | 2.02E-47 |
| RPS2P7 | 0.619 | 2.45E-75 | SLAMF8 | 0.509 | 2.03E-47 |
| ANG | 0.619 | 3.00E-75 | DRAXIN | 0.509 | 2.24E-47 |
| TMEM230P2 | 0.619 | 3.13E-75 | IL7R | 0.508 | 2.37E-47 |
| RARRES2 | 0.618 | 3.66E-75 | UGP2 | 0.508 | 2.48E-47 |
| CPQ | 0.618 | 3.86E-75 | TPX2 | 0.508 | 2.54E-47 |
| ANGPT2 | 0.618 | 4.56E-75 | DENR | 0.508 | 2.57E-47 |
| RPS4XP1 | 0.618 | 4.69E-75 | NAALADL1 | 0.508 | 2.66E-47 |
| MMP2 | 0.618 | 4.69E-75 | NID1 | 0.508 | 2.83E-47 |
| EIF3CL | 0.618 | 5.07E-75 | VWF | 0.508 | 3.43E-47 |
| TGFB1I1 | 0.617 | 6.53E-75 | TNFRSF10B | 0.508 | 3.51E-47 |
| RPS11P5 | 0.617 | 6.73E-75 | AL589674.1 | 0.508 | 3.51E-47 |
| OAZ1 | 0.617 | 7.31E-75 | FCER1G | 0.508 | 3.67E-47 |
| VIM | 0.617 | 7.67E-75 | TIMM8BP2 | 0.507 | 3.91E-47 |
| NEK6 | 0.617 | 7.67E-75 | EGFLAM | 0.507 | 3.93E-47 |
| TREM1 | 0.617 | 9.39E-75 | MBNL3 | 0.507 | 4.00E-47 |
| DEDD2 | 0.617 | 1.22E-74 | GZMH | 0.507 | 4.16E-47 |
| PRF1 | 0.617 | 1.23E-74 | BIRC3 | 0.507 | 4.36E-47 |
| HNRNPKP4 | 0.617 | 1.26E-74 | SEC61B | 0.507 | 4.58E-47 |
| HSPG2 | 0.616 | 1.29E-74 | SNRPC | 0.507 | 5.45E-47 |
| RPN1 | 0.616 | 1.48E-74 | LRRC42 | 0.507 | 5.49E-47 |
| BZW1 | 0.616 | 1.59E-74 | AC064799.1 | 0.507 | 5.69E-47 |
| DCTD | 0.616 | 1.61E-74 | VAMP8 | 0.507 | 5.71E-47 |
| ARPC3P1 | 0.616 | 1.82E-74 | RAB5IF | 0.507 | 5.95E-47 |
| MGAT4B | 0.616 | 1.87E-74 | PARVB | 0.506 | 6.30E-47 |
| MED8 | 0.616 | 2.31E-74 | AC006001.3 | 0.506 | 6.38E-47 |
| EEF1AKMT4 | 0.616 | 2.40E-74 | RPL13AP7 | 0.506 | 6.41E-47 |
| LBX2-AS1 | 0.616 | 2.49E-74 | CD74 | 0.506 | 7.10E-47 |
| AC021074.1 | 0.615 | 2.91E-74 | TEAD4 | 0.506 | 7.46E-47 |
| GGH | 0.615 | 2.91E-74 | CBLN3 | 0.506 | 7.95E-47 |
| FTH1P10 | 0.615 | 3.33E-74 | SEM1P1 | 0.506 | 8.34E-47 |
| SERTAD1 | 0.615 | 3.37E-74 | RPS15AP12 | 0.506 | 8.53E-47 |
| TGFBI | 0.615 | 3.39E-74 | CENPS | 0.506 | 8.59E-47 |
| RCN3 | 0.615 | 3.42E-74 | LAMB2 | 0.506 | 9.04E-47 |
| MYD88 | 0.615 | 4.16E-74 | GCH1 | 0.506 | 9.07E-47 |
| HSPA5 | 0.615 | 4.68E-74 | MT1L | 0.506 | 9.12E-47 |
| PDGFD | 0.615 | 4.89E-74 | TSPAN9 | 0.506 | 9.48E-47 |
| TPT1P6 | 0.615 | 5.17E-74 | DNAJB6 | 0.505 | 1.02E-46 |
| ZNF217 | 0.615 | 5.18E-74 | RPS27AP16 | 0.505 | 1.03E-46 |
| Z96811.1 | 0.614 | 6.51E-74 | GPI | 0.505 | 1.04E-46 |
| FZD1 | 0.614 | 6.69E-74 | PMP22 | 0.505 | 1.04E-46 |
| TXNP6 | 0.614 | 7.27E-74 | SIVA1 | 0.505 | 1.09E-46 |
| PDIA5 | 0.614 | 7.51E-74 | ARSL | 0.505 | 1.14E-46 |
| GNS | 0.614 | 7.76E-74 | HLA-DPA1 | 0.505 | 1.14E-46 |
| NDUFA4L2 | 0.614 | 7.89E-74 | FLNC | 0.505 | 1.27E-46 |
| SLC16A3 | 0.614 | 8.70E-74 | BX679664.1 | 0.505 | 1.29E-46 |
| SRPRA | 0.614 | 8.79E-74 | SLC29A1 | 0.505 | 1.31E-46 |
| HOXC10 | 0.613 | 1.13E-73 | ACVRL1 | 0.505 | 1.33E-46 |
| FTH1P11 | 0.613 | 1.14E-73 | CRABP2 | 0.505 | 1.44E-46 |
| CHCHD2P6 | 0.613 | 1.16E-73 | TFPI | 0.505 | 1.54E-46 |
| C1orf54 | 0.613 | 1.16E-73 | AC018868.1 | 0.505 | 1.55E-46 |
| TMEM159 | 0.613 | 1.25E-73 | ANO1 | 0.504 | 1.56E-46 |
| FHOD1 | 0.613 | 1.42E-73 | CLSPN | 0.504 | 1.56E-46 |
| CRYBG1 | 0.613 | 1.68E-73 | LAMP3 | 0.504 | 1.58E-46 |
| PROCR | 0.613 | 1.86E-73 | MTCO2P2 | 0.504 | 1.61E-46 |
| NKX2-5 | 0.612 | 2.06E-73 | HOXC13 | 0.504 | 1.65E-46 |
| HSP90AA2P | 0.612 | 2.08E-73 | H2AZ1 | 0.504 | 1.67E-46 |
| PLA2G5 | 0.612 | 2.17E-73 | RASSF1 | 0.504 | 1.79E-46 |
| PPP1CA | 0.612 | 2.18E-73 | PRKACA | 0.504 | 1.82E-46 |
| MXRA8 | 0.612 | 2.24E-73 | LYN | 0.504 | 2.07E-46 |
| BDKRB2 | 0.612 | 2.29E-73 | RPS13P2 | 0.504 | 2.08E-46 |
| AC079944.2 | 0.612 | 2.30E-73 | PPIH | 0.504 | 2.09E-46 |
| AFAP1L1 | 0.612 | 2.37E-73 | SIL1 | 0.504 | 2.09E-46 |
| RNF135 | 0.612 | 2.55E-73 | HLA-DQB1 | 0.504 | 2.26E-46 |
| PPCS | 0.612 | 2.88E-73 | NCF1B | 0.504 | 2.31E-46 |
| CKS1B | 0.612 | 2.93E-73 | TPD52L2 | 0.504 | 2.31E-46 |
| MYCBP | 0.612 | 3.13E-73 | FAM192BP | 0.504 | 2.32E-46 |
| GGACT | 0.612 | 3.65E-73 | SLC2A9 | 0.504 | 2.47E-46 |
| H2AZP3 | 0.612 | 3.91E-73 | CD302 | 0.503 | 2.76E-46 |
| HSPD1P1 | 0.612 | 3.91E-73 | RAP2C | 0.503 | 2.84E-46 |
| AC106820.1 | 0.612 | 4.06E-73 | FKBP7 | 0.503 | 2.92E-46 |
| NFE2L3 | 0.611 | 4.29E-73 | PREB | 0.503 | 2.96E-46 |
| COX7CP1 | 0.611 | 4.36E-73 | GBP5 | 0.503 | 3.14E-46 |
| GADD45A | 0.611 | 4.73E-73 | XRN2 | 0.503 | 3.22E-46 |
| TUBBP1 | 0.611 | 5.26E-73 | ARPC4 | 0.503 | 3.23E-46 |
| C2orf66 | 0.611 | 5.80E-73 | ELOC | 0.503 | 3.26E-46 |
| HYAL2 | 0.611 | 6.26E-73 | AL353691.1 | 0.503 | 3.52E-46 |
| ITPRIPL1 | 0.611 | 6.59E-73 | PAXIP1-AS2 | 0.503 | 3.74E-46 |
| SP100 | 0.611 | 6.61E-73 | RPS26P58 | 0.503 | 3.85E-46 |
| YBX1P1 | 0.611 | 7.46E-73 | AC025171.5 | 0.502 | 4.22E-46 |
| AC138305.3 | 0.611 | 7.53E-73 | HOXB-AS1 | 0.502 | 4.27E-46 |
| AL033519.3 | 0.610 | 8.53E-73 | ATP6V1F | 0.502 | 4.51E-46 |
| DCN | 0.610 | 9.63E-73 | NCAPH | 0.502 | 4.53E-46 |
| AL122020.1 | 0.610 | 9.79E-73 | AURKB | 0.502 | 4.56E-46 |
| CDC42 | 0.610 | 1.12E-72 | LGALS3BP | 0.502 | 4.66E-46 |
| UBBP2 | 0.610 | 1.18E-72 | DOK1 | 0.502 | 5.35E-46 |
| IFITM3 | 0.610 | 1.29E-72 | RPL31P17 | 0.502 | 5.71E-46 |
| LRRC32 | 0.610 | 1.30E-72 | RPS29P17 | 0.502 | 5.93E-46 |
| UBE2S | 0.610 | 1.40E-72 | ARSI | 0.502 | 6.11E-46 |
| HNRNPKP2 | 0.610 | 1.58E-72 | DAP3 | 0.502 | 6.18E-46 |
| AL356653.1 | 0.609 | 1.65E-72 | AQP9 | 0.502 | 6.20E-46 |
| CXCL10 | 0.609 | 1.68E-72 | SIGLEC7 | 0.502 | 6.20E-46 |
| SQOR | 0.609 | 1.76E-72 | ADRM1 | 0.501 | 6.70E-46 |
| ADGRL2 | 0.609 | 1.78E-72 | RPL7AP66 | 0.501 | 6.77E-46 |
| AC020915.2 | 0.609 | 1.84E-72 | TTF2 | 0.501 | 6.83E-46 |
| GGN | 0.609 | 1.92E-72 | LYRM4-AS1 | 0.501 | 6.87E-46 |
| AC125807.1 | 0.609 | 2.22E-72 | DCTPP1 | 0.501 | 6.93E-46 |
| GLRX | 0.609 | 2.49E-72 | VDAC1P8 | 0.501 | 6.98E-46 |
| CTSK | 0.609 | 2.62E-72 | YBX1P10 | 0.501 | 7.39E-46 |
| TRPM8 | 0.609 | 2.69E-72 | MRPL2 | 0.501 | 7.50E-46 |
| AC073861.1 | 0.609 | 2.84E-72 | CHODL | 0.501 | 7.53E-46 |
| AC069218.1 | 0.608 | 3.20E-72 | CHCHD2 | 0.501 | 7.67E-46 |
| SLC22A18 | 0.608 | 3.84E-72 | G6PD | 0.501 | 7.70E-46 |
| PTTG1 | 0.608 | 4.16E-72 | EEF1A1P6 | 0.501 | 7.80E-46 |
| TRAC | 0.608 | 4.55E-72 | HNF4G | 0.501 | 7.83E-46 |
| AL138693.1 | 0.607 | 6.44E-72 | FAM241A | 0.501 | 7.83E-46 |
| CCNYL1 | 0.607 | 6.63E-72 | SLC49A3 | 0.501 | 8.81E-46 |
| AC091429.1 | 0.607 | 8.18E-72 | TRAPPC1 | 0.501 | 8.95E-46 |
| TMED2 | 0.607 | 8.64E-72 | GPX1P2 | 0.501 | 8.96E-46 |
| MYO1G | 0.607 | 9.61E-72 | AL390755.3 | 0.501 | 9.25E-46 |
| COX6A1P2 | 0.607 | 1.02E-71 | EIF3K | 0.501 | 9.71E-46 |
| SLC26A2 | 0.607 | 1.06E-71 | DPAGT1 | 0.501 | 9.77E-46 |
| PMS2P1 | 0.607 | 1.06E-71 | IL2RG | 0.501 | 9.89E-46 |
| AC027307.2 | 0.607 | 1.10E-71 | FADD | 0.501 | 1.02E-45 |
| TAFA3 | 0.607 | 1.13E-71 | C1QTNF2 | 0.500 | 1.12E-45 |
| POP4 | 0.607 | 1.19E-71 | MAP3K6 | 0.500 | 1.14E-45 |
| GSTK1 | 0.606 | 1.27E-71 | AL161421.1 | 0.500 | 1.17E-45 |
| TXNDC17 | 0.606 | 1.30E-71 | PCGF1 | 0.500 | 1.18E-45 |
| AC080023.2 | 0.606 | 1.33E-71 | RPL7AP50 | 0.500 | 1.19E-45 |
| ERI1 | 0.606 | 1.54E-71 | MFSD5 | 0.500 | 1.22E-45 |
| ACTR3 | 0.606 | 1.74E-71 | HLA-C | 0.500 | 1.22E-45 |
| RPL5P1 | 0.606 | 1.85E-71 | TBCA | 0.500 | 1.26E-45 |
| RPS29P3 | 0.606 | 1.90E-71 | TWIST2 | 0.500 | 1.26E-45 |
| LINC01842 | 0.606 | 1.92E-71 | SMN1 | 0.500 | 1.26E-45 |
| ADAMTSL4 | 0.606 | 1.96E-71 | ACBD7 | -0.500 | 1.25E-45 |
| PSME2P1 | 0.606 | 2.27E-71 | AL355073.2 | -0.500 | 1.08E-45 |
| PRPS2 | 0.605 | 2.62E-71 | AC092162.3 | -0.500 | 1.05E-45 |
| HSPB1P1 | 0.605 | 2.80E-71 | LINC00320 | -0.501 | 9.64E-46 |
| ZNF600 | 0.605 | 3.22E-71 | MMD2 | -0.501 | 9.13E-46 |
| KHNYN | 0.605 | 3.67E-71 | AL355474.1 | -0.501 | 9.12E-46 |
| FES | 0.605 | 3.97E-71 | SUSD4 | -0.501 | 9.02E-46 |
| HNRNPUP1 | 0.605 | 4.24E-71 | ARHGAP32 | -0.501 | 8.91E-46 |
| PPIAP6 | 0.605 | 4.38E-71 | CYS1 | -0.501 | 8.79E-46 |
| RPS4XP3 | 0.605 | 4.41E-71 | SMAD7 | -0.501 | 8.76E-46 |
| AC027309.2 | 0.604 | 4.78E-71 | DLG2 | -0.501 | 7.89E-46 |
| MYL6 | 0.604 | 5.01E-71 | SLC9A6 | -0.501 | 6.90E-46 |
| RPS2P55 | 0.604 | 6.01E-71 | ZCCHC24 | -0.501 | 6.69E-46 |
| SWAP70 | 0.604 | 6.78E-71 | PRDM11 | -0.502 | 6.26E-46 |
| CGAS | 0.604 | 6.92E-71 | LIFR-AS1 | -0.502 | 6.21E-46 |
| ULBP2 | 0.604 | 6.92E-71 | RNF150 | -0.502 | 6.03E-46 |
| CEP55 | 0.604 | 7.61E-71 | KLRC3 | -0.502 | 5.64E-46 |
| C1S | 0.604 | 8.13E-71 | ACTR1A | -0.502 | 5.28E-46 |
| DLGAP5 | 0.604 | 8.30E-71 | LPIN1 | -0.502 | 5.20E-46 |
| MSR1 | 0.604 | 8.35E-71 | AC104825.1 | -0.502 | 5.14E-46 |
| CLDN1 | 0.604 | 8.62E-71 | UQCRB | -0.502 | 4.67E-46 |
| INAFM1 | 0.603 | 9.50E-71 | PAQR6 | -0.502 | 4.40E-46 |
| HNRNPA3P5 | 0.603 | 9.60E-71 | LINC01532 | -0.502 | 4.22E-46 |
| CLEC18B | 0.603 | 9.79E-71 | OSGIN2 | -0.503 | 3.96E-46 |
| TBL2 | 0.603 | 1.05E-70 | AL033519.5 | -0.503 | 3.76E-46 |
| GNG12 | 0.603 | 1.11E-70 | PCSK6 | -0.503 | 3.48E-46 |
| SNRPD2 | 0.603 | 1.14E-70 | GRIA2 | -0.503 | 3.17E-46 |
| SPRED3 | 0.603 | 1.16E-70 | ABCC8 | -0.503 | 3.13E-46 |
| CTSC | 0.603 | 1.30E-70 | TMEM63C | -0.503 | 2.91E-46 |
| SETP14 | 0.603 | 1.48E-70 | AL450311.1 | -0.503 | 2.52E-46 |
| AC010343.1 | 0.603 | 1.64E-70 | PLCXD2 | -0.504 | 2.47E-46 |
| HOXA5 | 0.603 | 1.73E-70 | PGAP4 | -0.504 | 2.30E-46 |
| KIF23 | 0.602 | 1.77E-70 | MT-CO2 | -0.504 | 2.22E-46 |
| VAMP5 | 0.602 | 1.80E-70 | GPIHBP1 | -0.504 | 2.19E-46 |
| ECE1 | 0.602 | 1.96E-70 | PNMA3 | -0.504 | 2.14E-46 |
| TEAD2 | 0.602 | 2.08E-70 | KIF13A | -0.504 | 2.03E-46 |
| PI4K2B | 0.602 | 2.62E-70 | FAM222A-AS1 | -0.504 | 1.94E-46 |
| RPS7P10 | 0.602 | 2.66E-70 | GDAP1 | -0.504 | 1.93E-46 |
| SSR3 | 0.602 | 3.14E-70 | BEND7 | -0.504 | 1.76E-46 |
| C9orf64 | 0.602 | 3.29E-70 | NKAIN1 | -0.504 | 1.63E-46 |
| RBBP4P1 | 0.602 | 3.32E-70 | PPP1R9A | -0.505 | 1.51E-46 |
| PRELID1 | 0.602 | 3.36E-70 | AC018648.1 | -0.505 | 1.42E-46 |
| ARHGAP29 | 0.602 | 3.38E-70 | HNRNPH3 | -0.505 | 1.35E-46 |
| CENPA | 0.601 | 3.74E-70 | CHADL | -0.505 | 1.28E-46 |
| CDCA4 | 0.601 | 3.74E-70 | NEGR1 | -0.505 | 1.25E-46 |
| FTH1P4 | 0.601 | 3.86E-70 | AC137723.1 | -0.505 | 1.22E-46 |
| STBD1 | 0.601 | 4.01E-70 | MYH7B | -0.505 | 1.04E-46 |
| ADAM33 | 0.601 | 4.25E-70 | PELI2 | -0.506 | 9.48E-47 |
| UBBP1 | 0.601 | 4.28E-70 | RUSC2 | -0.506 | 7.95E-47 |
| UQCRFS1P1 | 0.601 | 4.40E-70 | LRIG2-DT | -0.506 | 7.84E-47 |
| EIF1AXP1 | 0.601 | 5.68E-70 | MKX | -0.506 | 7.61E-47 |
| SFRP4 | 0.601 | 5.79E-70 | AFF2 | -0.506 | 7.44E-47 |
| ETHE1 | 0.601 | 5.96E-70 | TOX3 | -0.506 | 6.17E-47 |
| CANT1 | 0.601 | 6.47E-70 | KMT2A | -0.507 | 5.65E-47 |
| TRIP6 | 0.600 | 6.85E-70 | SGMS1-AS1 | -0.507 | 5.14E-47 |
| AC024619.4 | 0.600 | 7.07E-70 | RCOR2 | -0.507 | 4.98E-47 |
| MIR22HG | 0.600 | 8.38E-70 | WNT7B | -0.507 | 4.85E-47 |
| ITGB1 | 0.600 | 9.00E-70 | ELAVL2 | -0.507 | 4.83E-47 |
| RRM2 | 0.600 | 9.94E-70 | DGCR2 | -0.507 | 4.74E-47 |
| PTPN7 | 0.599 | 1.30E-69 | GPT2 | -0.507 | 4.49E-47 |
| RPL12P4 | 0.599 | 1.31E-69 | OLIG2 | -0.507 | 4.49E-47 |
| ACTG1P14 | 0.599 | 1.36E-69 | CTTNBP2 | -0.507 | 4.30E-47 |
| SHQ1 | 0.599 | 1.44E-69 | ACTL6B | -0.507 | 4.15E-47 |
| LTF | 0.599 | 1.48E-69 | AC068880.4 | -0.507 | 3.92E-47 |
| DUSP5 | 0.599 | 1.52E-69 | LRRC8A | -0.507 | 3.80E-47 |
| S100A13 | 0.599 | 1.57E-69 | GAREM2 | -0.507 | 3.75E-47 |
| CABP4 | 0.599 | 1.59E-69 | AC008751.3 | -0.507 | 3.74E-47 |
| SMYD2 | 0.599 | 1.73E-69 | GPR162 | -0.508 | 3.27E-47 |
| AC011495.1 | 0.599 | 1.90E-69 | C20orf194 | -0.508 | 3.18E-47 |
| CLDN23 | 0.599 | 2.30E-69 | Z84468.2 | -0.508 | 2.78E-47 |
| TK1 | 0.599 | 2.32E-69 | ARMCX4 | -0.508 | 2.67E-47 |
| AC026401.3 | 0.599 | 2.42E-69 | UPF2 | -0.508 | 2.64E-47 |
| HSPE1P2 | 0.598 | 2.84E-69 | EPB41L4A-AS1 | -0.508 | 2.56E-47 |
| NDUFB1P1 | 0.598 | 3.13E-69 | PBLD | -0.508 | 2.55E-47 |
| RPL24P8 | 0.598 | 3.21E-69 | AC127070.2 | -0.508 | 2.54E-47 |
| MRPL32 | 0.598 | 3.39E-69 | CDH18 | -0.508 | 2.35E-47 |
| AC025458.1 | 0.598 | 3.63E-69 | MACROH2A2 | -0.508 | 2.34E-47 |
| KIF20A | 0.598 | 4.03E-69 | AL022069.3 | -0.509 | 2.21E-47 |
| AL139095.2 | 0.598 | 4.27E-69 | AL591848.3 | -0.509 | 2.03E-47 |
| DSTNP3 | 0.598 | 4.51E-69 | KHDRBS2 | -0.509 | 2.02E-47 |
| E2F7 | 0.598 | 4.52E-69 | DENND11 | -0.509 | 1.70E-47 |
| RPL37P2 | 0.597 | 4.75E-69 | LINC02883 | -0.509 | 1.67E-47 |
| CMTM6 | 0.597 | 5.09E-69 | RUNDC3A-AS1 | -0.509 | 1.64E-47 |
| ATRAID | 0.597 | 5.81E-69 | LHPP | -0.509 | 1.53E-47 |
| KIF18A | 0.597 | 6.55E-69 | GFRA1 | -0.509 | 1.51E-47 |
| CDH6 | 0.597 | 6.70E-69 | SCN3A | -0.510 | 1.41E-47 |
| DNAJC19P9 | 0.597 | 6.85E-69 | HRH3 | -0.510 | 1.27E-47 |
| RPS20P10 | 0.597 | 7.52E-69 | CYP2E1 | -0.510 | 1.22E-47 |
| AL080243.2 | 0.597 | 7.70E-69 | DTX4 | -0.510 | 1.18E-47 |
| ALDH7A1P1 | 0.596 | 1.02E-68 | SLC7A14 | -0.510 | 1.15E-47 |
| UBE2MP1 | 0.596 | 1.03E-68 | GCLC | -0.510 | 1.13E-47 |
| APOBEC3B | 0.596 | 1.04E-68 | PELI3 | -0.510 | 1.07E-47 |
| RPL23AP65 | 0.596 | 1.09E-68 | MIR124-2HG | -0.510 | 9.95E-48 |
| PDGFA | 0.596 | 1.11E-68 | LINC01561 | -0.510 | 9.07E-48 |
| SH3BGRL3 | 0.596 | 1.16E-68 | DGKB | -0.510 | 8.98E-48 |
| ACTN4 | 0.596 | 1.16E-68 | AL391834.1 | -0.510 | 8.79E-48 |
| CDC20 | 0.596 | 1.18E-68 | TERF2IP | -0.511 | 8.72E-48 |
| RPS2P4 | 0.596 | 1.28E-68 | CNTNAP2 | -0.511 | 8.29E-48 |
| ACSS3 | 0.596 | 1.31E-68 | AC079140.6 | -0.511 | 7.51E-48 |
| PRSS23 | 0.596 | 1.51E-68 | UNC79 | -0.511 | 7.24E-48 |
| RNPS1P1 | 0.596 | 1.56E-68 | AC073349.2 | -0.511 | 7.22E-48 |
| SUMO2P1 | 0.595 | 1.74E-68 | WNK2 | -0.511 | 6.74E-48 |
| HOXB2 | 0.595 | 1.81E-68 | ELMO1 | -0.511 | 5.94E-48 |
| EIF1P5 | 0.595 | 1.88E-68 | AL162171.3 | -0.511 | 5.52E-48 |
| TENT5B | 0.595 | 1.90E-68 | ASCC1 | -0.512 | 5.36E-48 |
| RPS10P3 | 0.595 | 2.22E-68 | PLCH2 | -0.512 | 5.19E-48 |
| ROR1 | 0.595 | 2.24E-68 | AL731571.1 | -0.512 | 4.66E-48 |
| PSMA6P1 | 0.595 | 2.26E-68 | ADGRA1 | -0.512 | 4.38E-48 |
| TAGLN | 0.595 | 2.32E-68 | RAPGEF4 | -0.512 | 4.37E-48 |
| SHISA5 | 0.595 | 2.51E-68 | MYH14 | -0.512 | 3.69E-48 |
| PTMAP4 | 0.595 | 2.53E-68 | CELF5 | -0.512 | 3.68E-48 |
| RPSAP19 | 0.595 | 2.54E-68 | AC023024.2 | -0.513 | 3.04E-48 |
| LOXL4 | 0.595 | 2.60E-68 | AC015967.1 | -0.513 | 3.03E-48 |
| SP140L | 0.595 | 2.60E-68 | ACADSB | -0.513 | 3.02E-48 |
| FTH1P12 | 0.595 | 2.76E-68 | EZH1 | -0.513 | 2.87E-48 |
| LZTS1 | 0.595 | 2.85E-68 | ERBB4 | -0.513 | 2.43E-48 |
| ALG3 | 0.595 | 3.23E-68 | AL160313.1 | -0.513 | 2.40E-48 |
| AC022613.1 | 0.594 | 3.48E-68 | FGF12 | -0.513 | 2.34E-48 |
| RPL30P4 | 0.594 | 3.51E-68 | JAKMIP3 | -0.513 | 2.26E-48 |
| AL355032.1 | 0.594 | 3.52E-68 | SLC6A1 | -0.513 | 2.24E-48 |
| CALM2P3 | 0.594 | 3.53E-68 | CHGA | -0.513 | 2.05E-48 |
| PLOD2 | 0.594 | 3.81E-68 | RANBP17 | -0.513 | 2.03E-48 |
| SLC35A2 | 0.594 | 3.90E-68 | DNMBP-AS1 | -0.514 | 1.85E-48 |
| RPL34P18 | 0.594 | 4.53E-68 | AL109615.4 | -0.514 | 1.68E-48 |
| MEDAG | 0.594 | 4.80E-68 | AL353597.1 | -0.514 | 1.63E-48 |
| OST4 | 0.594 | 5.51E-68 | NEU4 | -0.514 | 1.51E-48 |
| RPS15AP11 | 0.594 | 6.22E-68 | AC006059.1 | -0.514 | 1.26E-48 |
| RPL41P2 | 0.593 | 6.37E-68 | IL1RAPL1 | -0.515 | 1.10E-48 |
| GLIPR1 | 0.593 | 6.80E-68 | CMTM4 | -0.515 | 9.97E-49 |
| CD274 | 0.593 | 7.05E-68 | RIPPLY2 | -0.515 | 9.54E-49 |
| YBX3 | 0.593 | 7.34E-68 | GKAP1 | -0.515 | 8.79E-49 |
| C8orf88 | 0.593 | 8.01E-68 | AC026471.2 | -0.515 | 8.58E-49 |
| MYO1C | 0.593 | 8.16E-68 | SORCS3 | -0.516 | 7.32E-49 |
| HIC1 | 0.593 | 8.41E-68 | PSME3IP1 | -0.516 | 7.25E-49 |
| ZBTB42 | 0.593 | 8.50E-68 | NECTIN1 | -0.516 | 5.05E-49 |
| OLFML3 | 0.593 | 9.32E-68 | SMIM18 | -0.516 | 4.79E-49 |
| TRDJ1 | 0.593 | 9.54E-68 | TOB1-AS1 | -0.517 | 4.63E-49 |
| NUP37 | 0.593 | 1.04E-67 | AL590666.2 | -0.517 | 4.57E-49 |
| PI3 | 0.593 | 1.12E-67 | DSCAM | -0.517 | 4.40E-49 |
| MYBPH | 0.592 | 1.20E-67 | NKAIN4 | -0.517 | 4.09E-49 |
| ZNF530 | 0.592 | 1.51E-67 | UBXN1 | -0.517 | 4.00E-49 |
| WWTR1 | 0.592 | 1.58E-67 | AC069549.2 | -0.517 | 3.93E-49 |
| TMEM106C | 0.592 | 1.62E-67 | MT-ND3 | -0.517 | 3.91E-49 |
| PSRC1 | 0.592 | 1.89E-67 | SGMS1 | -0.517 | 3.75E-49 |
| FAAP24 | 0.592 | 2.18E-67 | SYT9 | -0.517 | 3.57E-49 |
| TOMM20P4 | 0.592 | 2.23E-67 | DPYSL4 | -0.517 | 3.50E-49 |
| KPNA2 | 0.591 | 2.38E-67 | ZNF33A | -0.517 | 3.01E-49 |
| RPS15AP38 | 0.591 | 2.43E-67 | UQCRHP1 | -0.517 | 2.93E-49 |
| LAMC3 | 0.591 | 2.44E-67 | SLIT1 | -0.518 | 2.80E-49 |
| SERPINA5 | 0.591 | 2.58E-67 | AP000766.1 | -0.518 | 2.68E-49 |
| RPL10AP2 | 0.591 | 2.76E-67 | NLGN2 | -0.518 | 2.54E-49 |
| FTH1P2 | 0.591 | 2.89E-67 | IGFALS | -0.518 | 2.41E-49 |
| PECAM1 | 0.591 | 3.21E-67 | AC026691.1 | -0.518 | 2.35E-49 |
| RPS7P1 | 0.591 | 3.41E-67 | AL161668.4 | -0.518 | 2.31E-49 |
| CDK6-AS1 | 0.591 | 3.59E-67 | SLC8A2 | -0.518 | 2.15E-49 |
| DES | 0.591 | 3.82E-67 | TTC3 | -0.518 | 2.05E-49 |
| RPSAP15 | 0.591 | 4.11E-67 | TM7SF2 | -0.518 | 1.86E-49 |
| ATP5MDP1 | 0.590 | 4.33E-67 | TSPAN7 | -0.518 | 1.76E-49 |
| AHR | 0.590 | 4.51E-67 | MAGEE1 | -0.519 | 1.54E-49 |
| LMNA | 0.590 | 4.74E-67 | HMGA1P7 | -0.519 | 1.33E-49 |
| IER5L | 0.590 | 5.09E-67 | PLEKHB1 | -0.519 | 1.31E-49 |
| EVA1C | 0.590 | 5.15E-67 | NAP1L2 | -0.519 | 1.13E-49 |
| DPY19L1 | 0.590 | 5.31E-67 | AL353751.1 | -0.520 | 9.28E-50 |
| RPL9P7 | 0.590 | 6.55E-67 | RAB33A | -0.520 | 8.17E-50 |
| PTPN9 | 0.590 | 7.18E-67 | NUMA1 | -0.520 | 8.14E-50 |
| ERGIC3 | 0.590 | 7.25E-67 | INPP1 | -0.520 | 7.76E-50 |
| SBNO2 | 0.590 | 7.38E-67 | FBXL16 | -0.521 | 6.24E-50 |
| ERHP1 | 0.590 | 7.94E-67 | CAMSAP3 | -0.521 | 6.08E-50 |
| RCAN1 | 0.589 | 8.24E-67 | CACNG2 | -0.521 | 5.89E-50 |
| CNIH3 | 0.589 | 8.44E-67 | TPTE2P1 | -0.521 | 5.51E-50 |
| SBF2-AS1 | 0.589 | 1.04E-66 | AL118558.3 | -0.521 | 5.26E-50 |
| TMEM14DP | 0.589 | 1.20E-66 | RND1 | -0.521 | 4.93E-50 |
| RARS1 | 0.589 | 1.25E-66 | APC2 | -0.521 | 4.45E-50 |
| DYNLT3 | 0.589 | 1.30E-66 | Z95115.1 | -0.521 | 4.45E-50 |
| LIMS1 | 0.589 | 1.39E-66 | LINC00463 | -0.521 | 4.23E-50 |
| AL138785.1 | 0.588 | 1.55E-66 | AC010175.1 | -0.521 | 3.86E-50 |
| AL109766.1 | 0.588 | 1.58E-66 | LGR5 | -0.522 | 3.83E-50 |
| AC099560.2 | 0.588 | 1.73E-66 | AC000068.2 | -0.522 | 3.79E-50 |
| FCHSD1 | 0.588 | 1.74E-66 | CHGB | -0.522 | 3.61E-50 |
| NPM1P27 | 0.588 | 1.82E-66 | ATCAY | -0.522 | 3.56E-50 |
| AL049873.1 | 0.588 | 1.82E-66 | ALDH6A1 | -0.522 | 3.44E-50 |
| CENPK | 0.588 | 1.88E-66 | FBLL1 | -0.522 | 2.66E-50 |
| PNO1 | 0.588 | 1.95E-66 | PHBP4 | -0.522 | 2.59E-50 |
| RPL10AP6 | 0.588 | 2.08E-66 | LINC02440 | -0.522 | 2.52E-50 |
| CCDC8 | 0.588 | 2.18E-66 | COX15 | -0.523 | 2.17E-50 |
| LINC00900 | 0.588 | 2.21E-66 | ADCYAP1R1 | -0.523 | 1.74E-50 |
| SP6 | 0.588 | 2.49E-66 | GREB1L | -0.523 | 1.72E-50 |
| AC061992.2 | 0.588 | 2.63E-66 | TMEM151B | -0.523 | 1.57E-50 |
| CDC6 | 0.588 | 2.71E-66 | ZFHX2-AS1 | -0.523 | 1.55E-50 |
| TYMS | 0.587 | 3.04E-66 | GNAI1 | -0.524 | 1.28E-50 |
| GNG11 | 0.587 | 3.37E-66 | LINC02731 | -0.524 | 1.09E-50 |
| RPS29P5 | 0.587 | 3.38E-66 | HIP1R | -0.524 | 9.29E-51 |
| MFAP2 | 0.587 | 3.38E-66 | SGCD | -0.524 | 9.27E-51 |
| IGFBP4 | 0.587 | 3.39E-66 | AMER3 | -0.524 | 9.26E-51 |
| CAST | 0.587 | 3.58E-66 | ID2-AS1 | -0.524 | 9.00E-51 |
| H19 | 0.587 | 3.65E-66 | KCNJ11 | -0.524 | 8.89E-51 |
| RPS4XP11 | 0.587 | 3.69E-66 | KAT6B | -0.525 | 7.03E-51 |
| CDK2 | 0.587 | 3.76E-66 | OGA | -0.525 | 5.84E-51 |
| ERRFI1 | 0.587 | 3.85E-66 | MLLT6 | -0.525 | 5.81E-51 |
| MRGBP | 0.587 | 4.06E-66 | ZNF710-AS1 | -0.525 | 5.73E-51 |
| HRH1 | 0.587 | 4.07E-66 | SPTAN1 | -0.525 | 5.65E-51 |
| RPS27AP5 | 0.587 | 4.42E-66 | LHFPL4 | -0.525 | 5.32E-51 |
| AQP5 | 0.587 | 4.47E-66 | DDX25 | -0.525 | 5.07E-51 |
| TMEM51 | 0.587 | 4.48E-66 | AL049749.1 | -0.526 | 4.78E-51 |
| AL031727.1 | 0.587 | 5.05E-66 | FSTL5 | -0.526 | 4.16E-51 |
| NOP10 | 0.587 | 5.05E-66 | AC009102.2 | -0.526 | 3.92E-51 |
| HIGD1AP1 | 0.586 | 5.33E-66 | ST8SIA3 | -0.527 | 3.01E-51 |
| TRAPPC2B | 0.586 | 5.70E-66 | AC016405.3 | -0.527 | 2.98E-51 |
| TSTD1 | 0.586 | 5.70E-66 | TRAM1L1 | -0.527 | 2.92E-51 |
| RAC1P2 | 0.586 | 6.15E-66 | BCAN | -0.527 | 2.80E-51 |
| CD101 | 0.586 | 6.19E-66 | Z97653.1 | -0.527 | 2.21E-51 |
| CHAF1B | 0.586 | 6.19E-66 | AL133216.2 | -0.527 | 1.87E-51 |
| PTMAP8 | 0.586 | 6.31E-66 | RBP3 | -0.527 | 1.86E-51 |
| MLX | 0.586 | 6.68E-66 | AL160270.1 | -0.528 | 1.59E-51 |
| HLA-A | 0.586 | 7.17E-66 | MAST1 | -0.528 | 1.42E-51 |
| SPOCD1 | 0.586 | 7.23E-66 | AC072052.1 | -0.528 | 1.41E-51 |
| FBLN5 | 0.586 | 7.41E-66 | TMEM100 | -0.528 | 1.37E-51 |
| Z74021.1 | 0.586 | 8.09E-66 | RPS6KA6 | -0.528 | 1.15E-51 |
| NACA2 | 0.586 | 8.30E-66 | AC012213.4 | -0.528 | 1.10E-51 |
| CYTL1 | 0.586 | 8.32E-66 | KIAA1755 | -0.529 | 9.45E-52 |
| TRPM4 | 0.585 | 9.92E-66 | AL365259.1 | -0.529 | 7.95E-52 |
| AL162424.1 | 0.585 | 1.10E-65 | LYPLAL1-DT | -0.529 | 7.71E-52 |
| SEL1L3 | 0.585 | 1.11E-65 | EPOP | -0.529 | 7.69E-52 |
| SNRPGP2 | 0.585 | 1.15E-65 | HTATSF1 | -0.529 | 7.02E-52 |
| MGST2 | 0.585 | 1.22E-65 | NOL4 | -0.529 | 6.96E-52 |
| HMGN2P3 | 0.585 | 1.27E-65 | GPR158 | -0.530 | 6.51E-52 |
| ATF5 | 0.585 | 1.28E-65 | AATK | -0.530 | 6.29E-52 |
| H3-5 | 0.585 | 1.32E-65 | CCDC92B | -0.530 | 5.81E-52 |
| HOXD10 | 0.585 | 1.34E-65 | MAPK8 | -0.530 | 5.29E-52 |
| RPL34P27 | 0.585 | 1.36E-65 | ZBTB20-AS4 | -0.530 | 4.82E-52 |
| S100A6 | 0.585 | 1.39E-65 | SRGAP3 | -0.530 | 4.70E-52 |
| SUB1P3 | 0.585 | 1.41E-65 | KCNN2 | -0.530 | 4.46E-52 |
| RPL12P12 | 0.585 | 1.42E-65 | ATP1A3 | -0.531 | 3.50E-52 |
| UBE2V1P2 | 0.585 | 1.42E-65 | RASGRF1 | -0.531 | 3.37E-52 |
| XBP1 | 0.585 | 1.46E-65 | TMEM121B | -0.531 | 3.01E-52 |
| ARF4 | 0.584 | 1.85E-65 | ABAT | -0.531 | 2.53E-52 |
| EEF1A1P17 | 0.584 | 2.02E-65 | USP20 | -0.531 | 2.52E-52 |
| CASP7 | 0.584 | 2.20E-65 | CDS2 | -0.532 | 2.25E-52 |
| CLEC2B | 0.584 | 2.23E-65 | KCNN3 | -0.532 | 2.07E-52 |
| LTBP2 | 0.584 | 2.28E-65 | AL162511.1 | -0.532 | 1.64E-52 |
| AL354919.2 | 0.584 | 2.31E-65 | DOK6 | -0.532 | 1.50E-52 |
| AC011005.1 | 0.584 | 2.32E-65 | ANKRD26 | -0.532 | 1.44E-52 |
| SOD3 | 0.584 | 2.40E-65 | NYAP1 | -0.532 | 1.42E-52 |
| TCIRG1 | 0.584 | 2.41E-65 | MTMR7 | -0.532 | 1.38E-52 |
| MAB21L1 | 0.584 | 2.58E-65 | RAB6B | -0.533 | 1.02E-52 |
| COL12A1 | 0.584 | 2.63E-65 | DGCR5 | -0.533 | 1.00E-52 |
| ORMDL2 | 0.584 | 2.68E-65 | AC009041.1 | -0.533 | 9.11E-53 |
| ATP5MF | 0.584 | 2.83E-65 | CBX7 | -0.533 | 8.16E-53 |
| SGMS2 | 0.584 | 2.86E-65 | CNRIP1 | -0.534 | 7.54E-53 |
| PSMA7 | 0.584 | 2.91E-65 | ARPP21-AS1 | -0.534 | 7.04E-53 |
| MTMR11 | 0.583 | 3.82E-65 | AKR1C3 | -0.534 | 6.83E-53 |
| RPS3AP25 | 0.583 | 4.17E-65 | PLEKHG3 | -0.534 | 5.60E-53 |
| IAH1 | 0.583 | 4.40E-65 | LRRTM4 | -0.534 | 5.22E-53 |
| ATP5MC2P4 | 0.583 | 4.41E-65 | WDR37 | -0.535 | 4.57E-53 |
| MALSU1 | 0.583 | 4.46E-65 | IDI1 | -0.535 | 3.95E-53 |
| STRA6 | 0.583 | 5.01E-65 | HDAC5 | -0.535 | 3.83E-53 |
| BATF3 | 0.583 | 5.07E-65 | GRIA4 | -0.535 | 3.01E-53 |
| IFITM3P2 | 0.583 | 5.09E-65 | AC007938.1 | -0.536 | 2.61E-53 |
| ITGA7 | 0.583 | 5.22E-65 | BRSK2 | -0.536 | 2.59E-53 |
| RPS7P11 | 0.583 | 5.26E-65 | IL17D | -0.536 | 2.56E-53 |
| ASF1B | 0.583 | 5.37E-65 | PARTICL | -0.536 | 2.32E-53 |
| ACE | 0.583 | 6.30E-65 | DGKI | -0.536 | 2.24E-53 |
| ZDHHC12 | 0.582 | 6.67E-65 | PPM1K | -0.536 | 1.95E-53 |
| ARNTL2 | 0.582 | 6.94E-65 | LSAMP | -0.536 | 1.80E-53 |
| PGK1 | 0.582 | 7.65E-65 | AL645608.6 | -0.536 | 1.79E-53 |
| AOX1 | 0.582 | 8.87E-65 | ABI1 | -0.537 | 1.66E-53 |
| SERTAD3 | 0.582 | 9.41E-65 | MICU1 | -0.537 | 1.21E-53 |
| FAUP1 | 0.582 | 9.81E-65 | CDK5R1 | -0.537 | 1.20E-53 |
| ADAMTS3 | 0.582 | 1.04E-64 | DLGAP1 | -0.537 | 1.19E-53 |
| TNFRSF10C | 0.582 | 1.07E-64 | FAM167A | -0.537 | 1.10E-53 |
| AC104619.3 | 0.582 | 1.10E-64 | MTCO1P12 | -0.538 | 9.66E-54 |
| ETV7 | 0.582 | 1.11E-64 | PRR36 | -0.538 | 8.30E-54 |
| TTYH3 | 0.582 | 1.13E-64 | MT-TF | -0.538 | 8.19E-54 |
| TTC38 | 0.582 | 1.13E-64 | AC024730.1 | -0.538 | 8.15E-54 |
| CCNA2 | 0.581 | 1.21E-64 | AC109439.1 | -0.538 | 7.74E-54 |
| KIRREL1 | 0.581 | 1.22E-64 | RGR | -0.538 | 7.65E-54 |
| ST8SIA4 | 0.581 | 1.25E-64 | UPK2 | -0.538 | 6.96E-54 |
| GPN1 | 0.581 | 1.29E-64 | TMEM170B | -0.538 | 6.88E-54 |
| NID2 | 0.581 | 1.30E-64 | PHLPP1 | -0.538 | 6.72E-54 |
| RPSAP17 | 0.581 | 1.50E-64 | RBSN | -0.538 | 6.47E-54 |
| KLHDC8A | 0.581 | 1.63E-64 | ASRGL1 | -0.538 | 6.22E-54 |
| CACYBPP2 | 0.581 | 1.63E-64 | ACSL6 | -0.539 | 5.23E-54 |
| RFC2 | 0.581 | 1.68E-64 | KCNIP2 | -0.539 | 4.74E-54 |
| RPS3AP6 | 0.581 | 1.88E-64 | PID1 | -0.539 | 4.06E-54 |
| P3H2 | 0.581 | 2.02E-64 | TMEM8B | -0.539 | 3.69E-54 |
| TNFRSF19 | 0.581 | 2.14E-64 | KCNH8 | -0.539 | 3.45E-54 |
| RAP2B | 0.580 | 2.27E-64 | AF131216.3 | -0.540 | 3.24E-54 |
| CDCA2 | 0.580 | 2.37E-64 | AC254562.3 | -0.540 | 3.17E-54 |
| LYZ | 0.580 | 2.52E-64 | LRRC4 | -0.540 | 3.03E-54 |
| RPS4XP6 | 0.580 | 2.62E-64 | SCAPER | -0.540 | 2.92E-54 |
| AL358472.1 | 0.580 | 2.72E-64 | MIR6071 | -0.540 | 2.67E-54 |
| NPM1P39 | 0.580 | 2.80E-64 | FAM110B | -0.540 | 2.61E-54 |
| RRAS | 0.580 | 3.19E-64 | AC083864.2 | -0.540 | 2.01E-54 |
| EEF1A1P38 | 0.580 | 3.24E-64 | WAC | -0.541 | 1.93E-54 |
| TRIAP1 | 0.580 | 3.41E-64 | CNTN3 | -0.541 | 1.83E-54 |
| CD40 | 0.580 | 3.55E-64 | DIP2C | -0.541 | 1.71E-54 |
| SERPINA1 | 0.580 | 3.62E-64 | AC233723.2 | -0.541 | 1.47E-54 |
| CDCA8 | 0.579 | 4.21E-64 | ADD3 | -0.541 | 1.46E-54 |
| THBD | 0.579 | 4.68E-64 | PPP1R12B | -0.541 | 1.34E-54 |
| RAC2 | 0.579 | 4.71E-64 | MIR325HG | -0.541 | 1.28E-54 |
| NXT1 | 0.579 | 4.92E-64 | DZIP3 | -0.541 | 1.26E-54 |
| AC098591.3 | 0.579 | 4.92E-64 | PAAF1 | -0.542 | 1.11E-54 |
| TCAF2 | 0.579 | 4.95E-64 | ZCCHC18 | -0.542 | 1.09E-54 |
| CENPL | 0.579 | 5.48E-64 | LRIG1 | -0.542 | 1.05E-54 |
| LRR1 | 0.579 | 6.51E-64 | MAP1A | -0.542 | 1.01E-54 |
| AC007683.1 | 0.579 | 6.53E-64 | AC053513.1 | -0.542 | 9.98E-55 |
| ARL4C | 0.579 | 6.85E-64 | PABPC5 | -0.542 | 8.66E-55 |
| BX470102.1 | 0.579 | 7.01E-64 | WBP2 | -0.542 | 7.77E-55 |
| RPL15P2 | 0.578 | 7.58E-64 | STX1B | -0.542 | 7.23E-55 |
| TFPI2 | 0.578 | 8.13E-64 | PPP1R9A-AS1 | -0.542 | 7.22E-55 |
| HDAC3 | 0.578 | 8.17E-64 | HMBOX1 | -0.542 | 6.90E-55 |
| NUAK2 | 0.578 | 8.57E-64 | AC012645.1 | -0.543 | 6.26E-55 |
| TMEM158 | 0.578 | 8.73E-64 | GRAMD1B | -0.543 | 6.18E-55 |
| TUBBP2 | 0.578 | 8.84E-64 | AC068643.1 | -0.543 | 4.22E-55 |
| EIF4HP1 | 0.578 | 8.94E-64 | KIF3A | -0.543 | 4.19E-55 |
| LSM10 | 0.578 | 9.13E-64 | LRRC20 | -0.543 | 4.10E-55 |
| DTX2 | 0.578 | 9.34E-64 | NRXN1 | -0.544 | 2.87E-55 |
| TRIM38 | 0.578 | 1.02E-63 | CNTN1 | -0.544 | 2.83E-55 |
| AC007688.2 | 0.578 | 1.11E-63 | LRRC1 | -0.544 | 2.34E-55 |
| MGP | 0.578 | 1.19E-63 | PSD | -0.545 | 1.84E-55 |
| IL2RA | 0.578 | 1.24E-63 | SMAD9 | -0.545 | 1.79E-55 |
| RPL37P23 | 0.578 | 1.26E-63 | ZNF385C | -0.545 | 1.40E-55 |
| HSD3B7 | 0.578 | 1.28E-63 | SCAMP5 | -0.546 | 1.34E-55 |
| GTF2E2 | 0.578 | 1.30E-63 | AL353597.2 | -0.546 | 1.19E-55 |
| TWF1P1 | 0.577 | 1.47E-63 | SLC22A17 | -0.546 | 9.52E-56 |
| RPL14P3 | 0.577 | 1.61E-63 | GDNF-AS1 | -0.547 | 7.80E-56 |
| RHOD | 0.577 | 1.62E-63 | PLPPR1 | -0.547 | 6.82E-56 |
| ZNF468 | 0.577 | 1.83E-63 | KSR2 | -0.547 | 5.63E-56 |
| RPSAP12 | 0.577 | 1.87E-63 | LINC00672 | -0.547 | 5.40E-56 |
| IGFBP5 | 0.577 | 1.90E-63 | SLITRK1 | -0.547 | 5.12E-56 |
| PXDNL | 0.577 | 1.98E-63 | MORN4 | -0.547 | 4.77E-56 |
| NHP2P1 | 0.577 | 1.99E-63 | TMEFF2 | -0.548 | 4.51E-56 |
| AC092809.3 | 0.577 | 2.13E-63 | FO681492.1 | -0.548 | 4.49E-56 |
| ZNF552 | 0.577 | 2.20E-63 | FAM155A | -0.548 | 4.46E-56 |
| PDCD1LG2 | 0.577 | 2.25E-63 | RUFY3 | -0.548 | 3.71E-56 |
| RPL9P18 | 0.577 | 2.33E-63 | AC061708.1 | -0.548 | 3.38E-56 |
| RCAN3 | 0.577 | 2.37E-63 | MYORG | -0.548 | 3.33E-56 |
| HOXD11 | 0.577 | 2.41E-63 | ZNF423 | -0.548 | 3.30E-56 |
| CEP112 | 0.577 | 2.43E-63 | MCMDC2 | -0.548 | 2.87E-56 |
| FOXS1 | 0.576 | 2.64E-63 | STOX1 | -0.549 | 2.47E-56 |
| CENPN | 0.576 | 2.85E-63 | NMNAT2 | -0.549 | 2.36E-56 |
| RPL15P18 | 0.576 | 2.86E-63 | TAPT1 | -0.549 | 2.34E-56 |
| ZWILCH | 0.576 | 2.91E-63 | AL023806.1 | -0.549 | 2.33E-56 |
| TMEM37 | 0.576 | 3.28E-63 | C1orf198 | -0.549 | 2.13E-56 |
| GRWD1 | 0.576 | 3.33E-63 | GRID1 | -0.549 | 2.07E-56 |
| OSTM1 | 0.576 | 3.41E-63 | DLL3 | -0.549 | 2.05E-56 |
| UBL5P2 | 0.576 | 3.42E-63 | CNNM2 | -0.549 | 1.82E-56 |
| LRRFIP1 | 0.576 | 3.73E-63 | BEX4 | -0.550 | 1.39E-56 |
| PTMA | 0.576 | 3.78E-63 | BTBD17 | -0.550 | 1.34E-56 |
| AC080038.1 | 0.576 | 4.10E-63 | CYTH1 | -0.550 | 1.30E-56 |
| CA9 | 0.576 | 4.37E-63 | LINC02283 | -0.550 | 1.27E-56 |
| TWSG1 | 0.575 | 4.68E-63 | ZDHHC11B | -0.550 | 9.79E-57 |
| H3P36 | 0.575 | 4.72E-63 | ASXL3 | -0.550 | 9.40E-57 |
| MAGOH | 0.575 | 5.05E-63 | DEGS2 | -0.550 | 9.38E-57 |
| PTGER4 | 0.575 | 5.73E-63 | ELFN2 | -0.551 | 8.35E-57 |
| YIPF2 | 0.575 | 5.94E-63 | RAB18 | -0.551 | 7.84E-57 |
| RPL9P3 | 0.575 | 6.02E-63 | AC013265.1 | -0.551 | 7.58E-57 |
| GNG5 | 0.575 | 7.01E-63 | NCAM2 | -0.551 | 7.01E-57 |
| BAX | 0.575 | 7.09E-63 | NDRG3 | -0.551 | 6.75E-57 |
| APCDD1L | 0.575 | 7.59E-63 | PDZD8 | -0.551 | 6.64E-57 |
| POLE2 | 0.575 | 8.21E-63 | AC120036.3 | -0.551 | 5.76E-57 |
| EPHB4 | 0.574 | 9.13E-63 | AC025211.1 | -0.551 | 5.71E-57 |
| F11R | 0.574 | 9.39E-63 | TCEAL3 | -0.551 | 5.05E-57 |
| RPL13AP6 | 0.574 | 1.04E-62 | BMP2 | -0.552 | 4.69E-57 |
| LENG9 | 0.574 | 1.29E-62 | MT-ND4 | -0.552 | 4.39E-57 |
| LINC01711 | 0.574 | 1.41E-62 | SCRT1 | -0.552 | 4.01E-57 |
| MEST | 0.574 | 1.42E-62 | EFHD1 | -0.552 | 3.24E-57 |
| AC097658.1 | 0.574 | 1.44E-62 | HLF | -0.552 | 2.90E-57 |
| RIPK1 | 0.574 | 1.45E-62 | PPP1R3E | -0.553 | 2.81E-57 |
| FAM86C1P | 0.573 | 1.55E-62 | POU6F1 | -0.553 | 2.30E-57 |
| LGALS8 | 0.573 | 1.63E-62 | SEMA4G | -0.553 | 1.99E-57 |
| GON7 | 0.573 | 1.86E-62 | GABRA3 | -0.553 | 1.83E-57 |
| PVR | 0.573 | 1.94E-62 | RFTN2 | -0.553 | 1.79E-57 |
| RGS16 | 0.573 | 2.16E-62 | APBB1 | -0.553 | 1.70E-57 |
| AC012085.1 | 0.573 | 2.21E-62 | CUX2 | -0.554 | 1.38E-57 |
| ZNF799 | 0.573 | 2.23E-62 | CARMIL3 | -0.554 | 1.31E-57 |
| SERPINB1 | 0.573 | 2.35E-62 | IRAG1 | -0.554 | 1.23E-57 |
| RPSAP58 | 0.573 | 2.48E-62 | RPL7P38 | -0.554 | 1.22E-57 |
| CTSL | 0.573 | 2.51E-62 | ACVR2B | -0.554 | 1.16E-57 |
| AC024940.2 | 0.573 | 2.60E-62 | AC007786.1 | -0.554 | 1.14E-57 |
| CASP1 | 0.573 | 2.71E-62 | SERP2 | -0.554 | 1.09E-57 |
| PIPSL | 0.573 | 2.72E-62 | MOB3B | -0.554 | 1.08E-57 |
| AC020899.1 | 0.573 | 2.74E-62 | AL589826.2 | -0.555 | 9.06E-58 |
| SRPRB | 0.572 | 2.81E-62 | UGT8 | -0.555 | 8.81E-58 |
| UBASH3B | 0.572 | 2.90E-62 | CAMK2G | -0.555 | 8.13E-58 |
| CYP2S1 | 0.572 | 2.94E-62 | SLC25A41 | -0.555 | 7.24E-58 |
| TNFRSF4 | 0.572 | 2.98E-62 | TRAPPC5 | -0.555 | 6.60E-58 |
| HSPE1P4 | 0.572 | 3.02E-62 | IKZF5 | -0.555 | 6.45E-58 |
| PCNA | 0.572 | 3.69E-62 | TMOD2 | -0.555 | 6.18E-58 |
| AC092115.1 | 0.572 | 3.92E-62 | AL353796.1 | -0.556 | 4.41E-58 |
| EEF1A1P7 | 0.572 | 4.15E-62 | AC011008.1 | -0.556 | 4.11E-58 |
| RPA3 | 0.572 | 4.38E-62 | ABLIM1 | -0.556 | 3.28E-58 |
| P3H4 | 0.572 | 4.44E-62 | SMPD3 | -0.556 | 3.27E-58 |
| TRIP10 | 0.572 | 4.50E-62 | SATB1 | -0.557 | 2.62E-58 |
| TXLNB | 0.572 | 4.81E-62 | GRIN2C | -0.557 | 2.21E-58 |
| AL390755.1 | 0.572 | 4.82E-62 | ATP9A | -0.557 | 1.72E-58 |
| DYNLL1P1 | 0.572 | 4.82E-62 | MYH15 | -0.558 | 1.56E-58 |
| SKA1 | 0.571 | 5.19E-62 | SH3GL3 | -0.558 | 1.34E-58 |
| RPS7P4 | 0.571 | 5.27E-62 | KANTR | -0.558 | 1.05E-58 |
| GAPDHP71 | 0.571 | 5.77E-62 | SLC25A27 | -0.559 | 8.10E-59 |
| FUCA1 | 0.571 | 5.88E-62 | AC016931.1 | -0.559 | 7.98E-59 |
| TMEM147 | 0.571 | 6.31E-62 | PPP1R3F | -0.559 | 7.47E-59 |
| KTI12 | 0.571 | 6.50E-62 | CALN1 | -0.559 | 7.00E-59 |
| AC078817.1 | 0.571 | 6.60E-62 | LINC00609 | -0.559 | 5.92E-59 |
| ABHD17AP1 | 0.571 | 6.84E-62 | LINC00632 | -0.559 | 5.49E-59 |
| PLEKHA4 | 0.571 | 6.88E-62 | MAILR | -0.560 | 4.74E-59 |
| MIR1244-2 | 0.571 | 7.19E-62 | SEMA3D | -0.560 | 4.10E-59 |
| SLC12A7 | 0.571 | 7.38E-62 | RNA5SP508 | -0.561 | 2.85E-59 |
| CXCL8 | 0.571 | 7.93E-62 | ZNF25 | -0.561 | 2.54E-59 |
| SEPHS2 | 0.571 | 7.94E-62 | AL512308.1 | -0.561 | 2.42E-59 |
| HK3 | 0.571 | 7.97E-62 | ARFGEF3 | -0.561 | 2.10E-59 |
| C14orf119 | 0.571 | 8.21E-62 | DHTKD1 | -0.562 | 1.55E-59 |
| TRIP13 | 0.571 | 8.73E-62 | WDFY3-AS2 | -0.562 | 1.46E-59 |
| EXT2 | 0.571 | 8.83E-62 | WASF3 | -0.562 | 1.11E-59 |
| CHEK2 | 0.571 | 8.87E-62 | SLC1A6 | -0.562 | 1.06E-59 |
| PTPN12 | 0.571 | 8.89E-62 | TDH | -0.563 | 8.05E-60 |
| TXNDC15 | 0.571 | 8.92E-62 | ZHX2 | -0.564 | 5.40E-60 |
| KRT18 | 0.571 | 9.05E-62 | AC079414.3 | -0.564 | 5.18E-60 |
| FTH1P8 | 0.570 | 9.81E-62 | NRXN2 | -0.564 | 4.36E-60 |
| LINC01503 | 0.570 | 1.00E-61 | MAPK10 | -0.564 | 4.05E-60 |
| EEF1A1P14 | 0.570 | 1.00E-61 | MDGA2 | -0.564 | 3.86E-60 |
| HSP90AB3P | 0.570 | 1.11E-61 | SSTR2 | -0.564 | 3.47E-60 |
| HOXA1 | 0.570 | 1.16E-61 | LUZP2 | -0.564 | 3.46E-60 |
| HOXB7 | 0.570 | 1.31E-61 | TPPP | -0.565 | 2.76E-60 |
| TRAF5 | 0.570 | 1.49E-61 | RNF165 | -0.566 | 1.55E-60 |
| DUSP23 | 0.569 | 1.65E-61 | TOM1L2 | -0.566 | 1.27E-60 |
| AL133260.1 | 0.569 | 1.69E-61 | YPEL4 | -0.566 | 1.27E-60 |
| NAMPTP1 | 0.569 | 1.88E-61 | RBM17 | -0.566 | 9.75E-61 |
| SLC39A8 | 0.569 | 1.99E-61 | CASKIN1 | -0.567 | 9.16E-61 |
| AC092597.1 | 0.569 | 2.17E-61 | VSTM2B | -0.567 | 8.69E-61 |
| ELF4 | 0.569 | 2.25E-61 | HDAC11 | -0.567 | 6.41E-61 |
| FGFRL1 | 0.569 | 2.29E-61 | FRMPD3 | -0.568 | 4.89E-61 |
| EN1 | 0.569 | 2.30E-61 | TAS2R4 | -0.569 | 2.82E-61 |
| TMEM50A | 0.569 | 2.41E-61 | PLCL1 | -0.569 | 2.45E-61 |
| DTYMK | 0.569 | 2.48E-61 | CLASP2 | -0.569 | 2.28E-61 |
| LDHAP7 | 0.569 | 2.52E-61 | SGSM1 | -0.569 | 2.28E-61 |
| AC008753.1 | 0.569 | 2.58E-61 | AC073389.3 | -0.569 | 2.26E-61 |
| CDC42EP3 | 0.569 | 2.62E-61 | CPB2-AS1 | -0.569 | 1.79E-61 |
| ALG5 | 0.569 | 2.75E-61 | OLMALINC | -0.569 | 1.76E-61 |
| FCGR2A | 0.568 | 2.97E-61 | TAF3 | -0.570 | 1.45E-61 |
| SURF4 | 0.568 | 3.32E-61 | UNC5A | -0.570 | 1.24E-61 |
| HSPA6 | 0.568 | 3.46E-61 | LINC02593 | -0.570 | 1.23E-61 |
| RPS23P8 | 0.568 | 3.55E-61 | RASGEF1C | -0.570 | 1.20E-61 |
| C1QTNF6 | 0.568 | 3.60E-61 | LDHD | -0.570 | 1.10E-61 |
| CKAP4 | 0.568 | 3.82E-61 | CDNF | -0.571 | 8.78E-62 |
| FCGBP | 0.568 | 3.91E-61 | SLC16A9 | -0.571 | 6.20E-62 |
| RHOC | 0.568 | 4.22E-61 | GABRB3 | -0.571 | 5.29E-62 |
| SMC4 | 0.568 | 4.48E-61 | ZNNT1 | -0.572 | 4.03E-62 |
| TP53I13 | 0.568 | 4.89E-61 | TAFA5 | -0.572 | 2.97E-62 |
| SPINK8 | 0.568 | 4.92E-61 | TNRC6C | -0.573 | 2.49E-62 |
| FABP7 | 0.568 | 4.92E-61 | AC018730.1 | -0.573 | 2.47E-62 |
| CHRNA1 | 0.568 | 5.29E-61 | INA | -0.573 | 2.45E-62 |
| GAPDH | 0.567 | 5.45E-61 | ADARB2 | -0.573 | 2.43E-62 |
| TMEM60 | 0.567 | 5.89E-61 | MN1 | -0.573 | 1.54E-62 |
| HMGN1P36 | 0.567 | 6.75E-61 | GDPD1 | -0.574 | 1.49E-62 |
| FTH1P16 | 0.567 | 8.72E-61 | MTSS2 | -0.574 | 1.48E-62 |
| ADGRE2 | 0.567 | 9.00E-61 | RAP2A | -0.574 | 1.23E-62 |
| HM13 | 0.566 | 1.12E-60 | OBI1-AS1 | -0.574 | 1.10E-62 |
| OTP | 0.566 | 1.16E-60 | ALCAM | -0.574 | 9.51E-63 |
| RTCA | 0.566 | 1.18E-60 | HMX1 | -0.574 | 8.68E-63 |
| YWHAEP1 | 0.566 | 1.18E-60 | NECAB2 | -0.575 | 7.53E-63 |
| RPS3AP26 | 0.566 | 1.21E-60 | TEF | -0.575 | 7.17E-63 |
| RPL13AP5 | 0.566 | 1.36E-60 | ASIC4-AS1 | -0.575 | 7.06E-63 |
| NDUFA5P11 | 0.566 | 1.38E-60 | FERMT1 | -0.575 | 5.63E-63 |
| AC046143.1 | 0.566 | 1.39E-60 | BCL7A | -0.575 | 5.00E-63 |
| ZNF701 | 0.566 | 1.43E-60 | TRAPPC13P1 | -0.576 | 3.04E-63 |
| AC104563.1 | 0.566 | 1.47E-60 | PRLHR | -0.576 | 3.01E-63 |
| PSMC4 | 0.566 | 1.47E-60 | STARD10 | -0.576 | 2.71E-63 |
| ARMC10 | 0.566 | 1.49E-60 | KCNJ10 | -0.576 | 2.69E-63 |
| BCAM | 0.566 | 1.59E-60 | AC091271.1 | -0.577 | 2.28E-63 |
| ZNF236-DT | 0.566 | 1.69E-60 | PPP1R1A | -0.577 | 1.37E-63 |
| C1GALT1C1 | 0.566 | 1.70E-60 | NSUN6 | -0.578 | 1.33E-63 |
| PPP1R15A | 0.566 | 1.70E-60 | ZNF248 | -0.578 | 1.17E-63 |
| DSG2 | 0.565 | 1.87E-60 | AL512343.2 | -0.578 | 1.14E-63 |
| DNAJB11 | 0.565 | 1.92E-60 | DISP3 | -0.578 | 1.03E-63 |
| CCNB2 | 0.565 | 1.92E-60 | AC062021.1 | -0.579 | 7.39E-64 |
| RPSAP54 | 0.565 | 2.00E-60 | AC092117.1 | -0.579 | 4.05E-64 |
| ARPC3 | 0.565 | 2.06E-60 | RHBDL3 | -0.580 | 3.70E-64 |
| MT2P1 | 0.565 | 2.31E-60 | OPHN1 | -0.580 | 3.36E-64 |
| TIFA | 0.565 | 2.58E-60 | LINC00836 | -0.580 | 3.28E-64 |
| RPL26P19 | 0.565 | 2.83E-60 | AL117339.3 | -0.580 | 2.52E-64 |
| ACTG1P20 | 0.564 | 3.21E-60 | AC110285.1 | -0.581 | 2.03E-64 |
| GJD3 | 0.564 | 3.50E-60 | ADHFE1 | -0.581 | 1.58E-64 |
| RAI14 | 0.564 | 3.53E-60 | FSD1L | -0.581 | 1.57E-64 |
| BANF1P3 | 0.564 | 3.71E-60 | PLCB1 | -0.581 | 1.56E-64 |
| HOXB4 | 0.564 | 3.75E-60 | ITPK1 | -0.581 | 1.38E-64 |
| ITGB1BP1 | 0.564 | 3.77E-60 | NKD1 | -0.581 | 1.36E-64 |
| BST1 | 0.564 | 3.78E-60 | PAK5 | -0.581 | 1.33E-64 |
| DENND2D | 0.564 | 3.87E-60 | ABHD6 | -0.581 | 1.31E-64 |
| C8orf76 | 0.564 | 4.08E-60 | SCD | -0.582 | 1.16E-64 |
| AC026271.1 | 0.564 | 4.39E-60 | TLCD5 | -0.582 | 1.10E-64 |
| RPL23AP18 | 0.564 | 4.40E-60 | GDAP1L1 | -0.582 | 7.44E-65 |
| RWDD4P2 | 0.564 | 4.54E-60 | MAPK8IP2 | -0.583 | 5.95E-65 |
| OAF | 0.564 | 5.17E-60 | GNAL | -0.583 | 4.98E-65 |
| NPC2 | 0.564 | 5.32E-60 | SSTR1 | -0.583 | 4.35E-65 |
| ZC3HAV1L | 0.564 | 5.39E-60 | AL133304.3 | -0.584 | 3.07E-65 |
| FAP | 0.563 | 6.73E-60 | DAAM2 | -0.584 | 2.51E-65 |
| RBPMS | 0.563 | 7.03E-60 | JPH4 | -0.584 | 2.40E-65 |
| EIF3I | 0.563 | 7.13E-60 | AL034348.1 | -0.584 | 2.37E-65 |
| RPSAP4 | 0.563 | 7.65E-60 | SCG3 | -0.584 | 2.10E-65 |
| LY96 | 0.563 | 8.03E-60 | CADM2 | -0.584 | 1.99E-65 |
| ZDHHC18 | 0.563 | 8.12E-60 | AP006333.2 | -0.584 | 1.90E-65 |
| ENPP1 | 0.563 | 8.19E-60 | TLCD3B | -0.585 | 1.75E-65 |
| TTC26 | 0.563 | 8.45E-60 | TNK2 | -0.585 | 1.62E-65 |
| HJURP | 0.563 | 8.53E-60 | CDIP1 | -0.585 | 1.42E-65 |
| AC012618.1 | 0.563 | 8.64E-60 | HDAC4 | -0.585 | 1.35E-65 |
| LPAR6 | 0.563 | 9.34E-60 | RTN3 | -0.585 | 1.35E-65 |
| FAM83D | 0.563 | 9.38E-60 | AC027130.1 | -0.585 | 1.35E-65 |
| RPSAP9 | 0.563 | 9.52E-60 | AL049838.1 | -0.585 | 1.12E-65 |
| CARD19 | 0.562 | 1.00E-59 | ARL3 | -0.586 | 9.51E-66 |
| ADSS1 | 0.562 | 1.02E-59 | PTPRT | -0.586 | 9.41E-66 |
| RAB36 | 0.562 | 1.04E-59 | CHRNA4 | -0.586 | 7.83E-66 |
| RPL35P5 | 0.562 | 1.04E-59 | NSG2 | -0.586 | 5.94E-66 |
| DUSP4 | 0.562 | 1.06E-59 | PDE2A | -0.588 | 2.08E-66 |
| RELB | 0.562 | 1.07E-59 | PLA2G6 | -0.588 | 2.06E-66 |
| SUMF1 | 0.562 | 1.08E-59 | TSPOAP1 | -0.589 | 1.32E-66 |
| PSMA5 | 0.562 | 1.09E-59 | ATOH8 | -0.589 | 1.07E-66 |
| TYMP | 0.562 | 1.10E-59 | FAAH | -0.590 | 7.15E-67 |
| RPS3AP49 | 0.562 | 1.10E-59 | RPS6KA5 | -0.591 | 4.17E-67 |
| RPLP0P6 | 0.562 | 1.19E-59 | MT-RNR1 | -0.591 | 3.20E-67 |
| ITPKC | 0.562 | 1.32E-59 | C14orf132 | -0.591 | 3.17E-67 |
| GALNT2 | 0.562 | 1.39E-59 | MT-TT | -0.591 | 3.10E-67 |
| SEC22B | 0.562 | 1.40E-59 | GRIK4 | -0.591 | 2.45E-67 |
| CA3 | 0.562 | 1.45E-59 | TMEM254-AS1 | -0.591 | 2.39E-67 |
| ATP5MFP2 | 0.562 | 1.64E-59 | AKAP6 | -0.592 | 2.18E-67 |
| SRP9P1 | 0.562 | 1.65E-59 | DNAJC27-AS1 | -0.592 | 2.10E-67 |
| REEP4 | 0.562 | 1.65E-59 | HIPK2 | -0.592 | 1.79E-67 |
| RPS2P46 | 0.562 | 1.68E-59 | ZC3H12B | -0.592 | 1.74E-67 |
| ACTA2 | 0.561 | 1.84E-59 | PEA15 | -0.592 | 1.56E-67 |
| LINC01579 | 0.561 | 1.97E-59 | EPHB1 | -0.592 | 1.29E-67 |
| CDCP1 | 0.561 | 2.06E-59 | ANKRD46 | -0.592 | 1.22E-67 |
| RPS3AP5 | 0.561 | 2.11E-59 | ADGRA1-AS1 | -0.593 | 1.11E-67 |
| EVC | 0.561 | 2.36E-59 | ZNF483 | -0.593 | 8.96E-68 |
| GPR65 | 0.561 | 2.82E-59 | AC133637.1 | -0.593 | 7.90E-68 |
| AC090114.1 | 0.561 | 2.93E-59 | PCBP3 | -0.594 | 5.58E-68 |
| AL353807.5 | 0.560 | 3.09E-59 | BCR | -0.594 | 4.92E-68 |
| SLC25A19 | 0.560 | 3.11E-59 | ADCY5 | -0.594 | 3.52E-68 |
| HCCS | 0.560 | 3.14E-59 | AC007342.7 | -0.596 | 1.38E-68 |
| STIL | 0.560 | 3.22E-59 | USH1C | -0.596 | 1.34E-68 |
| RPL19P5 | 0.560 | 3.37E-59 | VIPR2 | -0.596 | 1.16E-68 |
| Z97353.1 | 0.560 | 3.42E-59 | AL731533.2 | -0.596 | 9.95E-69 |
| EEF1A1P24 | 0.560 | 3.93E-59 | AL158212.3 | -0.597 | 8.59E-69 |
| DPEP1 | 0.560 | 3.98E-59 | RICTOR | -0.597 | 8.07E-69 |
| F13A1 | 0.560 | 3.98E-59 | SPHKAP | -0.597 | 7.29E-69 |
| NMI | 0.560 | 3.99E-59 | FOXO4 | -0.597 | 5.94E-69 |
| S100A16 | 0.560 | 4.24E-59 | TRIM8 | -0.598 | 2.78E-69 |
| RGS3 | 0.560 | 4.45E-59 | NTNG2 | -0.599 | 2.29E-69 |
| SERPINF1 | 0.560 | 4.69E-59 | CXXC4 | -0.599 | 1.68E-69 |
| PROS1 | 0.560 | 4.75E-59 | AL355334.2 | -0.599 | 1.32E-69 |
| ALG2 | 0.560 | 4.77E-59 | ZBTB18 | -0.600 | 1.08E-69 |
| NANP | 0.560 | 5.02E-59 | AC074286.1 | -0.601 | 6.45E-70 |
| TMED3 | 0.559 | 5.80E-59 | PCDH15 | -0.601 | 4.97E-70 |
| VNN2 | 0.559 | 6.04E-59 | SLITRK5 | -0.601 | 4.67E-70 |
| CHST14 | 0.559 | 6.11E-59 | RNU6-529P | -0.602 | 3.35E-70 |
| CCZ1B | 0.559 | 6.46E-59 | CEROX1 | -0.602 | 2.93E-70 |
| AL136116.3 | 0.559 | 6.56E-59 | USP54 | -0.602 | 2.70E-70 |
| SLC44A3-AS1 | 0.559 | 6.57E-59 | ZBTB47 | -0.602 | 2.26E-70 |
| LILRB3 | 0.559 | 6.57E-59 | ACBD5 | -0.602 | 1.85E-70 |
| SLC25A45 | 0.559 | 6.60E-59 | FCHSD2 | -0.603 | 1.71E-70 |
| TPI1 | 0.559 | 6.70E-59 | ETNPPL | -0.603 | 1.62E-70 |
| E2F8 | 0.559 | 6.70E-59 | SAMD8 | -0.603 | 1.31E-70 |
| AC009961.3 | 0.559 | 6.83E-59 | SPTBN2 | -0.603 | 1.03E-70 |
| LEF1 | 0.559 | 7.12E-59 | AL157392.3 | -0.605 | 3.55E-71 |
| EEF1DP1 | 0.559 | 7.21E-59 | OTUD7A | -0.606 | 1.66E-71 |
| AC127502.2 | 0.559 | 7.31E-59 | SLC22A6 | -0.606 | 1.54E-71 |
| IMPA2 | 0.559 | 7.37E-59 | RAB11FIP4 | -0.607 | 1.14E-71 |
| SECTM1 | 0.559 | 8.72E-59 | SLC25A48 | -0.607 | 9.98E-72 |
| DYRK3 | 0.559 | 8.79E-59 | GABRG1 | -0.607 | 9.89E-72 |
| RPL10P16 | 0.559 | 9.09E-59 | MCF2L2 | -0.607 | 6.64E-72 |
| PGM2 | 0.559 | 9.53E-59 | KIFBP | -0.608 | 5.28E-72 |
| EEF1A1P4 | 0.558 | 9.93E-59 | SHISA7 | -0.608 | 3.94E-72 |
| AC112220.2 | 0.558 | 1.12E-58 | DLL1 | -0.608 | 3.88E-72 |
| RPL14P1 | 0.558 | 1.13E-58 | ADAM22 | -0.609 | 2.52E-72 |
| FAM136A | 0.558 | 1.14E-58 | IGIP | -0.609 | 1.89E-72 |
| TNFRSF18 | 0.558 | 1.22E-58 | RUNDC3A | -0.609 | 1.78E-72 |
| PTGR1 | 0.558 | 1.25E-58 | LINC00641 | -0.609 | 1.76E-72 |
| IFRD2 | 0.558 | 1.30E-58 | TNR | -0.610 | 8.09E-73 |
| COMMD10 | 0.558 | 1.40E-58 | IFITM10 | -0.611 | 6.08E-73 |
| AC092645.1 | 0.558 | 1.45E-58 | FBXW4 | -0.611 | 5.26E-73 |
| NCAPG | 0.558 | 1.52E-58 | PAIP2B | -0.611 | 5.11E-73 |
| CTBS | 0.558 | 1.53E-58 | AC005696.4 | -0.612 | 4.04E-73 |
| KCTD14 | 0.558 | 1.54E-58 | ASB13 | -0.612 | 3.09E-73 |
| ERCC6L | 0.558 | 1.69E-58 | KCNJ9 | -0.612 | 2.39E-73 |
| AC005480.2 | 0.557 | 1.73E-58 | SHANK2 | -0.612 | 2.23E-73 |
| PMEPA1 | 0.557 | 2.09E-58 | SHD | -0.613 | 1.77E-73 |
| AC016739.1 | 0.557 | 2.29E-58 | AC005070.3 | -0.613 | 1.74E-73 |
| EEF1A1P29 | 0.557 | 2.32E-58 | LRIT2 | -0.613 | 1.51E-73 |
| FPGT | 0.557 | 2.42E-58 | MTURN | -0.613 | 1.06E-73 |
| SUMF2 | 0.557 | 2.52E-58 | AC004803.1 | -0.613 | 1.05E-73 |
| RPS3AP21 | 0.557 | 2.52E-58 | KIF21B | -0.614 | 6.57E-74 |
| RPS29P16 | 0.557 | 2.69E-58 | CMTM5 | -0.614 | 5.94E-74 |
| EEF1A1P10 | 0.557 | 2.77E-58 | FLRT1 | -0.615 | 4.75E-74 |
| BCL7B | 0.557 | 2.83E-58 | CBLN1 | -0.615 | 2.82E-74 |
| AC087385.2 | 0.556 | 3.17E-58 | MGAT4C | -0.616 | 2.22E-74 |
| RPL29P12 | 0.556 | 3.38E-58 | ZNF488 | -0.616 | 1.83E-74 |
| AL162231.2 | 0.556 | 3.44E-58 | AL359091.1 | -0.616 | 1.80E-74 |
| AC091825.2 | 0.556 | 3.47E-58 | FAIM2 | -0.617 | 8.28E-75 |
| CTSZ | 0.556 | 3.47E-58 | CCSER2 | -0.618 | 6.11E-75 |
| MRPS18A | 0.556 | 3.56E-58 | AP000223.1 | -0.618 | 3.42E-75 |
| GPR89A | 0.556 | 3.62E-58 | CSDC2 | -0.618 | 3.24E-75 |
| HS2ST1 | 0.556 | 3.72E-58 | MARCHF8 | -0.619 | 2.88E-75 |
| ITGB3BP | 0.556 | 4.15E-58 | OMG | -0.619 | 2.00E-75 |
| AP000936.3 | 0.556 | 4.33E-58 | SLCO1A2 | -0.619 | 1.77E-75 |
| CD3D | 0.556 | 4.36E-58 | NOG | -0.620 | 1.13E-75 |
| CD2 | 0.556 | 4.37E-58 | ATP8A1 | -0.620 | 9.79E-76 |
| TMEM87B | 0.556 | 4.91E-58 | ADCY2 | -0.620 | 9.35E-76 |
| ADAMTS1 | 0.556 | 5.17E-58 | SOX8 | -0.621 | 6.76E-76 |
| SMIM12 | 0.556 | 5.22E-58 | AC097382.3 | -0.621 | 5.69E-76 |
| MELK | 0.555 | 5.45E-58 | ZMYND11 | -0.622 | 2.98E-76 |
| PCLAF | 0.555 | 5.48E-58 | PDZD4 | -0.622 | 1.98E-76 |
| HSPA8P1 | 0.555 | 5.60E-58 | AC120036.4 | -0.623 | 1.85E-76 |
| IRF1 | 0.555 | 5.78E-58 | TARS3 | -0.623 | 1.36E-76 |
| CCDC125 | 0.555 | 6.01E-58 | RPRM | -0.623 | 9.87E-77 |
| CD48 | 0.555 | 6.11E-58 | FRY | -0.624 | 8.21E-77 |
| IRAK2 | 0.555 | 6.40E-58 | NALCN | -0.624 | 6.96E-77 |
| ACOT9 | 0.555 | 7.29E-58 | WAC-AS1 | -0.624 | 6.56E-77 |
| HSP90B1 | 0.555 | 7.38E-58 | GLUD1 | -0.624 | 6.34E-77 |
| WDR1 | 0.555 | 7.65E-58 | DPP10 | -0.624 | 5.75E-77 |
| KIF4A | 0.554 | 9.39E-58 | RTP5 | -0.626 | 1.08E-77 |
| LILRA5 | 0.554 | 1.06E-57 | AL157700.1 | -0.627 | 7.48E-78 |
| RNASE2 | 0.554 | 1.19E-57 | CPEB3 | -0.628 | 4.20E-78 |
| CHSY3 | 0.554 | 1.30E-57 | ARPP21 | -0.628 | 3.61E-78 |
| PPIAP22 | 0.554 | 1.43E-57 | NCAM1 | -0.628 | 3.52E-78 |
| ALG1 | 0.554 | 1.51E-57 | DNAJC12 | -0.630 | 1.05E-78 |
| FTH1P5 | 0.554 | 1.52E-57 | CTNNA3 | -0.631 | 5.26E-79 |
| OLFML1 | 0.554 | 1.54E-57 | HES6 | -0.631 | 3.06E-79 |
| RNFT1 | 0.554 | 1.54E-57 | NTRK2 | -0.631 | 3.01E-79 |
| PTGS1 | 0.554 | 1.56E-57 | PHACTR3-AS1 | -0.632 | 1.45E-79 |
| AL445433.1 | 0.553 | 1.67E-57 | CSMD3 | -0.635 | 2.41E-80 |
| MBD2 | 0.553 | 1.78E-57 | ALDOC | -0.635 | 1.73E-80 |
| KANK2 | 0.553 | 1.93E-57 | TUB | -0.635 | 1.59E-80 |
| ALOX5AP | 0.553 | 2.16E-57 | TMCC2 | -0.638 | 2.95E-81 |
| RPL29P33 | 0.553 | 2.38E-57 | HPSE2 | -0.638 | 2.64E-81 |
| AL590135.1 | 0.553 | 2.44E-57 | MT-RNR2 | -0.639 | 7.72E-82 |
| FLOT1 | 0.553 | 2.46E-57 | FRA10AC1 | -0.640 | 4.68E-82 |
| HNRNPA1P7 | 0.553 | 2.61E-57 | AL592295.6 | -0.640 | 4.23E-82 |
| P4HA2 | 0.553 | 2.66E-57 | NET1 | -0.642 | 9.08E-83 |
| SELENON | 0.553 | 2.67E-57 | SNAP91 | -0.643 | 4.72E-83 |
| IER5 | 0.553 | 2.67E-57 | USP43 | -0.644 | 2.59E-83 |
| DYNC1I2P1 | 0.553 | 2.77E-57 | FAM222A | -0.646 | 4.56E-84 |
| CIBAR1P1 | 0.552 | 2.86E-57 | CTIF | -0.646 | 4.19E-84 |
| IL13RA2 | 0.552 | 2.88E-57 | ZDHHC22 | -0.646 | 3.88E-84 |
| IFITM2 | 0.552 | 2.95E-57 | CRY2 | -0.646 | 3.77E-84 |
| P2RY6 | 0.552 | 2.97E-57 | ALDH5A1 | -0.647 | 2.89E-84 |
| KLHL4 | 0.552 | 2.99E-57 | AMER2 | -0.647 | 1.81E-84 |
| NEIL3 | 0.552 | 3.08E-57 | AC015540.1 | -0.647 | 1.70E-84 |
| CXCL11 | 0.552 | 3.08E-57 | ATP6V1G2 | -0.649 | 5.58E-85 |
| GJB2 | 0.552 | 3.31E-57 | SEPTIN3 | -0.649 | 4.92E-85 |
| GRN | 0.552 | 3.50E-57 | GABBR1 | -0.649 | 4.66E-85 |
| HOXD13 | 0.552 | 3.50E-57 | AC009227.1 | -0.650 | 2.99E-85 |
| ACTG2 | 0.552 | 3.53E-57 | CDH20 | -0.650 | 2.19E-85 |
| PGK1P2 | 0.552 | 3.56E-57 | TCEAL2 | -0.650 | 1.73E-85 |
| TFRC | 0.552 | 4.58E-57 | REPS2 | -0.652 | 6.13E-86 |
| GRB10 | 0.552 | 4.67E-57 | PPM1L | -0.652 | 4.31E-86 |
| FAM86DP | 0.551 | 5.04E-57 | FAM171A1 | -0.653 | 2.46E-86 |
| HUS1 | 0.551 | 5.10E-57 | KIF1A | -0.654 | 1.02E-86 |
| F3 | 0.551 | 5.10E-57 | NRG3 | -0.654 | 8.38E-87 |
| PKM | 0.551 | 5.11E-57 | FLJ16779 | -0.654 | 7.29E-87 |
| NEK2 | 0.551 | 5.13E-57 | DNM3 | -0.655 | 5.05E-87 |
| POC1A | 0.551 | 5.43E-57 | MAPT-AS1 | -0.656 | 2.30E-87 |
| ATP5PDP4 | 0.551 | 5.78E-57 | AUXG01000058.1 | -0.657 | 9.10E-88 |
| HOXC9 | 0.551 | 5.80E-57 | RASL10A | -0.659 | 1.25E-88 |
| AL596087.1 | 0.551 | 5.81E-57 | CRTAC1 | -0.660 | 5.34E-89 |
| KIF2C | 0.551 | 6.07E-57 | DUSP26 | -0.661 | 3.77E-89 |
| AC133134.1 | 0.551 | 6.12E-57 | KLHL32 | -0.662 | 1.82E-89 |
| ARPC1A | 0.551 | 6.29E-57 | NAP1L3 | -0.662 | 9.86E-90 |
| DNAJC22 | 0.551 | 6.59E-57 | SMOC1 | -0.663 | 5.36E-90 |
| CAPG | 0.551 | 6.83E-57 | JPH3 | -0.663 | 5.14E-90 |
| CHI3L2 | 0.551 | 6.91E-57 | CDHR1 | -0.666 | 6.70E-91 |
| PDCD5 | 0.551 | 7.44E-57 | PC | -0.666 | 3.19E-91 |
| TMEM179B | 0.551 | 7.47E-57 | DSCAML1 | -0.669 | 4.33E-92 |
| AC112191.2 | 0.551 | 7.53E-57 | HMGN5 | -0.670 | 1.25E-92 |
| RPL37AP1 | 0.551 | 7.61E-57 | MIR9-3HG | -0.671 | 5.67E-93 |
| CHSY1 | 0.551 | 7.63E-57 | GALNT13 | -0.672 | 2.32E-93 |
| AC018475.1 | 0.551 | 7.88E-57 | AL118505.1 | -0.674 | 4.97E-94 |
| FBXO22 | 0.551 | 8.14E-57 | ALDH2 | -0.676 | 1.34E-94 |
| BCL10 | 0.551 | 8.46E-57 | GNAO1 | -0.676 | 1.32E-94 |
| YWHAZP2 | 0.551 | 8.48E-57 | MXI1 | -0.677 | 5.57E-95 |
| SLC35D1 | 0.550 | 8.75E-57 | FAM133A | -0.678 | 1.29E-95 |
| TNFRSF10D | 0.550 | 9.04E-57 | ENHO | -0.680 | 4.00E-96 |
| POLR1F | 0.550 | 9.15E-57 | FAM13C | -0.680 | 3.48E-96 |
| ECHDC2 | 0.550 | 1.08E-56 | LINC00844 | -0.680 | 2.00E-96 |
| MYL12BP1 | 0.550 | 1.09E-56 | FUT9 | -0.681 | 7.33E-97 |
| FTH1P20 | 0.550 | 1.10E-56 | SH3GL2 | -0.683 | 2.79E-97 |
| SLC35D2 | 0.550 | 1.13E-56 | SLC25A21-AS1 | -0.683 | 1.42E-97 |
| CMYA5 | 0.550 | 1.25E-56 | NDRG2 | -0.684 | 8.72E-98 |
| PCCA-DT | 0.550 | 1.36E-56 | KCNIP3 | -0.697 | 6.36E-103 |
| GALM | 0.550 | 1.37E-56 | MAPT | -0.697 | 4.84E-103 |
| SNRPEP4 | 0.550 | 1.41E-56 | KCNB1 | -0.701 | 1.43E-104 |
| VTRNA1-2 | 0.549 | 1.53E-56 | PHACTR3 | -0.713 | 1.13E-109 |
| MYOF | 0.549 | 1.61E-56 | LINC00634 | -0.718 | 3.52E-112 |
| RPL9P32 | 0.549 | 1.82E-56 | THRA | -0.728 | 1.50E-116 |
| CMTM3 | 0.549 | 1.91E-56 | PHYHIPL | -0.734 | 1.76E-119 |
| MTFR2 | 0.549 | 2.14E-56 |  |  |  |
